# Supplementary material for: Targeting HPK1 inhibits neutrophil responses to mitigate post-stroke lung and cerebral injuries
Source: EMBO Mol Med. 2025 Apr 1;17(5):1018–40. doi: 10.1038/s44321-025-00220-8 (PMC12081623; doi:10.1038/s44321-025-00220-8)
Supplement: Supplementary file 1 — Appendix [file 44321_2025_220_MOESM1_ESM.pdf]

Appendix

Targeting HPK1 inhibits neutrophil responses to mitigate post-stroke lung and cerebral injuries

Tingting Zhang, Ying Sun, Jing Xia, Hongye Fan, Dingfang Shi, Qian Wu, Ming Huang, Xiao-Yu Hou

Table of Contents：

Appendix Table S1. Primers for qPCR in this study …………………………… Page 2

Appendix Figure S1. Gating strategies for neutrophil or microglia analysis in flow cytometry……………………………………………………………...……... Page 3

Appendix Table S1. Primers for qPCR in this study

Gene	Sense primer	Antisense primer
Tnf	TCTCATGCACCACCATCAAGGACT	ACCACTCTCCCTTTGCAGAACTCA
Il6	ACTTCCATCCAGTTGCCTTCTTGG	TTAAGCCTCCGACTTGTGAAGTGG
Il1b	TCGCAGCAGCACATCAACAAGAG	TGCTCATGTCCTCATCCTGGAAGG
Nos2	GGAGTGACGGCAAACATGACT	TCGATGCACAACTGGGTGAAC
Ptgs2	CACTCTATCACTGGCACCCC	TTGGCACATTTCTTCCCCCA
Socs3	GCACCTTTCTTATCCGCGAC	CTCACACTGGATGCGTAGGT
Il10	CAGTGGAGCAGGTGAAGAGT	AGATGTCAAATTCATTCATGGCCT

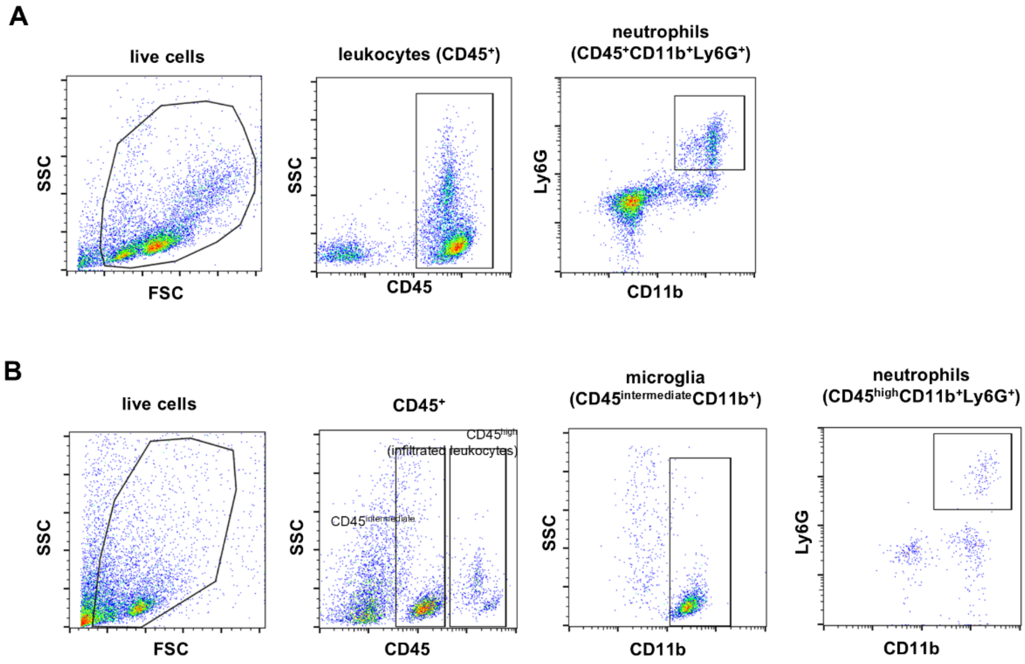

Appendix Figure S1. Gating strategies for neutrophil or microglia analysis in flow cytometry

(A) The gating strategy for neutrophil population ($CD45^+CD11b^+Ly6G^+$) in the blood, bone marrow, lung, and spleen. **(B)** The gating strategy for brain infiltrated neutrophils ($CD45^{high}CD11b^+Ly6G^+$) and microglia ($CD45^{intermediate}CD11b^+$).
