# Supplementary figures and images for: Targeting HPK1 inhibits neutrophil responses to mitigate post-stroke lung and cerebral injuries
Source: EMBO Mol Med. 2025 Apr 1;17(5):1018–40. doi: 10.1038/s44321-025-00220-8 (PMC12081623; doi:10.1038/s44321-025-00220-8)

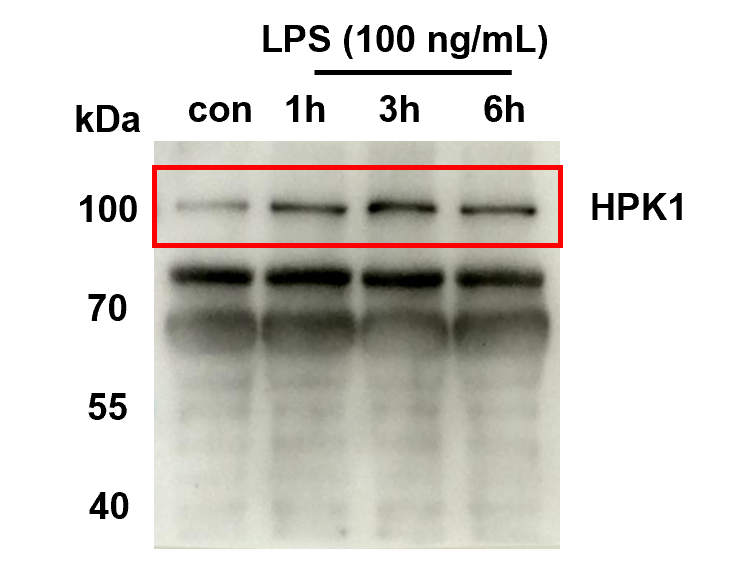

Supplement: Supplementary file 3 — EV Figures Source Data [file 44321_2025_220_MOESM3_ESM.zip › EMM-2024-20638-V3_EV Figures/Figure EV1/Figure EV1A/HPK1.tif]

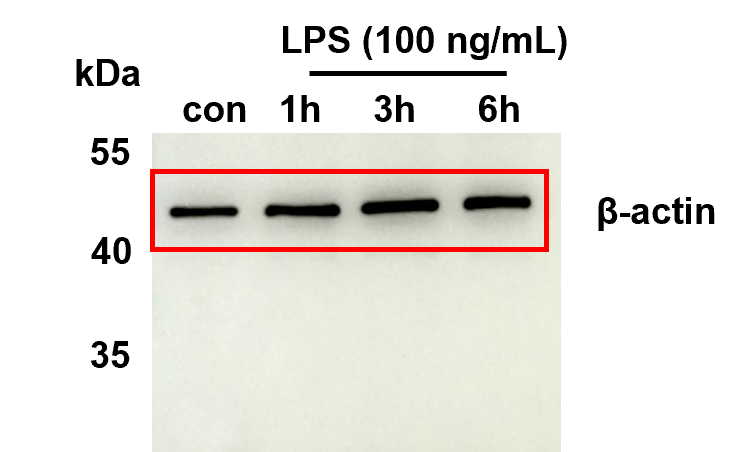

Supplement: Supplementary file 3 — EV Figures Source Data [file 44321_2025_220_MOESM3_ESM.zip › EMM-2024-20638-V3_EV Figures/Figure EV1/Figure EV1A/β-actin.tif]

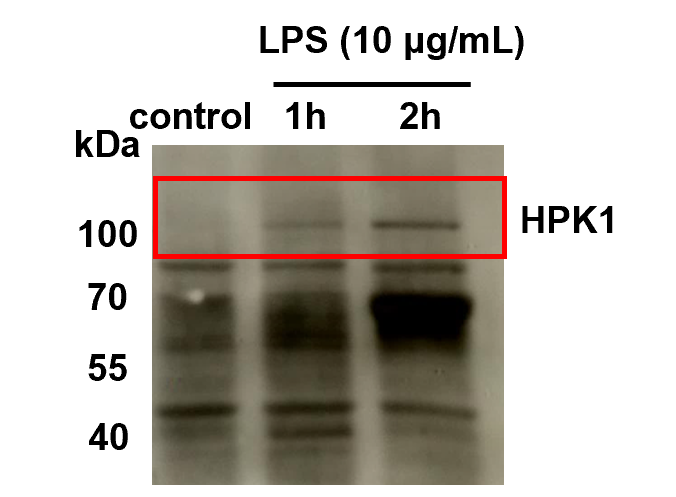

Supplement: Supplementary file 3 — EV Figures Source Data [file 44321_2025_220_MOESM3_ESM.zip › EMM-2024-20638-V3_EV Figures/Figure EV1/Figure EV1C/HPK1.png.tif]

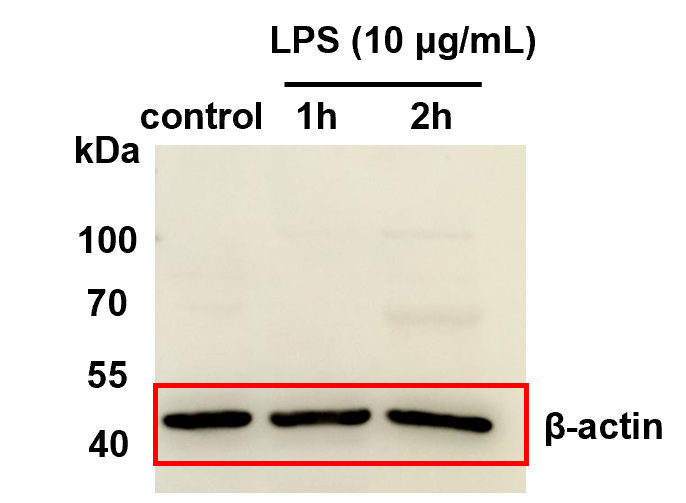

Supplement: Supplementary file 3 — EV Figures Source Data [file 44321_2025_220_MOESM3_ESM.zip › EMM-2024-20638-V3_EV Figures/Figure EV1/Figure EV1C/β-actin.tif]

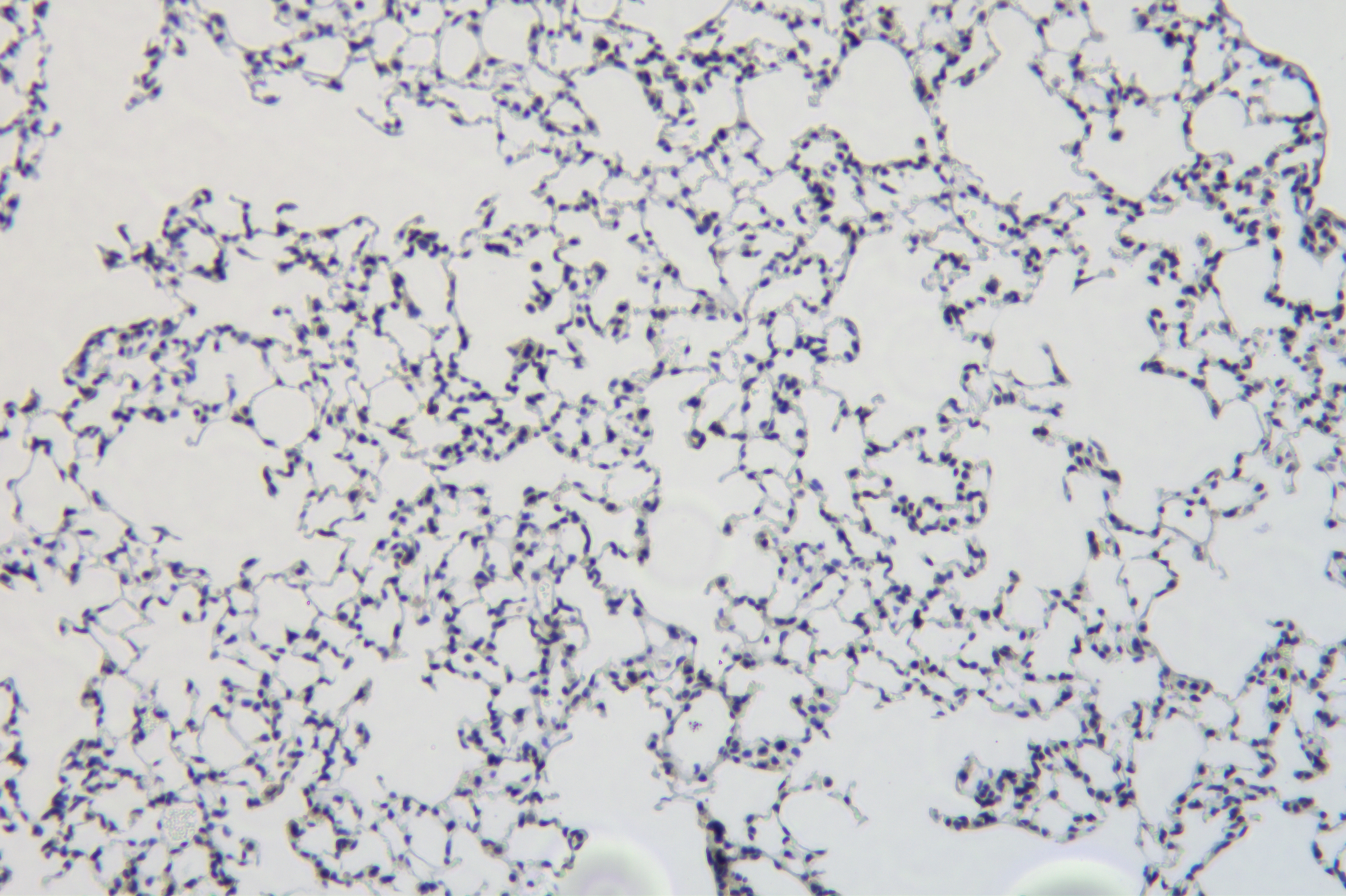

Supplement: Supplementary file 3 — EV Figures Source Data [file 44321_2025_220_MOESM3_ESM.zip › EMM-2024-20638-V3_EV Figures/Figure EV3/Figure EV3A/KO sham/KO sham CitH3.jpg]

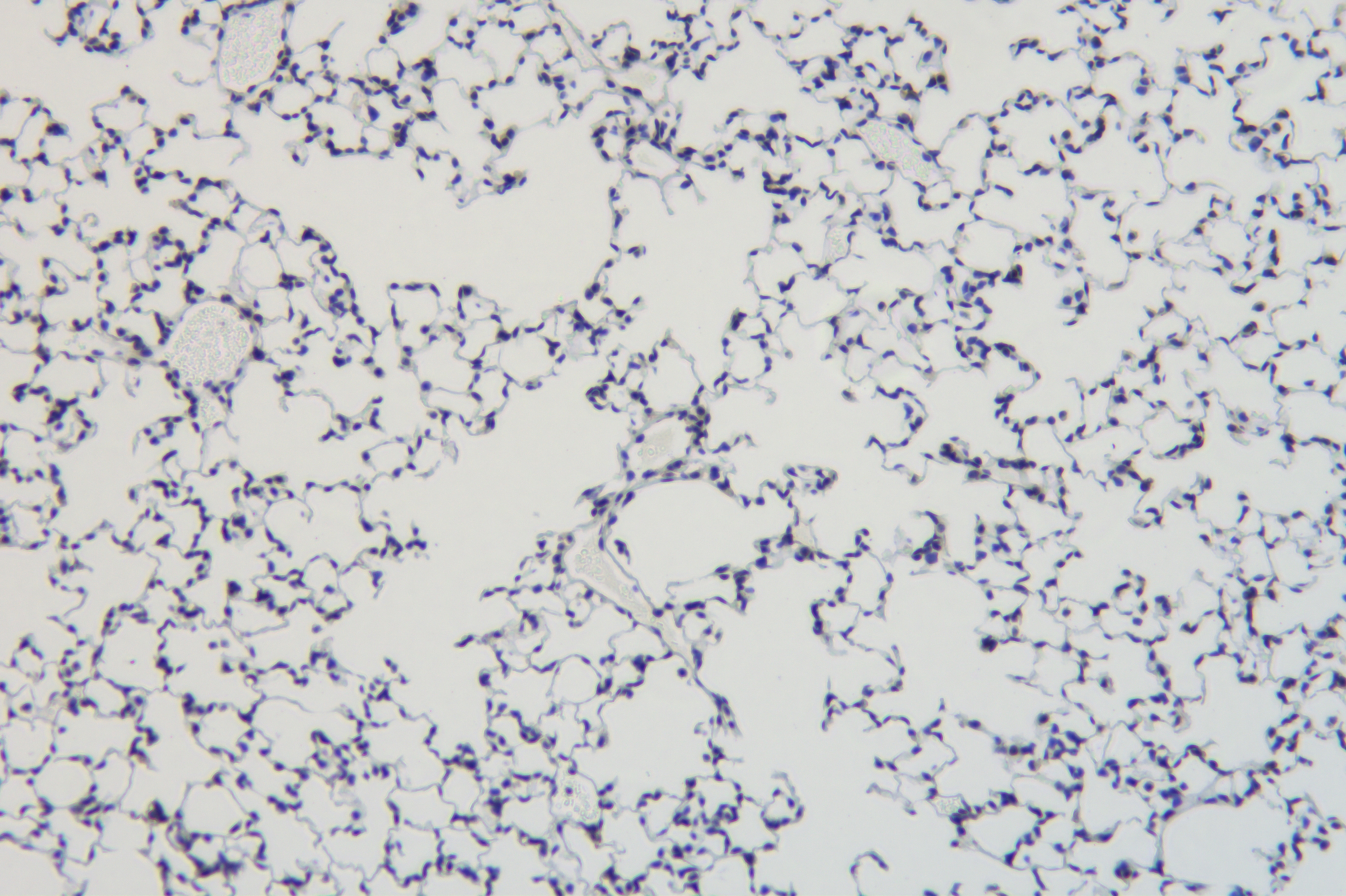

Supplement: Supplementary file 3 — EV Figures Source Data [file 44321_2025_220_MOESM3_ESM.zip › EMM-2024-20638-V3_EV Figures/Figure EV3/Figure EV3A/WT sham/WT sham CitH3.jpg]

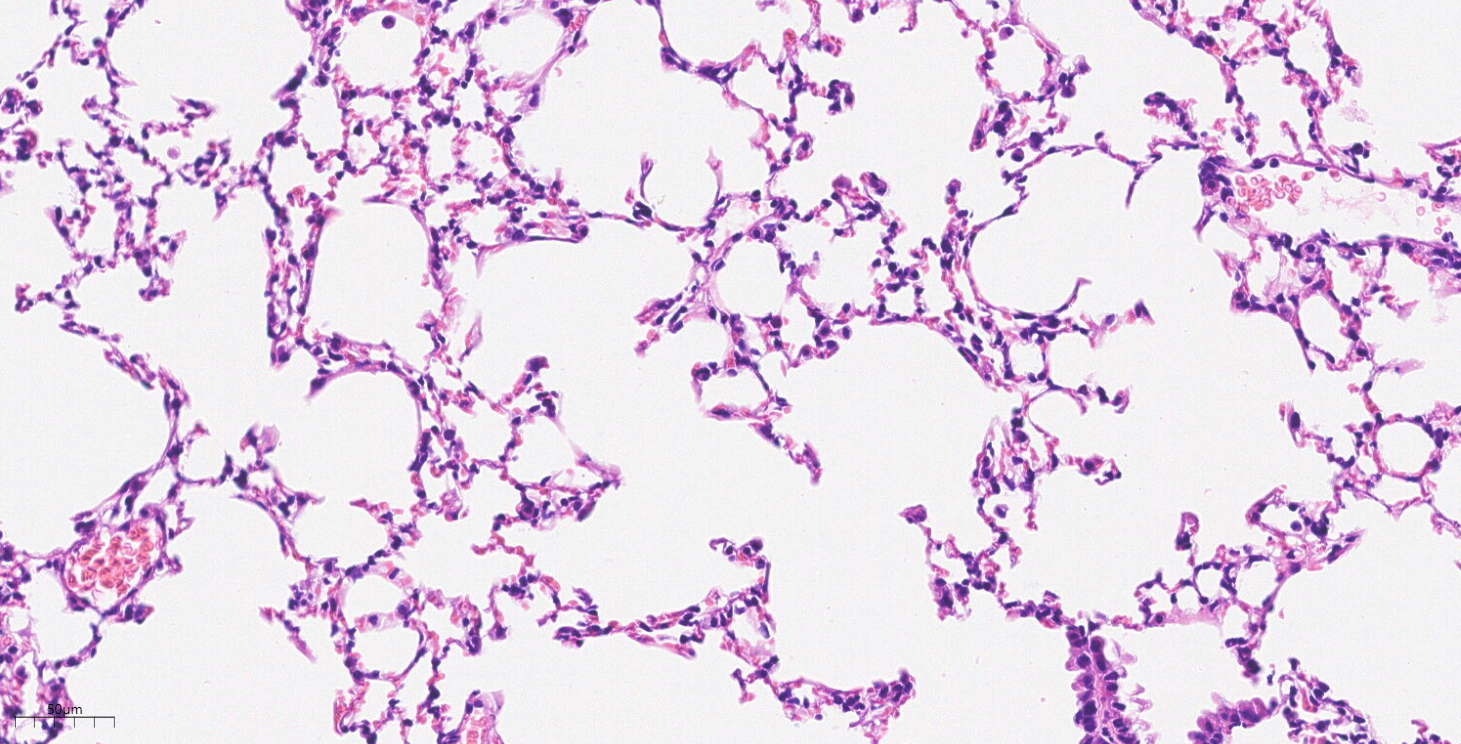

Supplement: Supplementary file 3 — EV Figures Source Data [file 44321_2025_220_MOESM3_ESM.zip › EMM-2024-20638-V3_EV Figures/Figure EV3/Figure EV3C/KO sham/KO sham.jpg]

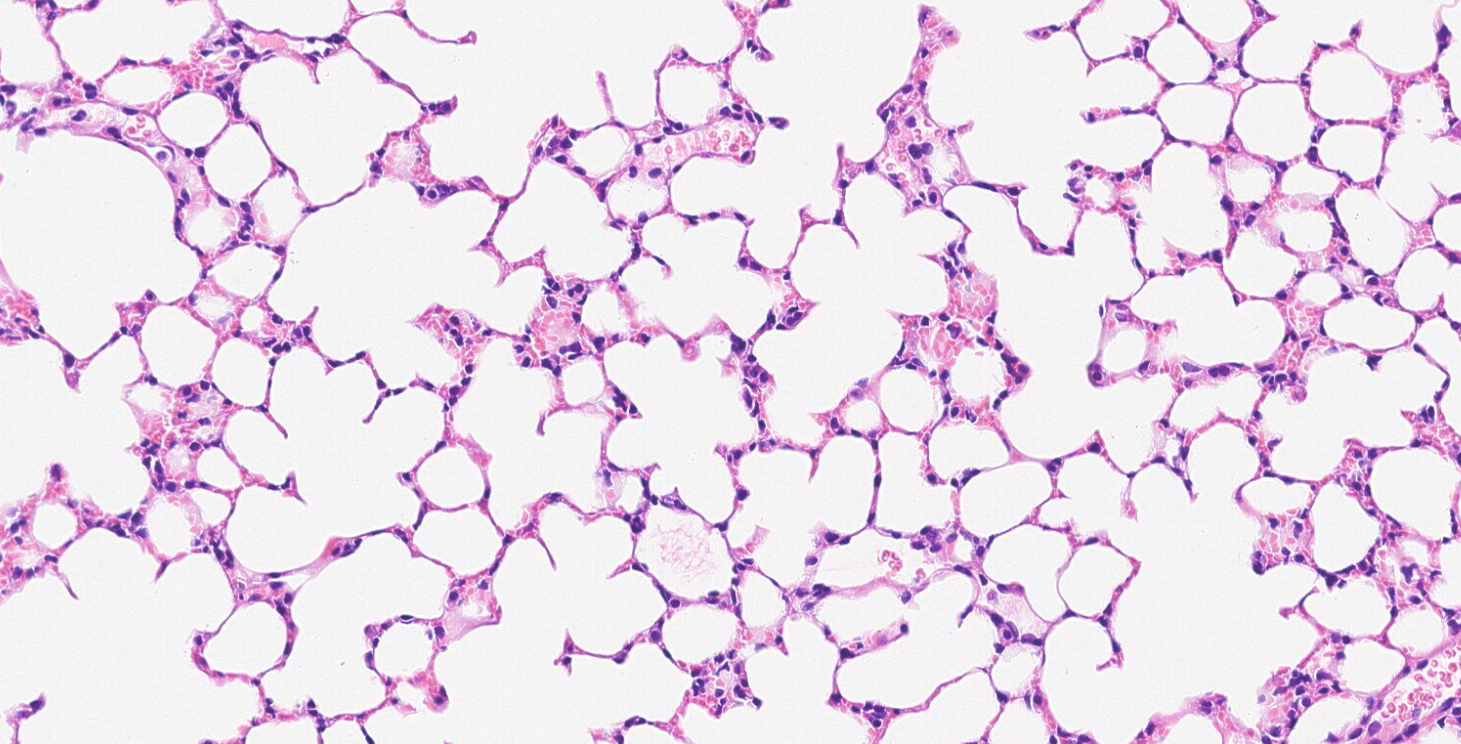

Supplement: Supplementary file 3 — EV Figures Source Data [file 44321_2025_220_MOESM3_ESM.zip › EMM-2024-20638-V3_EV Figures/Figure EV3/Figure EV3C/WT sham/WT sham.jpg]

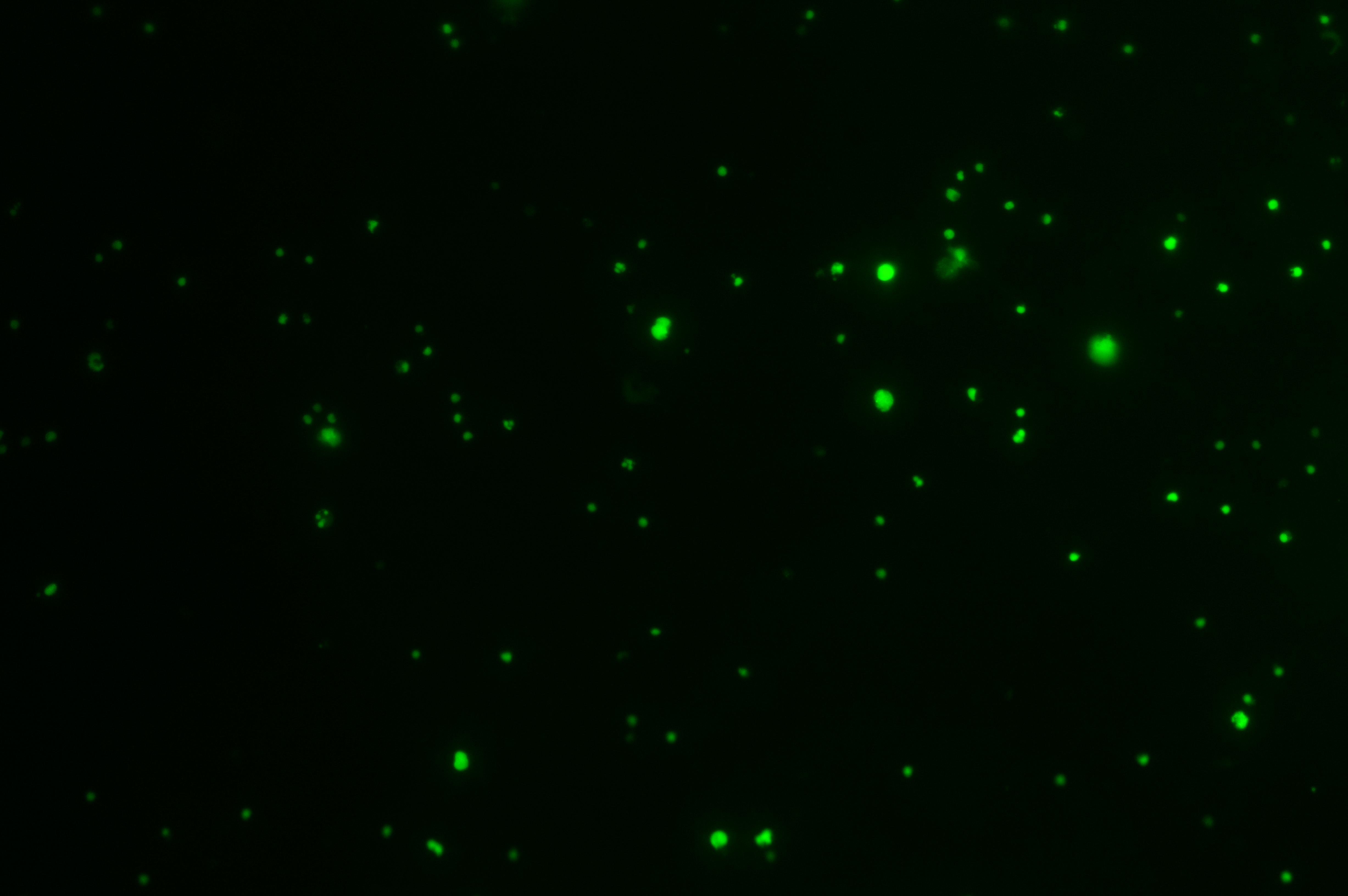

Supplement: Supplementary file 3 — EV Figures Source Data [file 44321_2025_220_MOESM3_ESM.zip › EMM-2024-20638-V3_EV Figures/Figure EV4/Figure EV4E/control/control.tif]

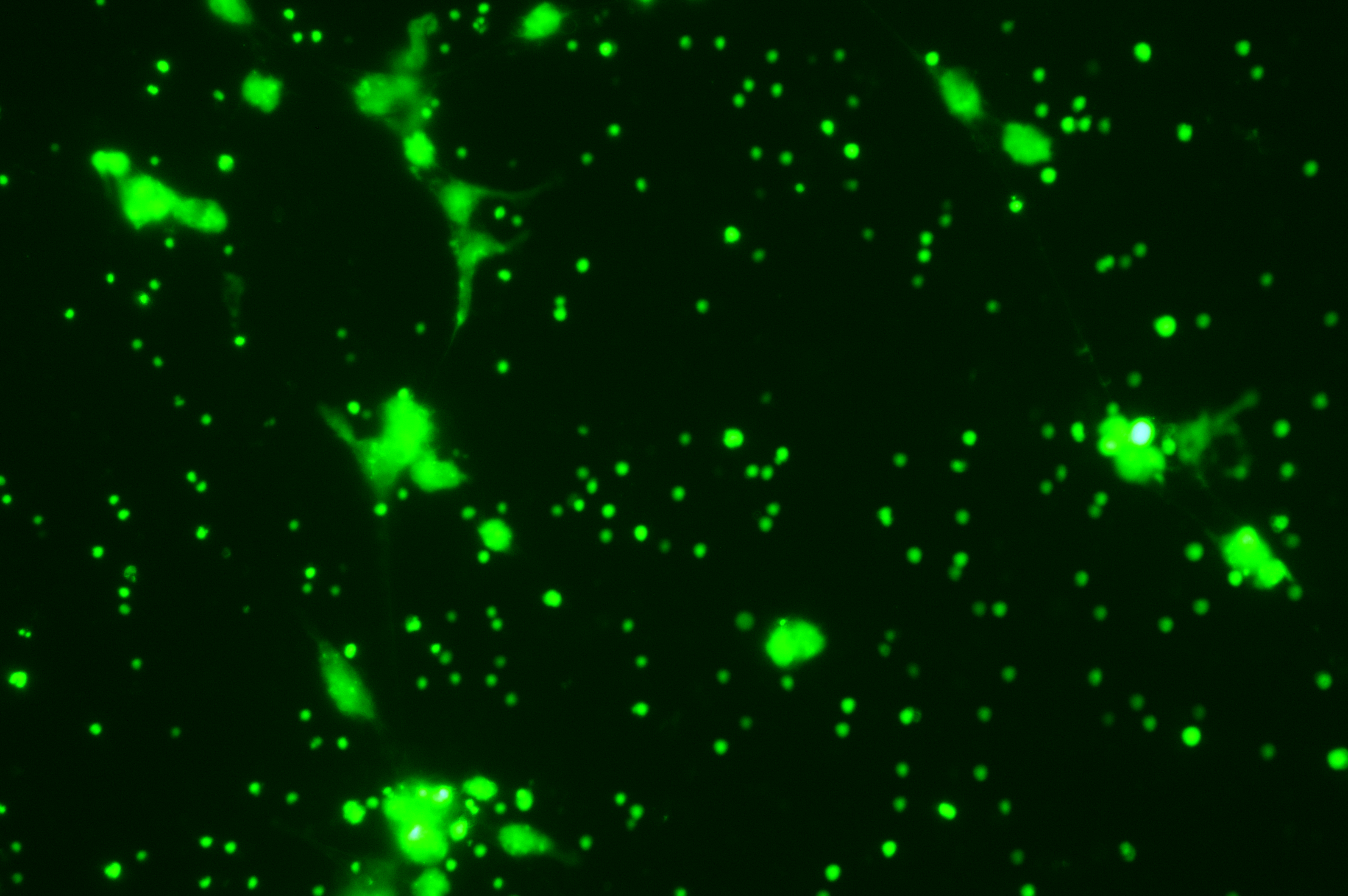

Supplement: Supplementary file 3 — EV Figures Source Data [file 44321_2025_220_MOESM3_ESM.zip › EMM-2024-20638-V3_EV Figures/Figure EV4/Figure EV4E/LPS/LPS.tif]

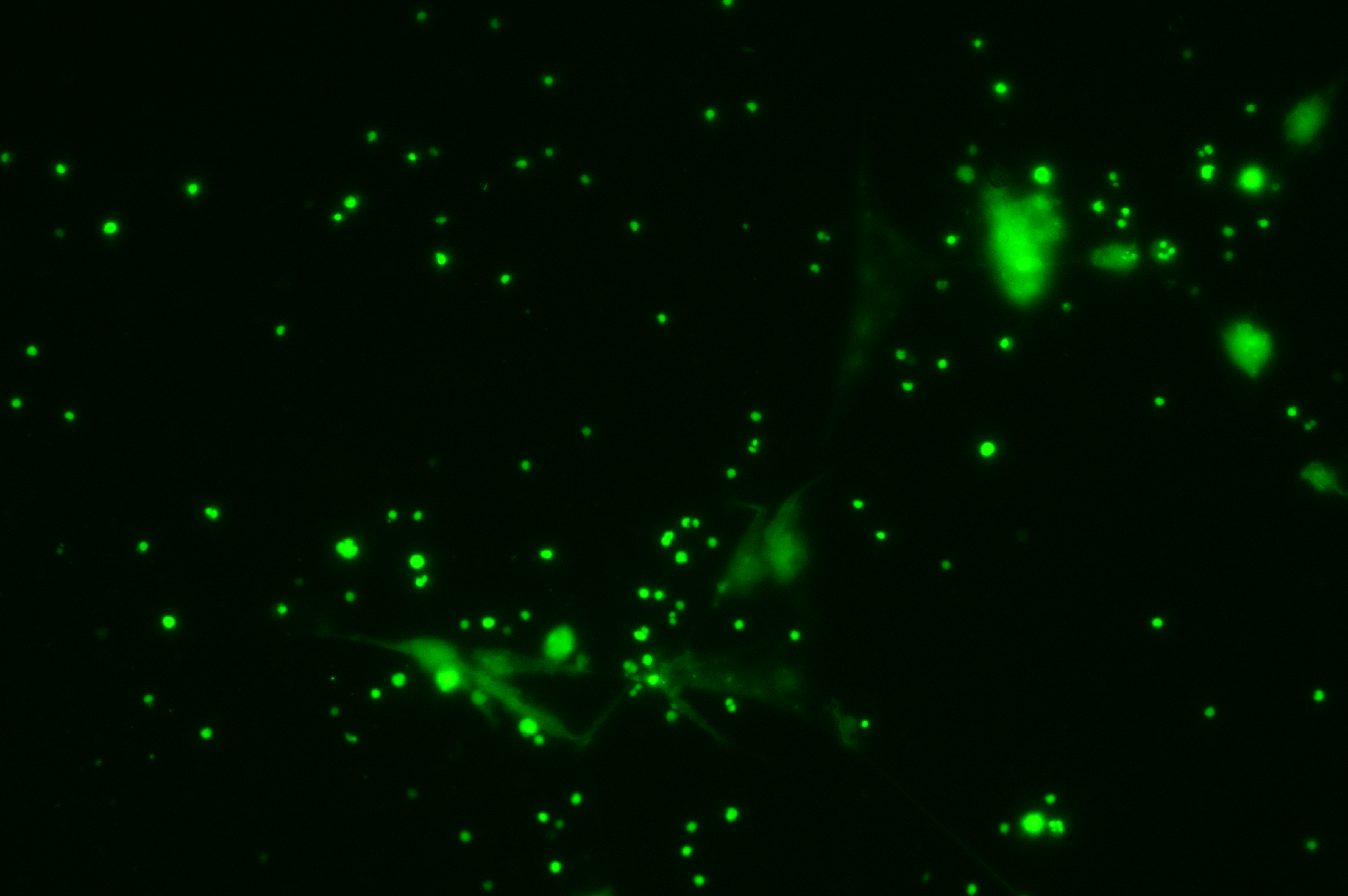

Supplement: Supplementary file 3 — EV Figures Source Data [file 44321_2025_220_MOESM3_ESM.zip › EMM-2024-20638-V3_EV Figures/Figure EV4/Figure EV4E/LPS+i-HPK1/LPS+i-HPK1.tif]

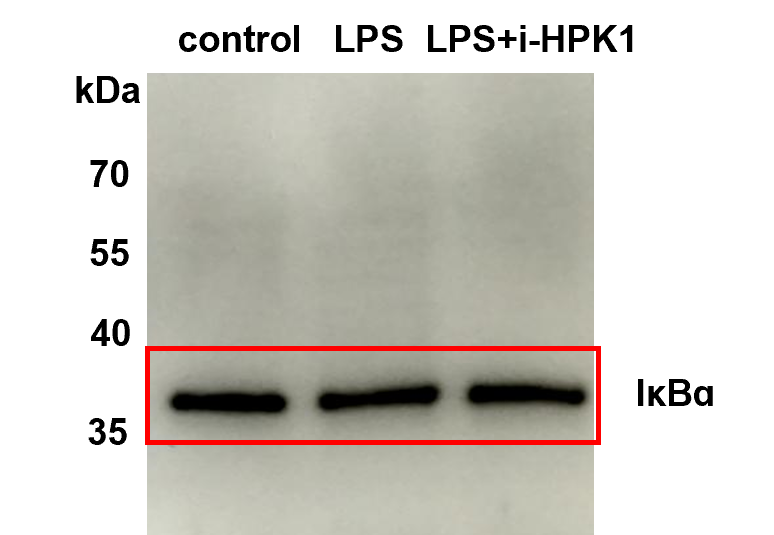

Supplement: Supplementary file 3 — EV Figures Source Data [file 44321_2025_220_MOESM3_ESM.zip › EMM-2024-20638-V3_EV Figures/Figure EV4/Figure EV4G/IκBɑ.tif]

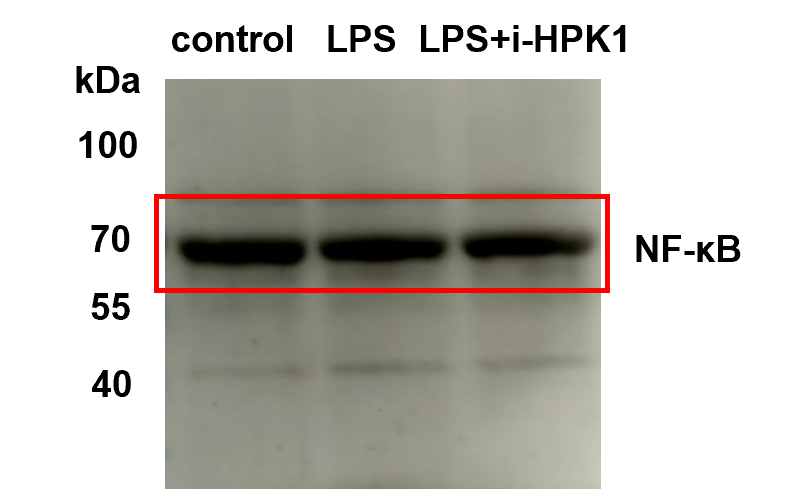

Supplement: Supplementary file 3 — EV Figures Source Data [file 44321_2025_220_MOESM3_ESM.zip › EMM-2024-20638-V3_EV Figures/Figure EV4/Figure EV4G/NF-κB.tif]

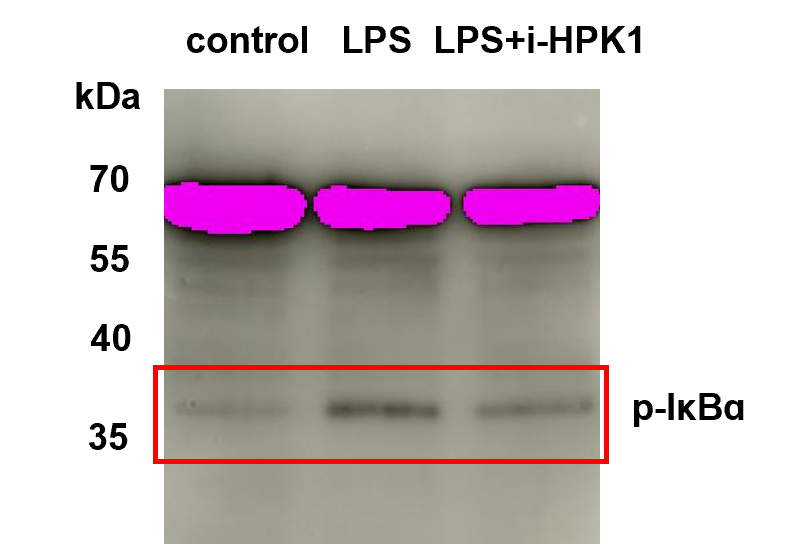

Supplement: Supplementary file 3 — EV Figures Source Data [file 44321_2025_220_MOESM3_ESM.zip › EMM-2024-20638-V3_EV Figures/Figure EV4/Figure EV4G/p-IκBɑ.tif]

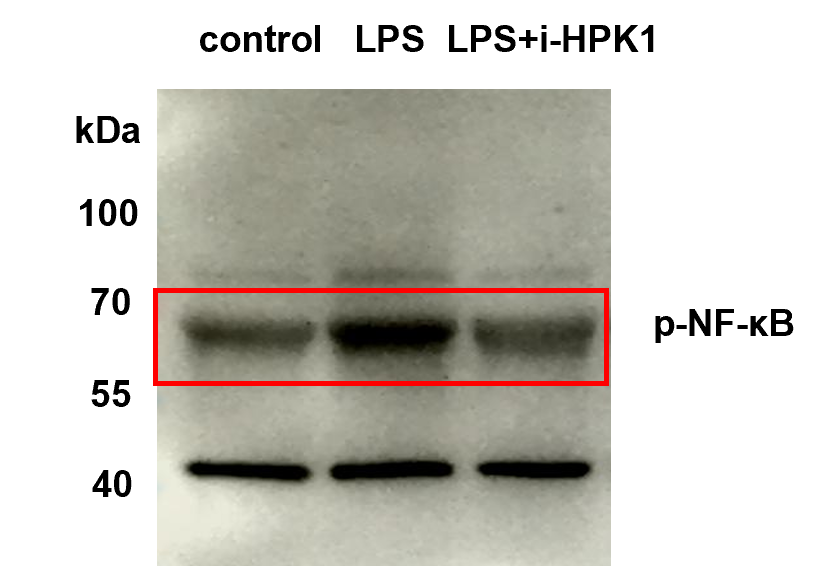

Supplement: Supplementary file 3 — EV Figures Source Data [file 44321_2025_220_MOESM3_ESM.zip › EMM-2024-20638-V3_EV Figures/Figure EV4/Figure EV4G/p-NF-κB.tif]

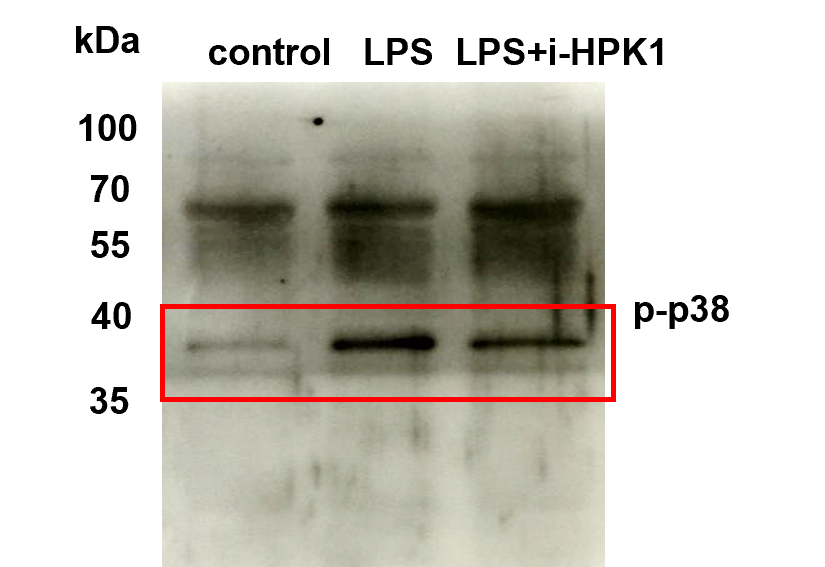

Supplement: Supplementary file 3 — EV Figures Source Data [file 44321_2025_220_MOESM3_ESM.zip › EMM-2024-20638-V3_EV Figures/Figure EV4/Figure EV4G/p-p38.tif]

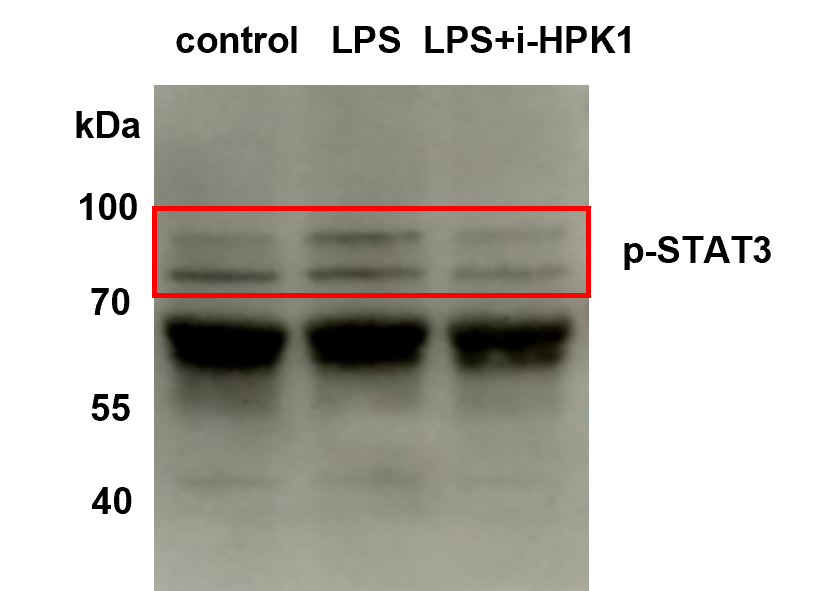

Supplement: Supplementary file 3 — EV Figures Source Data [file 44321_2025_220_MOESM3_ESM.zip › EMM-2024-20638-V3_EV Figures/Figure EV4/Figure EV4G/p-STAT3.tif]

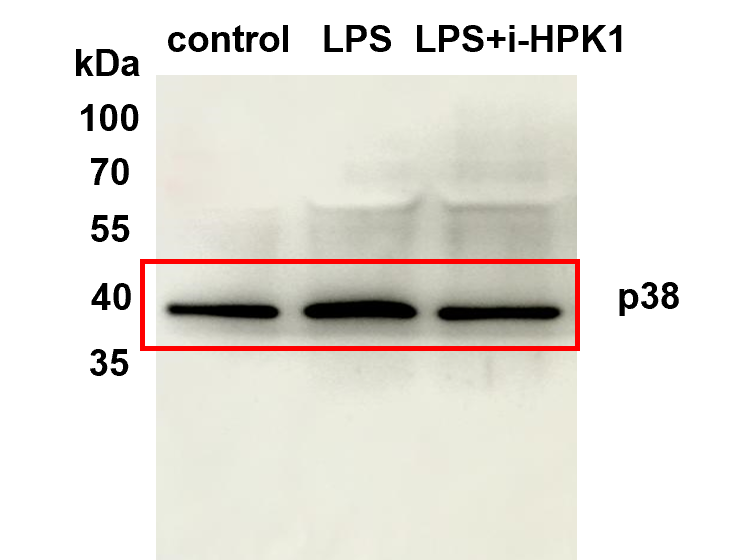

Supplement: Supplementary file 3 — EV Figures Source Data [file 44321_2025_220_MOESM3_ESM.zip › EMM-2024-20638-V3_EV Figures/Figure EV4/Figure EV4G/p38.tif]

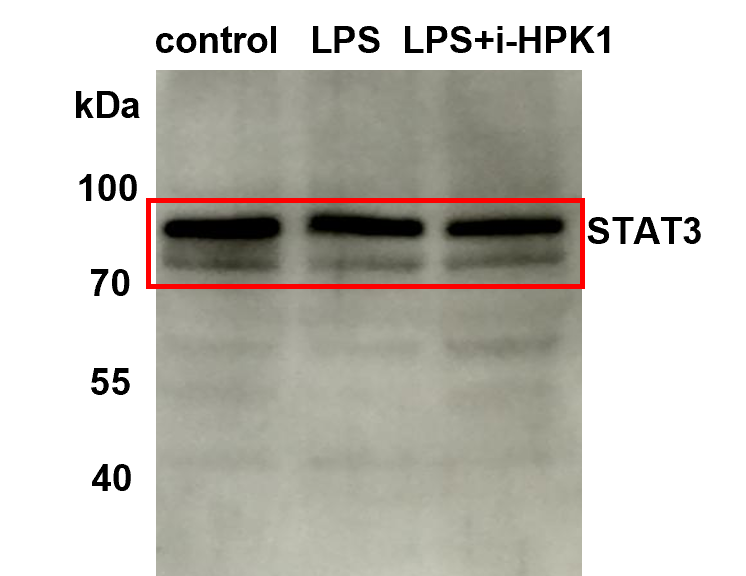

Supplement: Supplementary file 3 — EV Figures Source Data [file 44321_2025_220_MOESM3_ESM.zip › EMM-2024-20638-V3_EV Figures/Figure EV4/Figure EV4G/STAT3.tif]

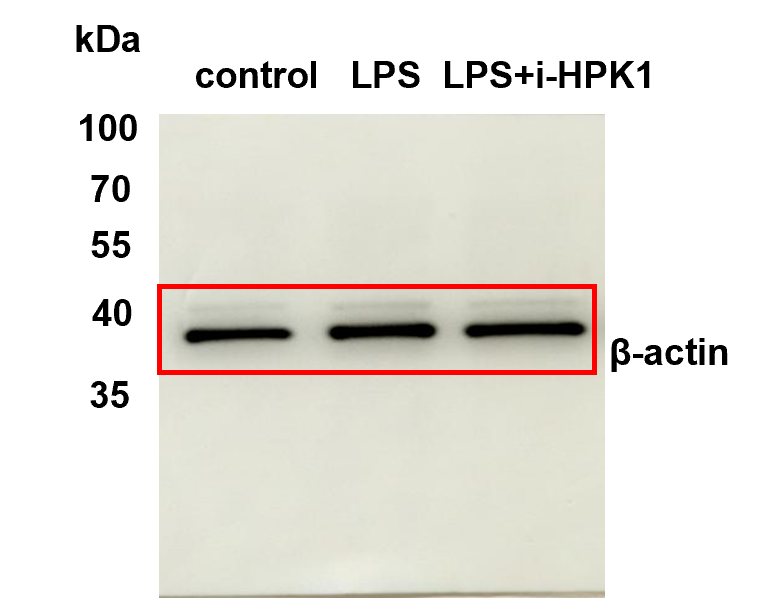

Supplement: Supplementary file 3 — EV Figures Source Data [file 44321_2025_220_MOESM3_ESM.zip › EMM-2024-20638-V3_EV Figures/Figure EV4/Figure EV4G/β-actin.tif]

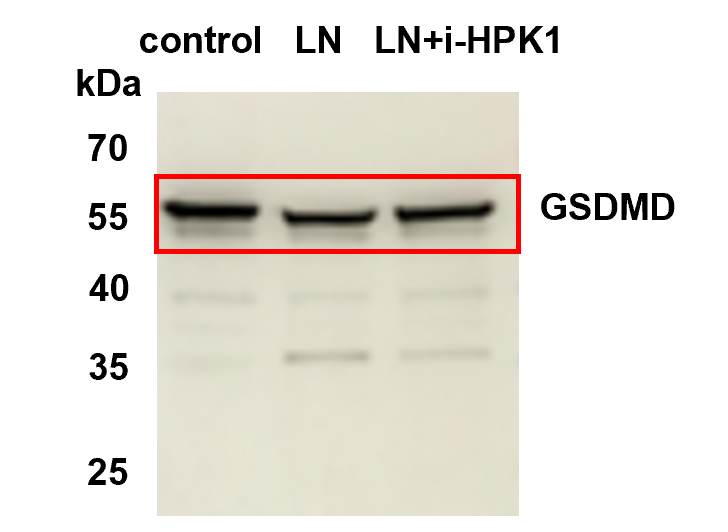

Supplement: Supplementary file 3 — EV Figures Source Data [file 44321_2025_220_MOESM3_ESM.zip › EMM-2024-20638-V3_EV Figures/Figure EV4/Figure EV4L/GSDMD.tif]

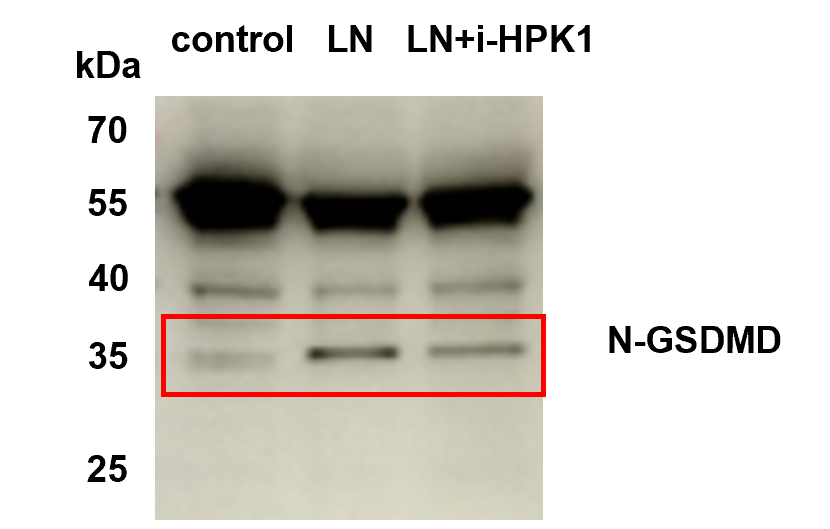

Supplement: Supplementary file 3 — EV Figures Source Data [file 44321_2025_220_MOESM3_ESM.zip › EMM-2024-20638-V3_EV Figures/Figure EV4/Figure EV4L/N-GSDMD.tif]

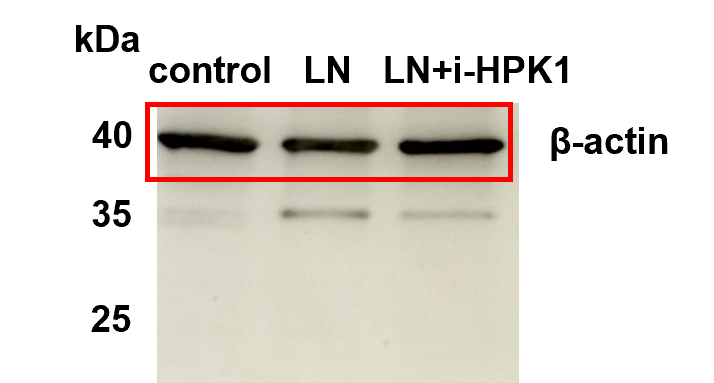

Supplement: Supplementary file 3 — EV Figures Source Data [file 44321_2025_220_MOESM3_ESM.zip › EMM-2024-20638-V3_EV Figures/Figure EV4/Figure EV4L/β-actin.tif]

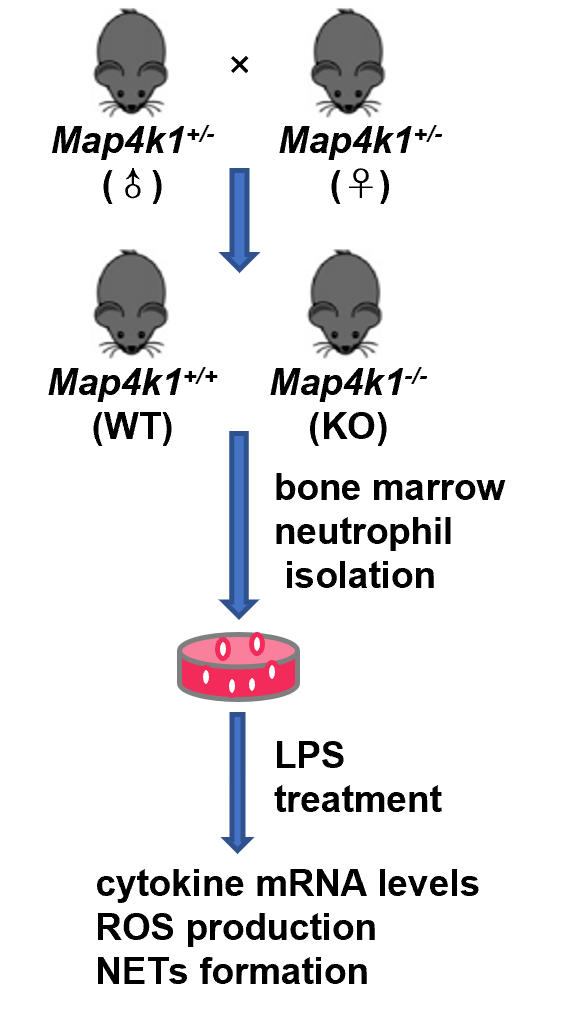

Supplement: Supplementary file 4 — Source data Fig. 1 [file 44321_2025_220_MOESM4_ESM.zip › EMM-2024-20638-V3_Figure 1/Figure 1A/Figure 1A.tif]

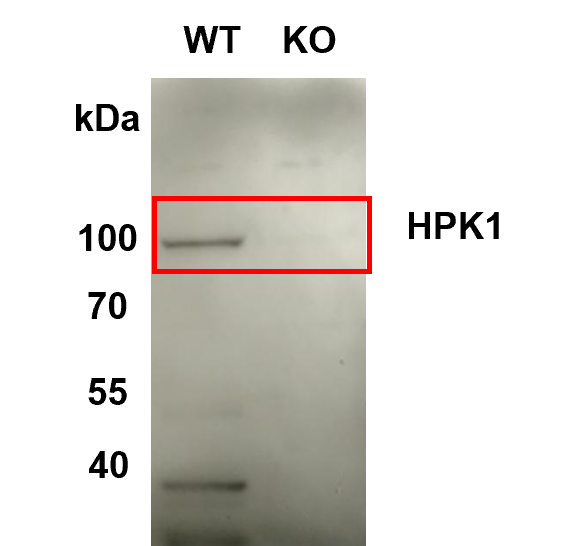

Supplement: Supplementary file 4 — Source data Fig. 1 [file 44321_2025_220_MOESM4_ESM.zip › EMM-2024-20638-V3_Figure 1/Figure 1C/HPK1.tif]

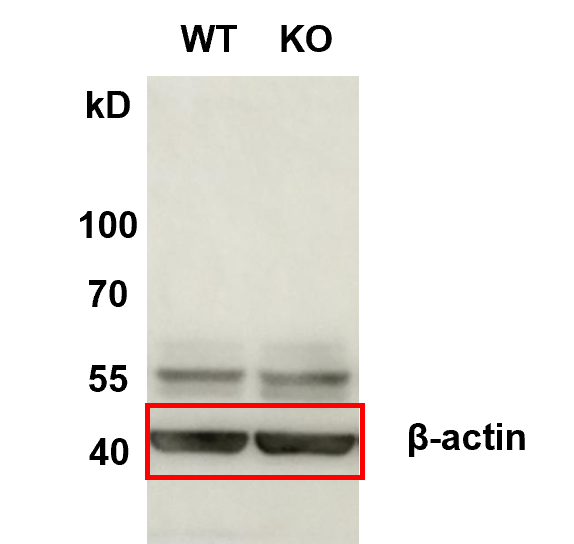

Supplement: Supplementary file 4 — Source data Fig. 1 [file 44321_2025_220_MOESM4_ESM.zip › EMM-2024-20638-V3_Figure 1/Figure 1C/β-actin.tif]

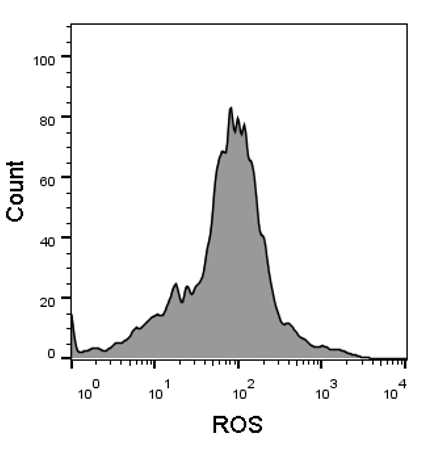

Supplement: Supplementary file 4 — Source data Fig. 1 [file 44321_2025_220_MOESM4_ESM.zip › EMM-2024-20638-V3_Figure 1/Figure 1I/KO control.tif]

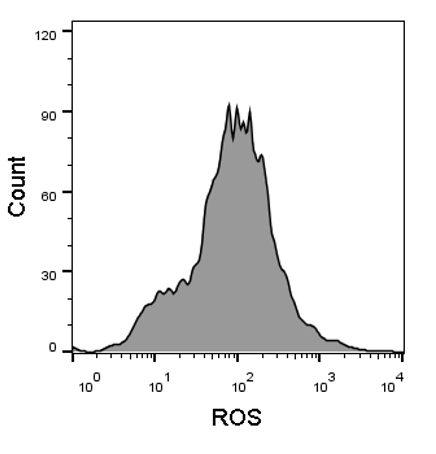

Supplement: Supplementary file 4 — Source data Fig. 1 [file 44321_2025_220_MOESM4_ESM.zip › EMM-2024-20638-V3_Figure 1/Figure 1I/KO LPS.tif]

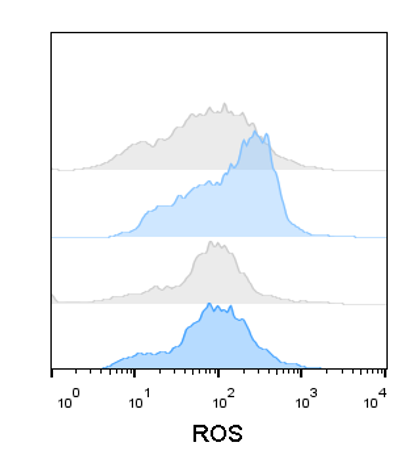

Supplement: Supplementary file 4 — Source data Fig. 1 [file 44321_2025_220_MOESM4_ESM.zip › EMM-2024-20638-V3_Figure 1/Figure 1I/offset.tif]

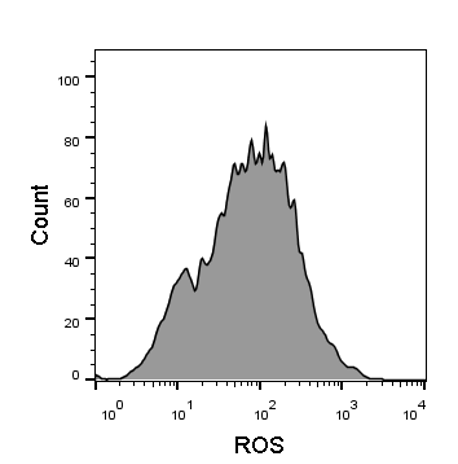

Supplement: Supplementary file 4 — Source data Fig. 1 [file 44321_2025_220_MOESM4_ESM.zip › EMM-2024-20638-V3_Figure 1/Figure 1I/WT control.tif]

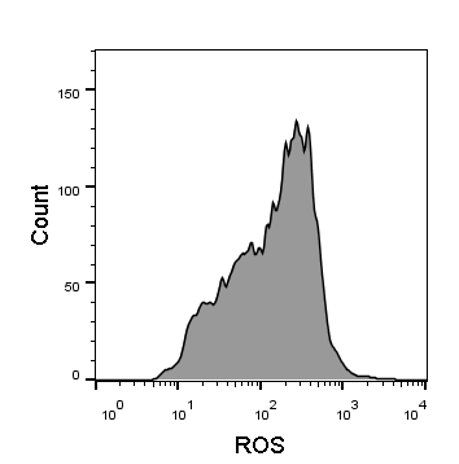

Supplement: Supplementary file 4 — Source data Fig. 1 [file 44321_2025_220_MOESM4_ESM.zip › EMM-2024-20638-V3_Figure 1/Figure 1I/WT LPS.tif]

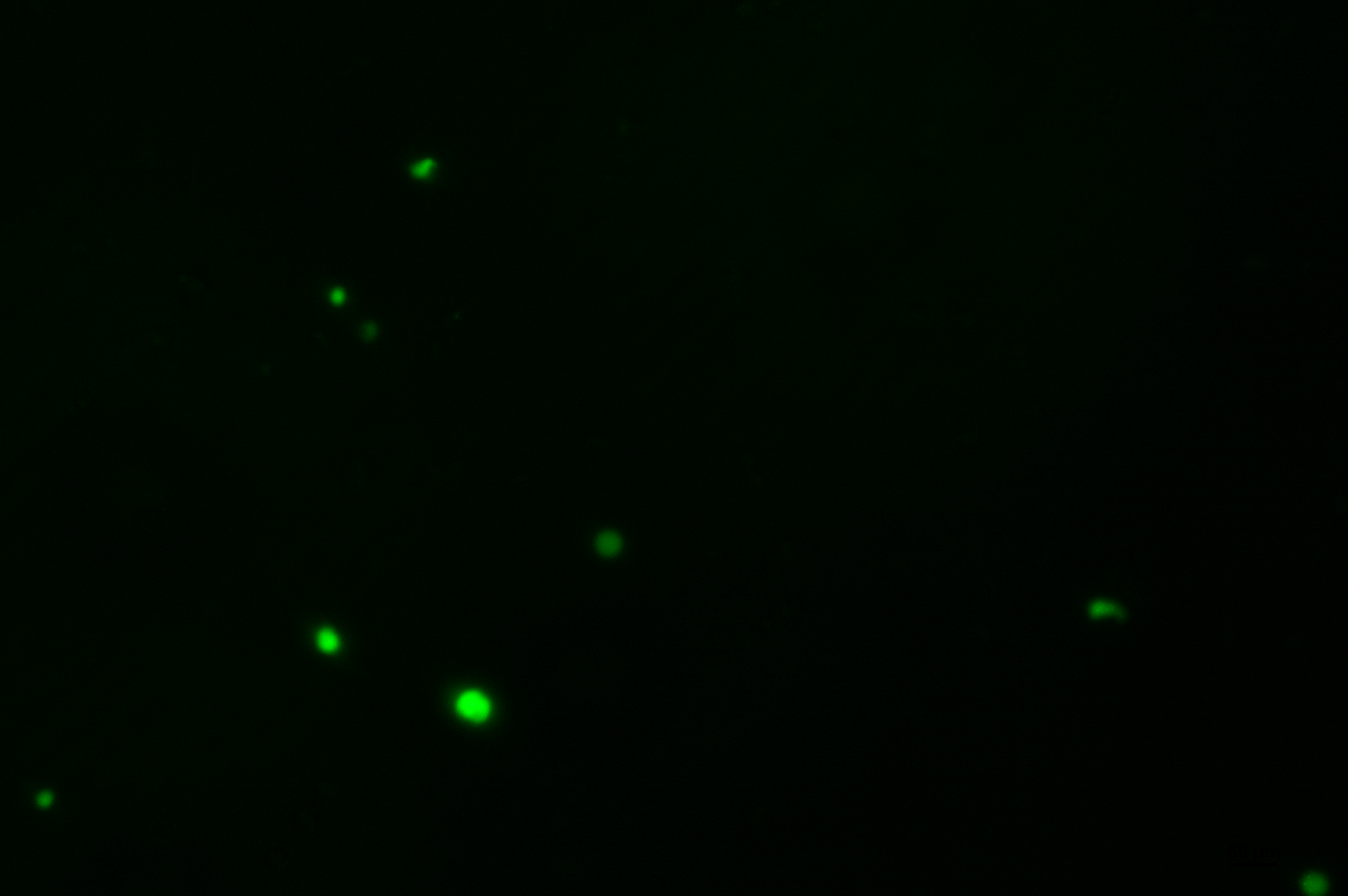

Supplement: Supplementary file 4 — Source data Fig. 1 [file 44321_2025_220_MOESM4_ESM.zip › EMM-2024-20638-V3_Figure 1/Figure 1K/KO control/sytox green.tif]

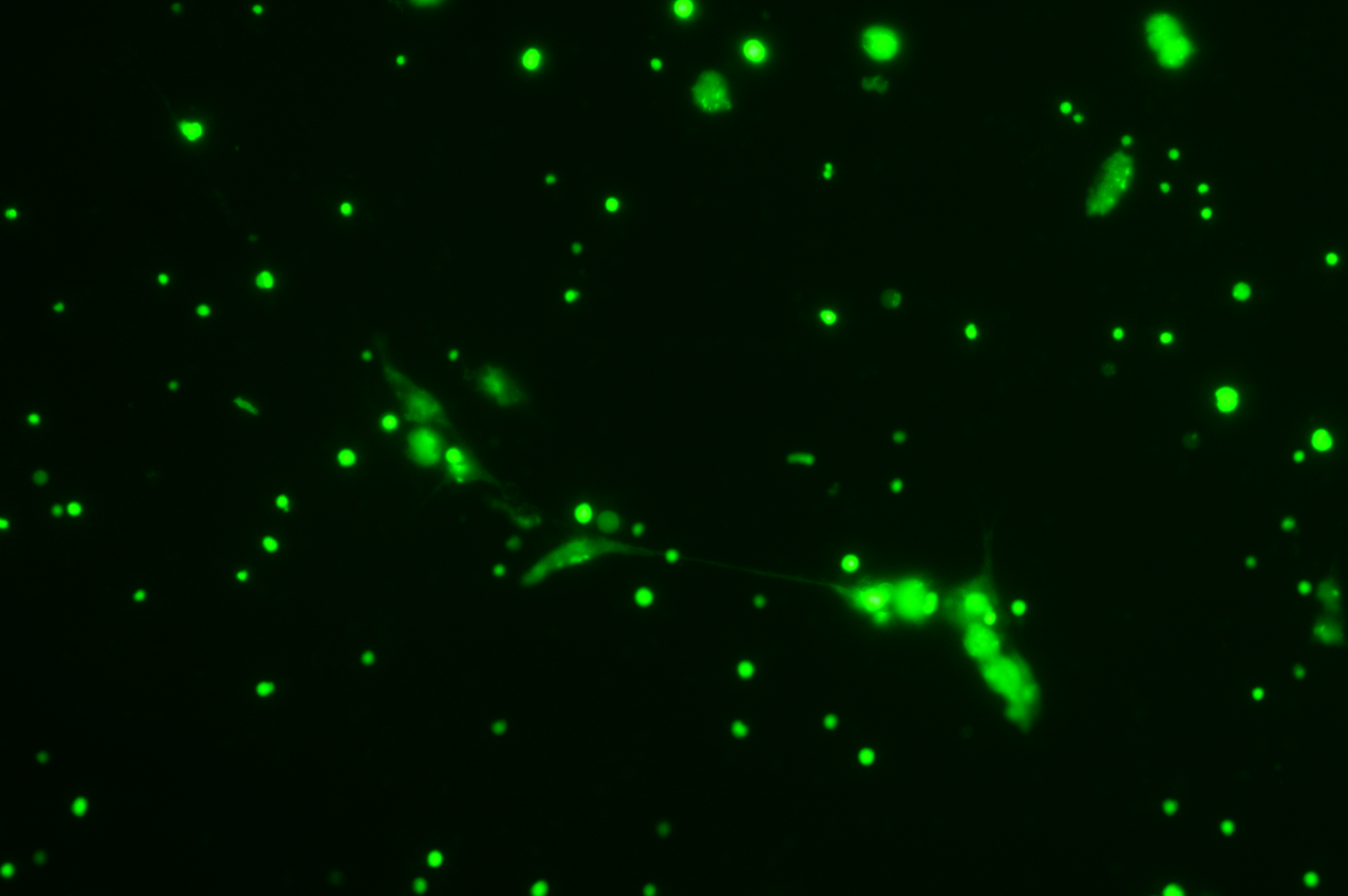

Supplement: Supplementary file 4 — Source data Fig. 1 [file 44321_2025_220_MOESM4_ESM.zip › EMM-2024-20638-V3_Figure 1/Figure 1K/KO LPS/sytox green.tif]

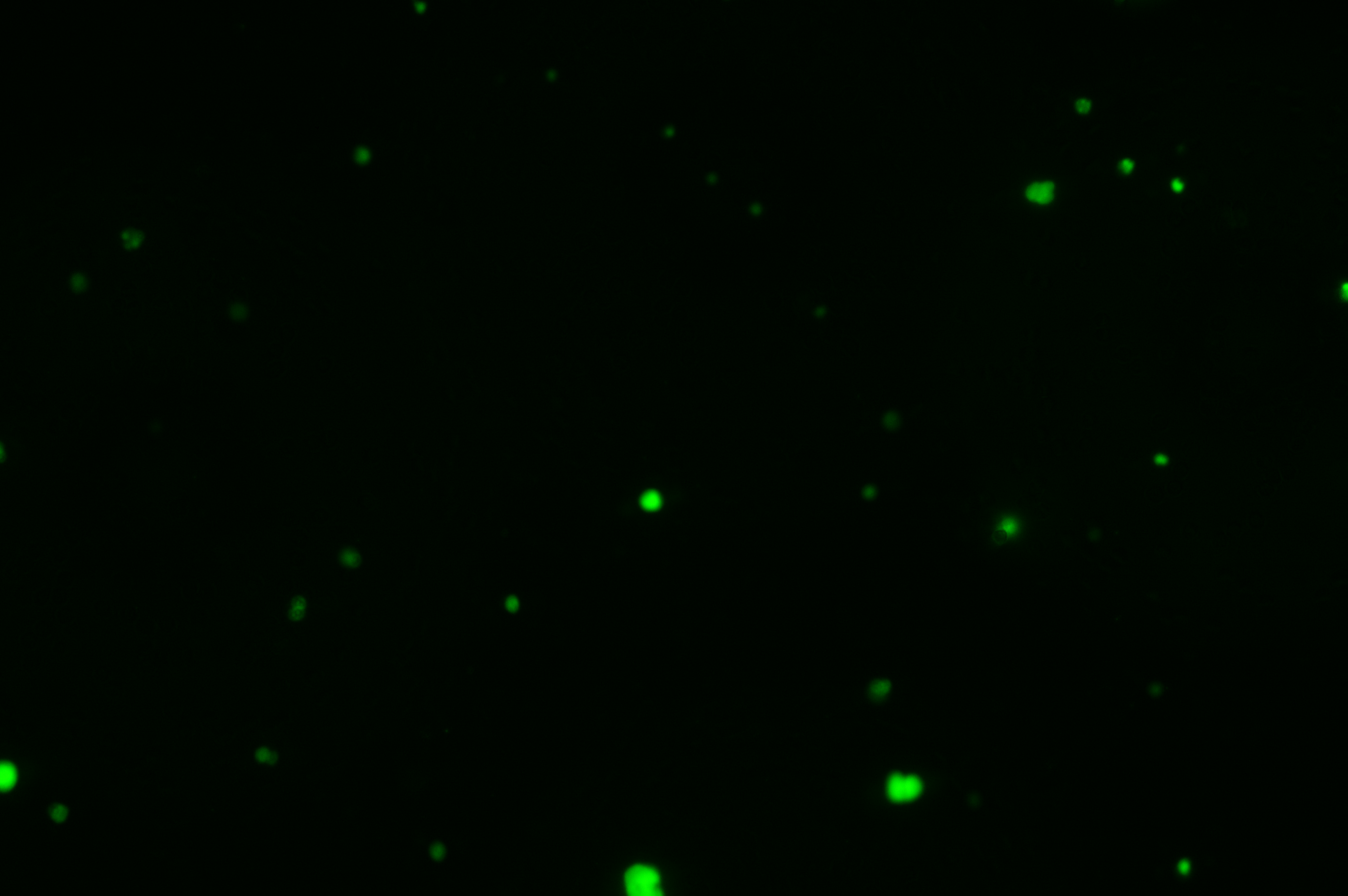

Supplement: Supplementary file 4 — Source data Fig. 1 [file 44321_2025_220_MOESM4_ESM.zip › EMM-2024-20638-V3_Figure 1/Figure 1K/WT control/sytox green.tif]

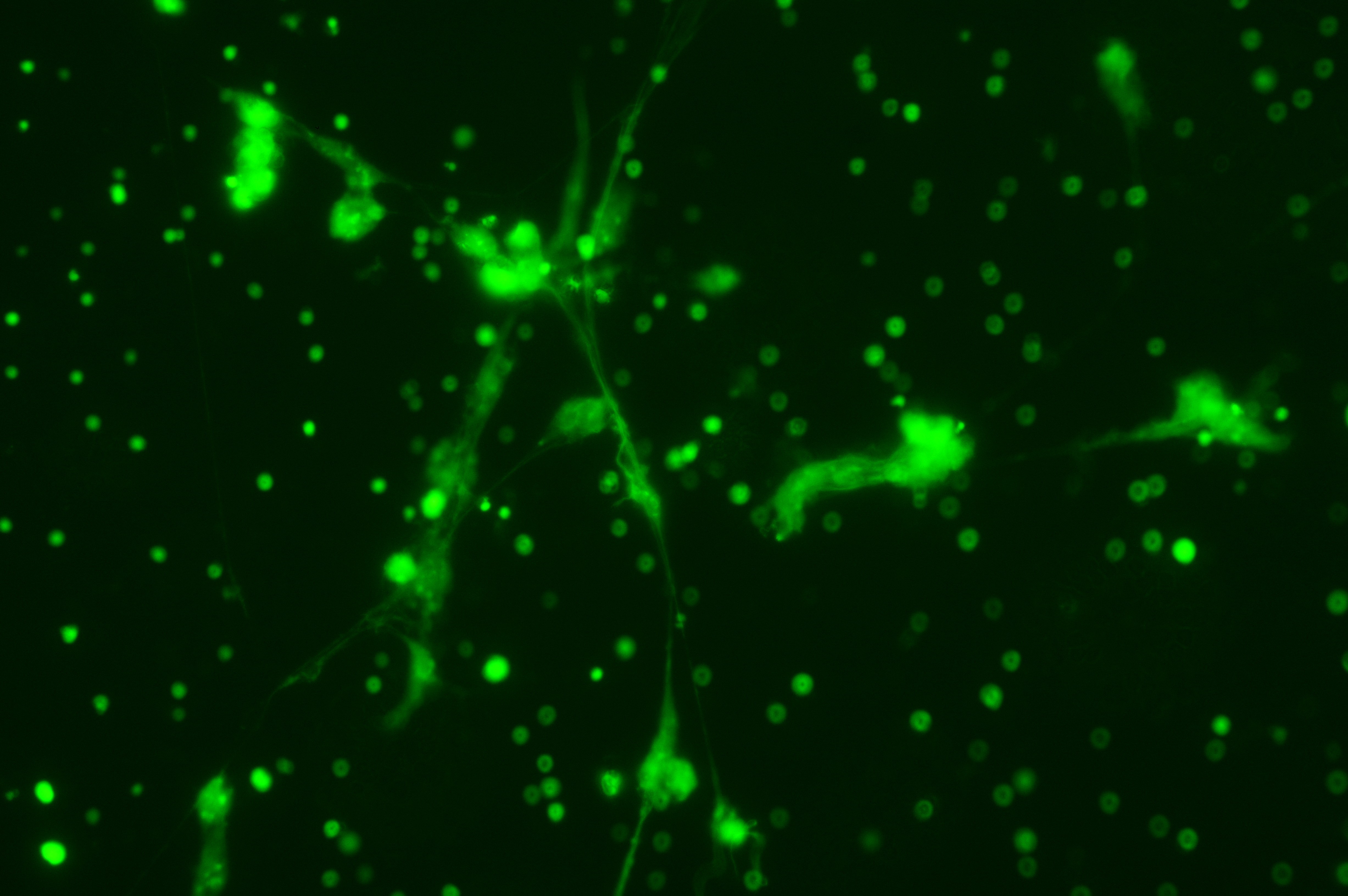

Supplement: Supplementary file 4 — Source data Fig. 1 [file 44321_2025_220_MOESM4_ESM.zip › EMM-2024-20638-V3_Figure 1/Figure 1K/WT LPS/sytox green.tif]

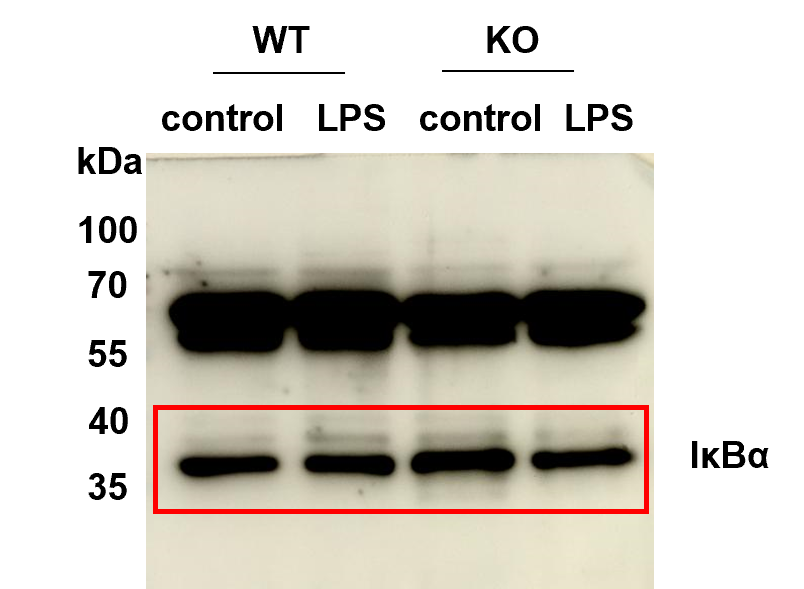

Supplement: Supplementary file 5 — Source data Fig. 2 [file 44321_2025_220_MOESM5_ESM.zip › Figure 2/Figure 2A/IκBα.tif]

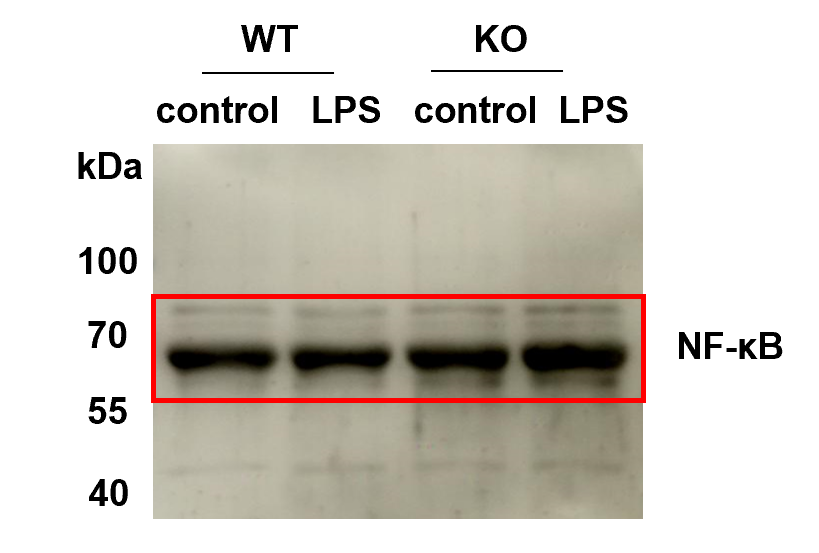

Supplement: Supplementary file 5 — Source data Fig. 2 [file 44321_2025_220_MOESM5_ESM.zip › Figure 2/Figure 2A/NF-κB.tif]

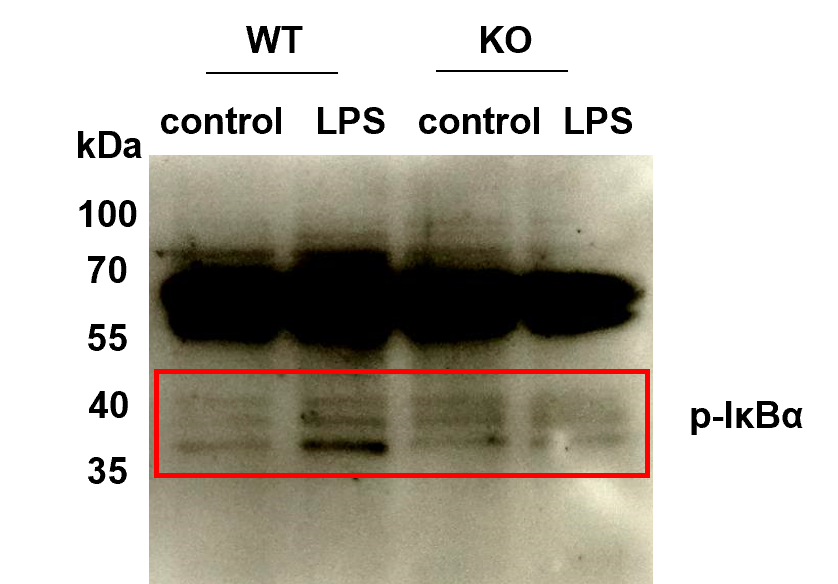

Supplement: Supplementary file 5 — Source data Fig. 2 [file 44321_2025_220_MOESM5_ESM.zip › Figure 2/Figure 2A/p-IκBα.tif]

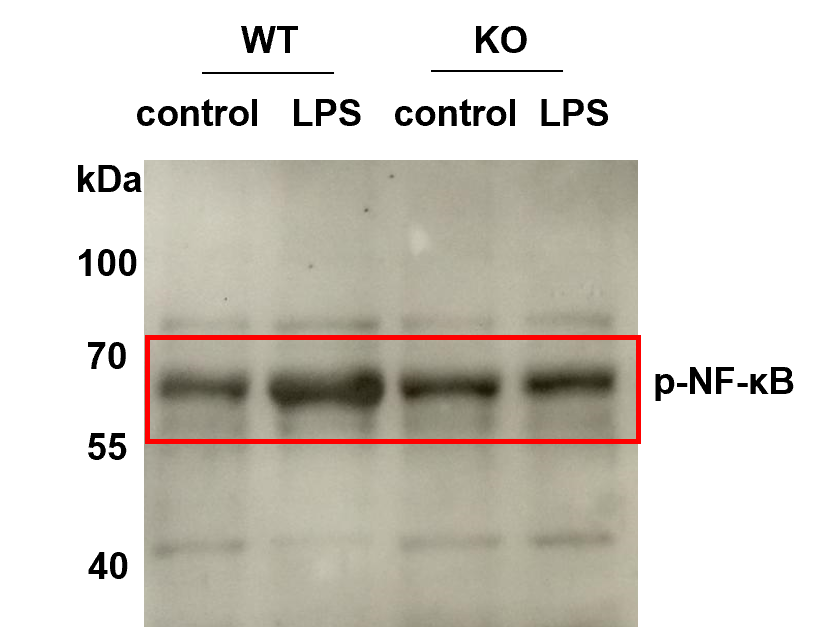

Supplement: Supplementary file 5 — Source data Fig. 2 [file 44321_2025_220_MOESM5_ESM.zip › Figure 2/Figure 2A/p-NF-κB.tif]

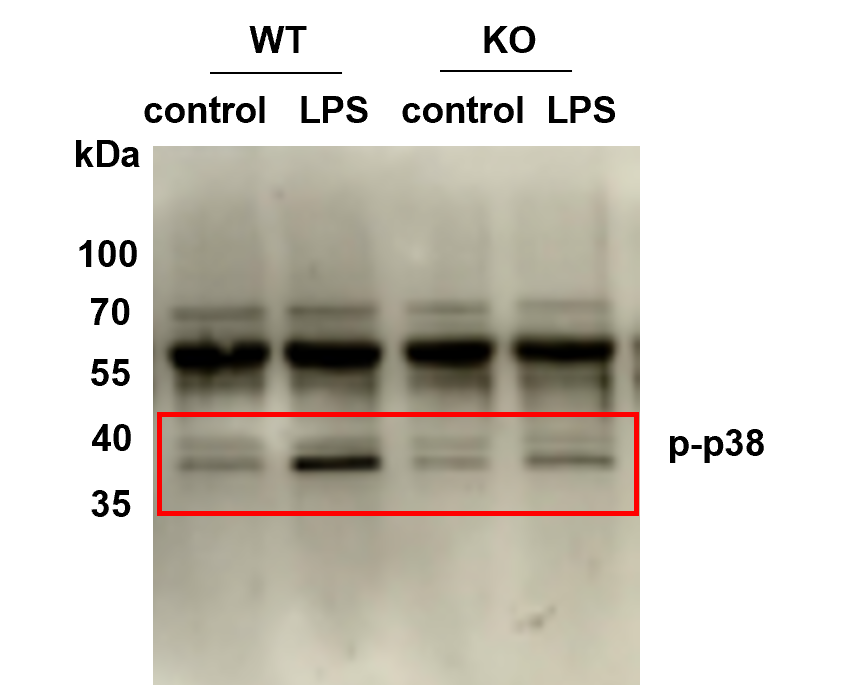

Supplement: Supplementary file 5 — Source data Fig. 2 [file 44321_2025_220_MOESM5_ESM.zip › Figure 2/Figure 2A/p-p38.tif]

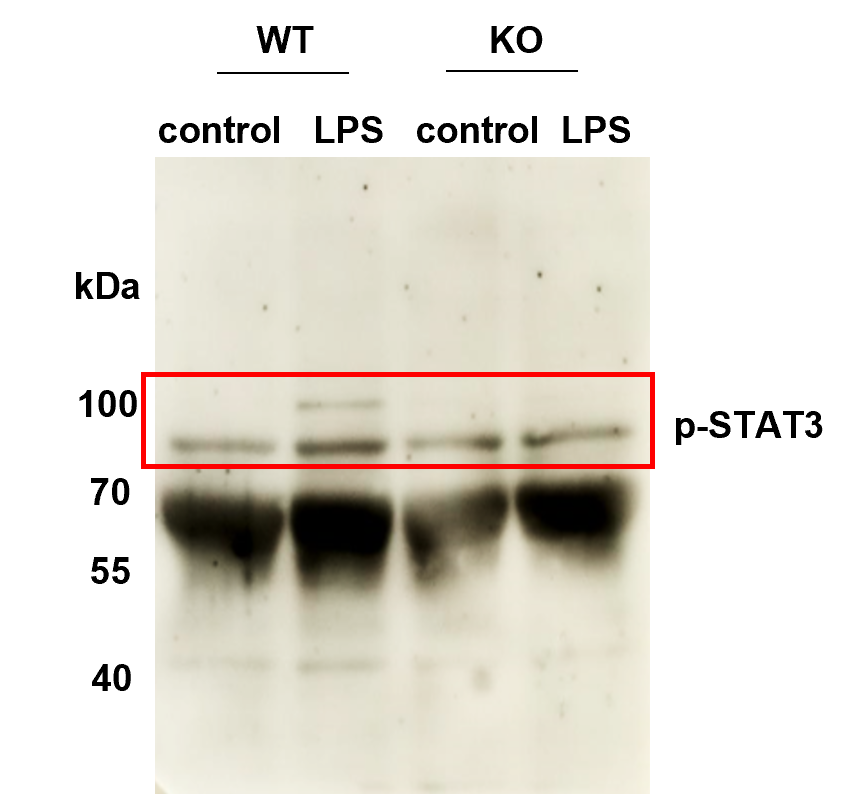

Supplement: Supplementary file 5 — Source data Fig. 2 [file 44321_2025_220_MOESM5_ESM.zip › Figure 2/Figure 2A/p-STAT3.tif]

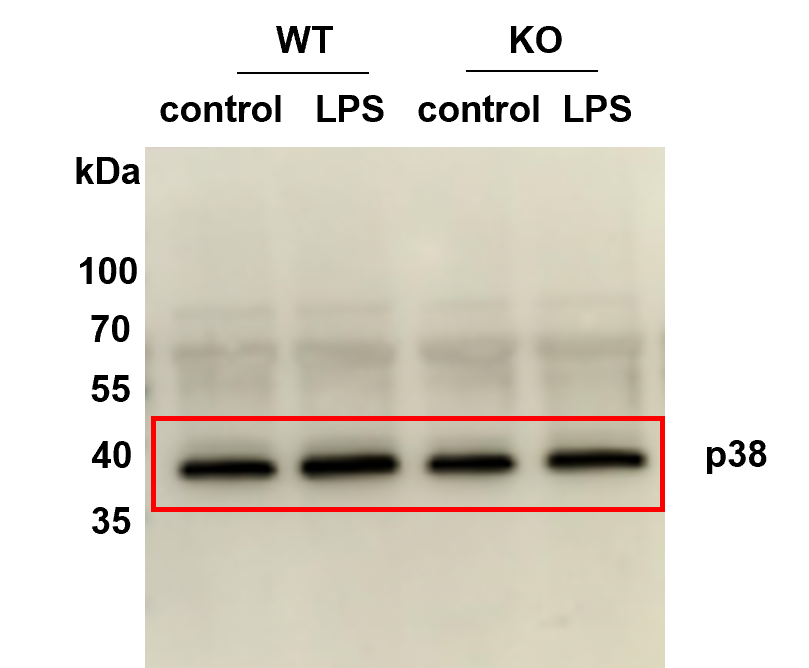

Supplement: Supplementary file 5 — Source data Fig. 2 [file 44321_2025_220_MOESM5_ESM.zip › Figure 2/Figure 2A/p38.tif]

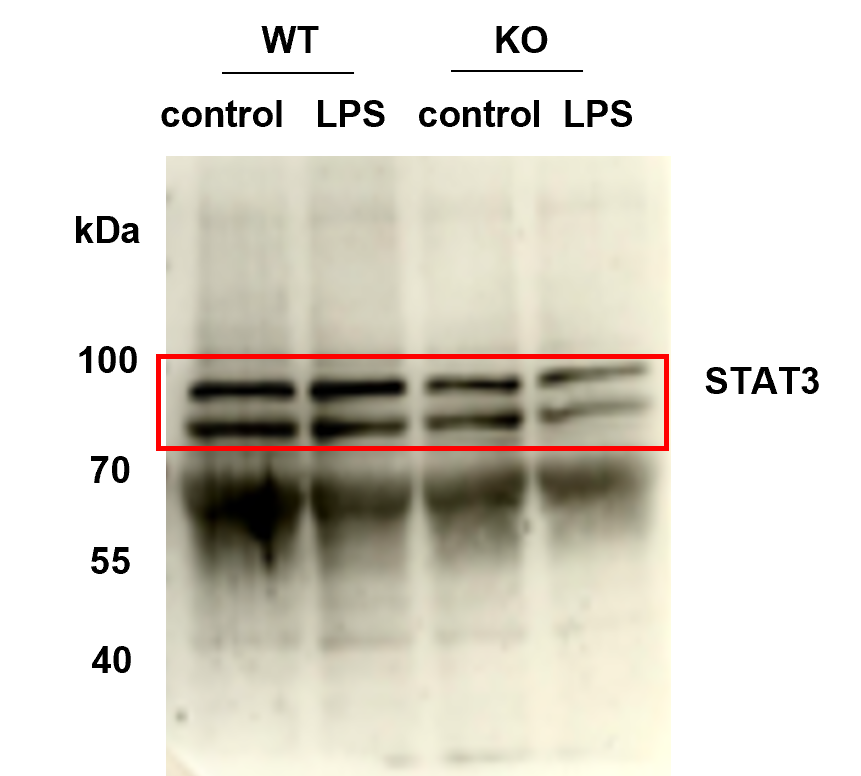

Supplement: Supplementary file 5 — Source data Fig. 2 [file 44321_2025_220_MOESM5_ESM.zip › Figure 2/Figure 2A/STAT3.tif]

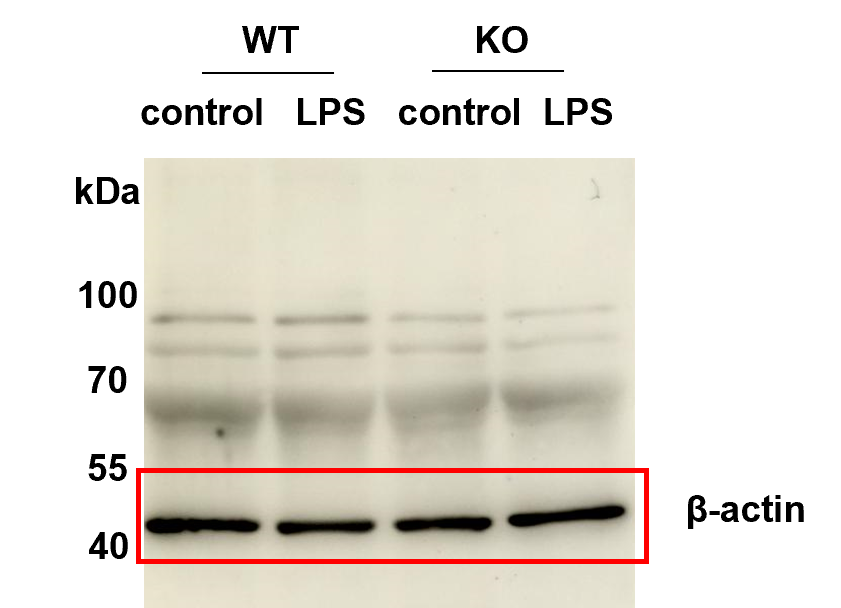

Supplement: Supplementary file 5 — Source data Fig. 2 [file 44321_2025_220_MOESM5_ESM.zip › Figure 2/Figure 2A/β-actin.tif]

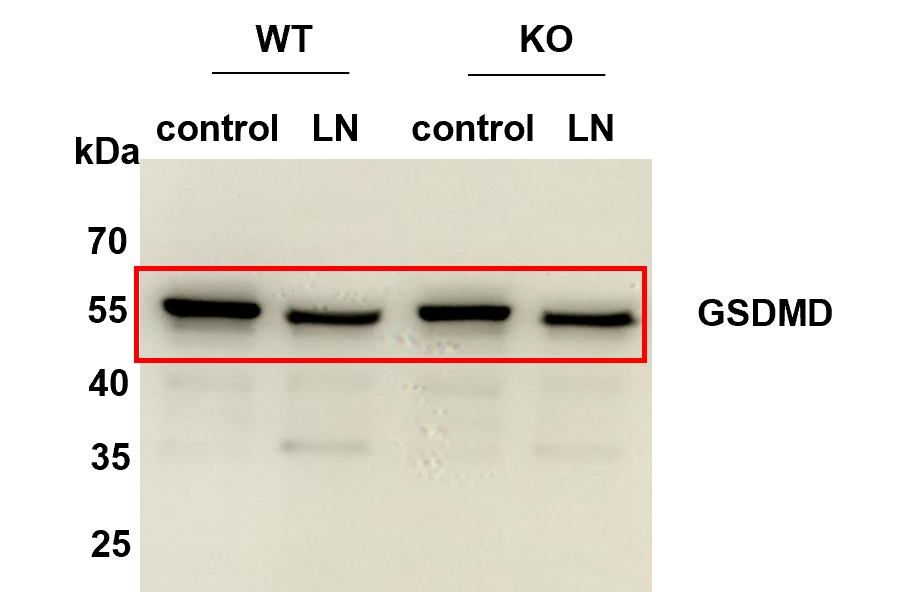

Supplement: Supplementary file 5 — Source data Fig. 2 [file 44321_2025_220_MOESM5_ESM.zip › Figure 2/Figure 2F/GSDMD.tif]

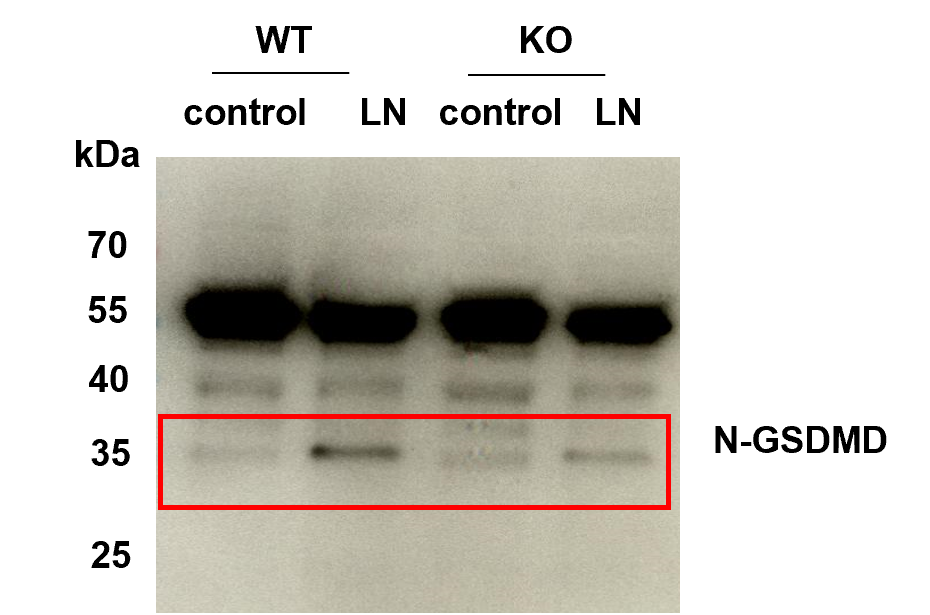

Supplement: Supplementary file 5 — Source data Fig. 2 [file 44321_2025_220_MOESM5_ESM.zip › Figure 2/Figure 2F/N-GSDMD.tif]

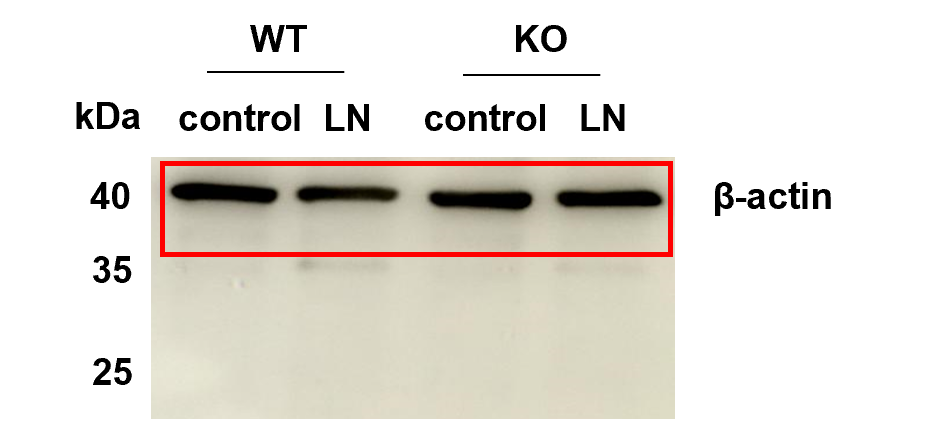

Supplement: Supplementary file 5 — Source data Fig. 2 [file 44321_2025_220_MOESM5_ESM.zip › Figure 2/Figure 2F/β-actin.tif]

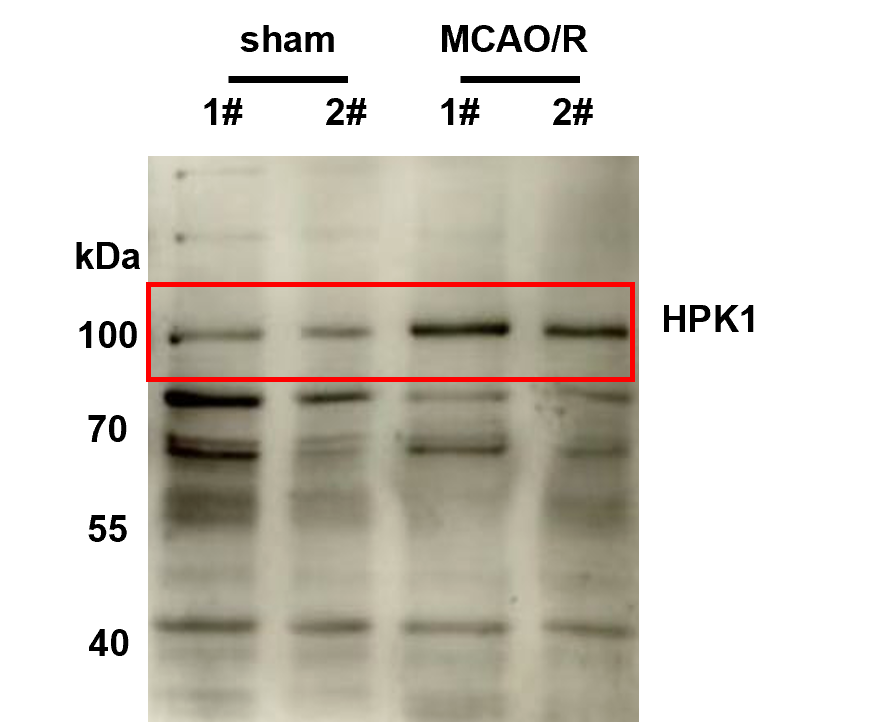

Supplement: Supplementary file 6 — Source data Fig. 3 [file 44321_2025_220_MOESM6_ESM.zip › Figure 3/Figure 3A/HPK1.tif]

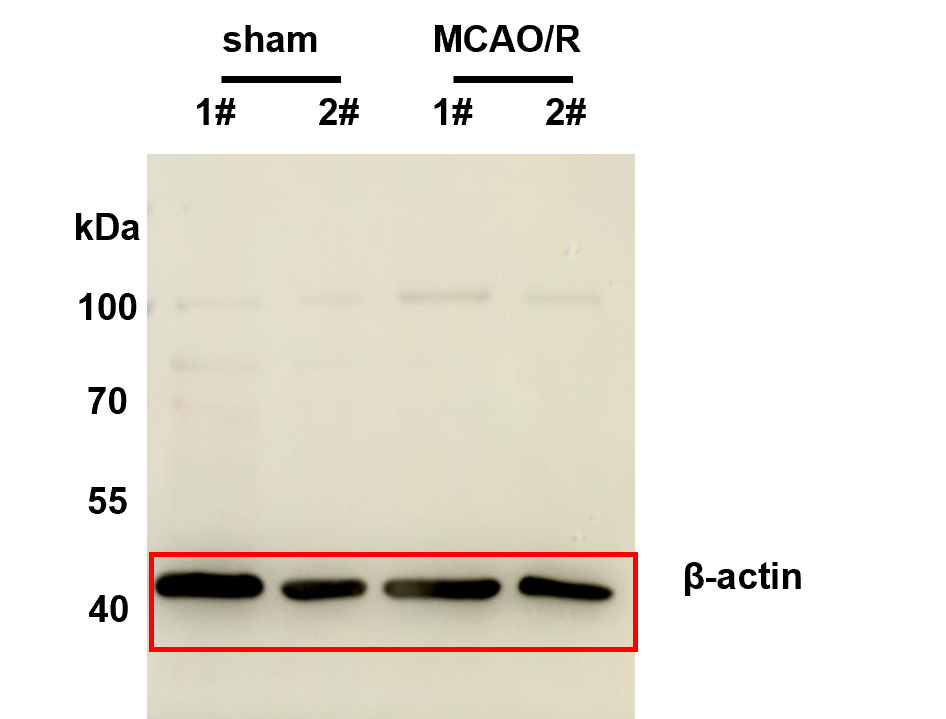

Supplement: Supplementary file 6 — Source data Fig. 3 [file 44321_2025_220_MOESM6_ESM.zip › Figure 3/Figure 3A/β-actin.tif]

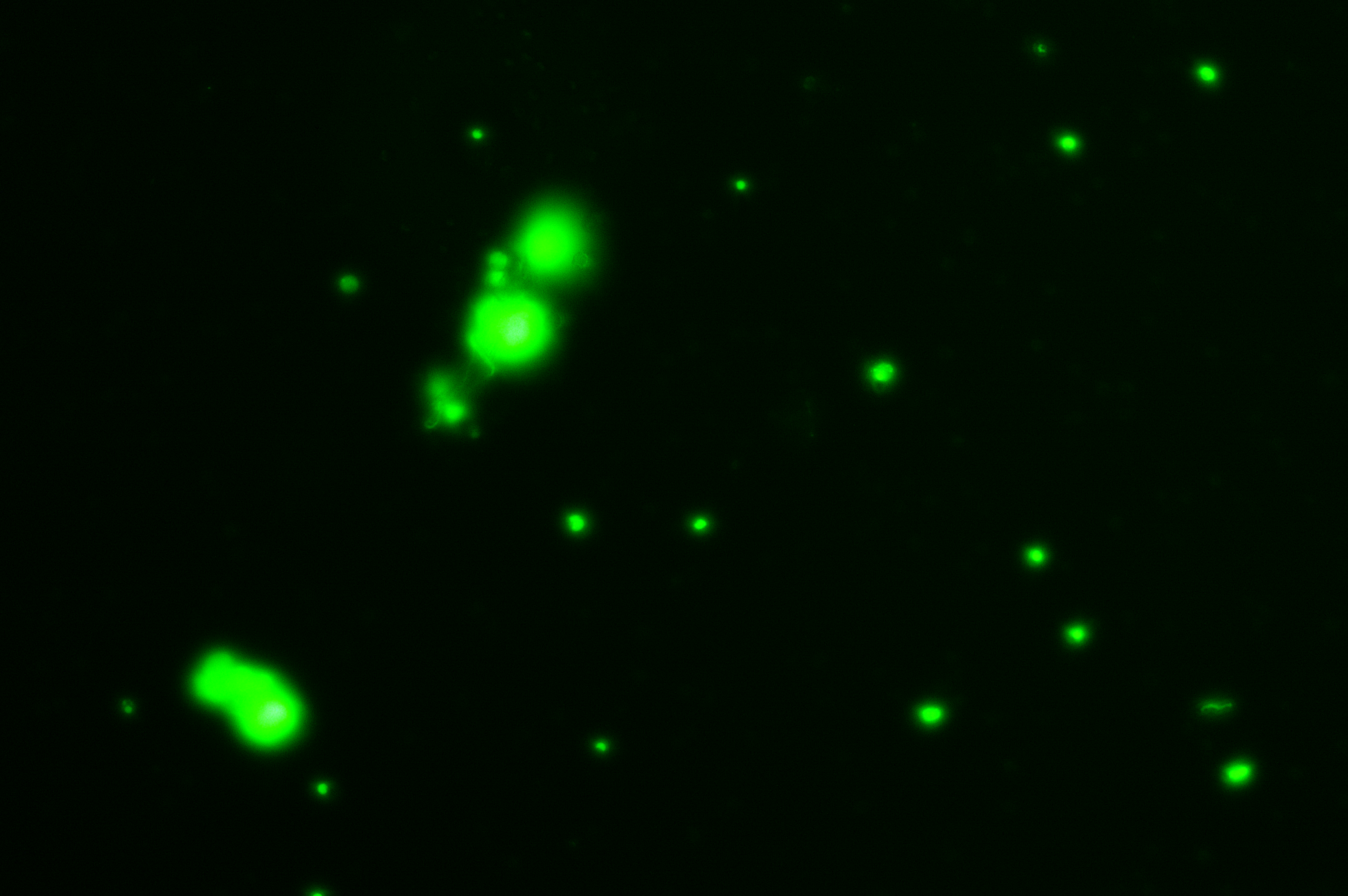

Supplement: Supplementary file 6 — Source data Fig. 3 [file 44321_2025_220_MOESM6_ESM.zip › Figure 3/Figure 3K/KO MCAO/sytox green.tif]

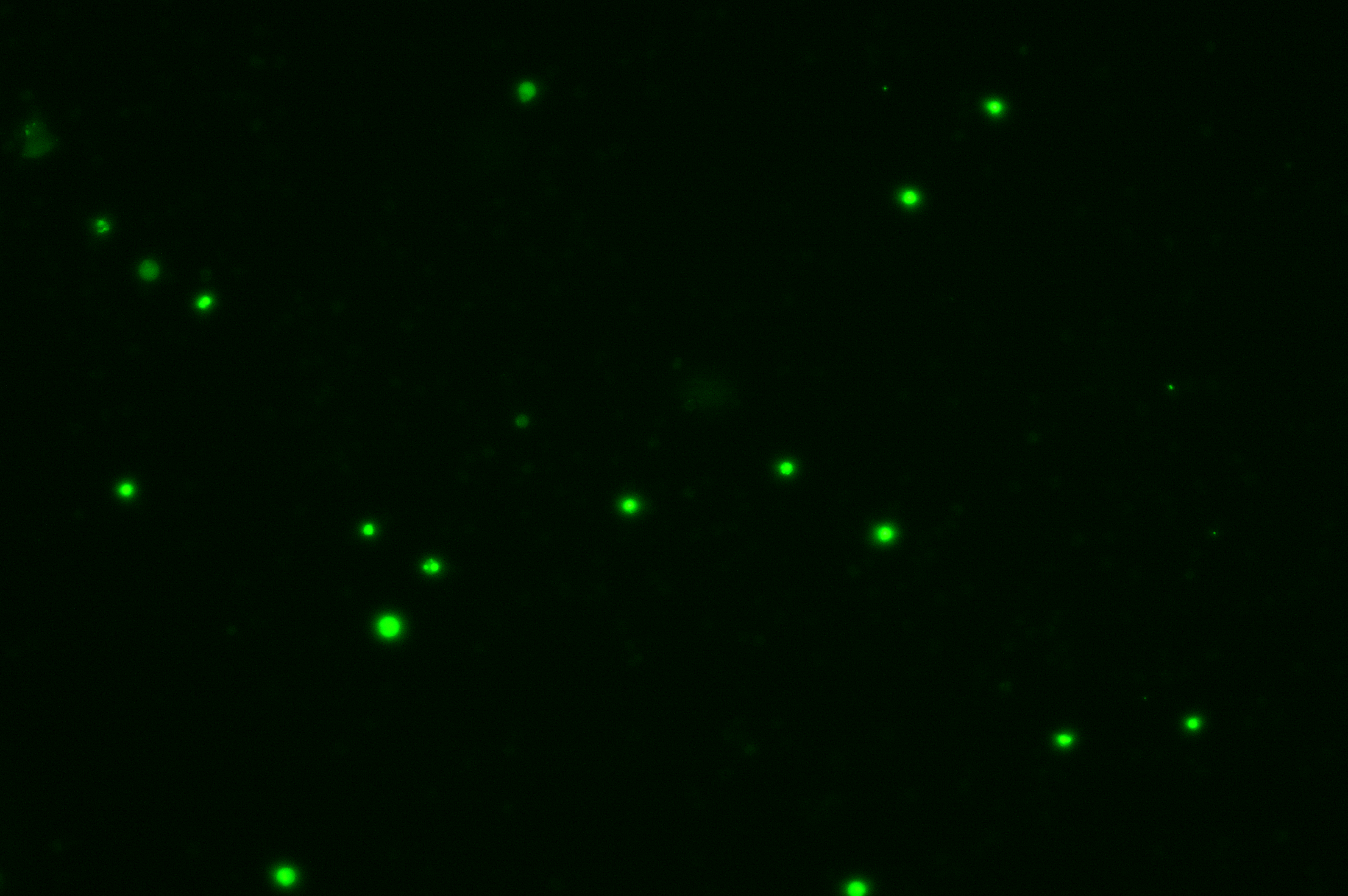

Supplement: Supplementary file 6 — Source data Fig. 3 [file 44321_2025_220_MOESM6_ESM.zip › Figure 3/Figure 3K/KO sham/sytox green.tif]

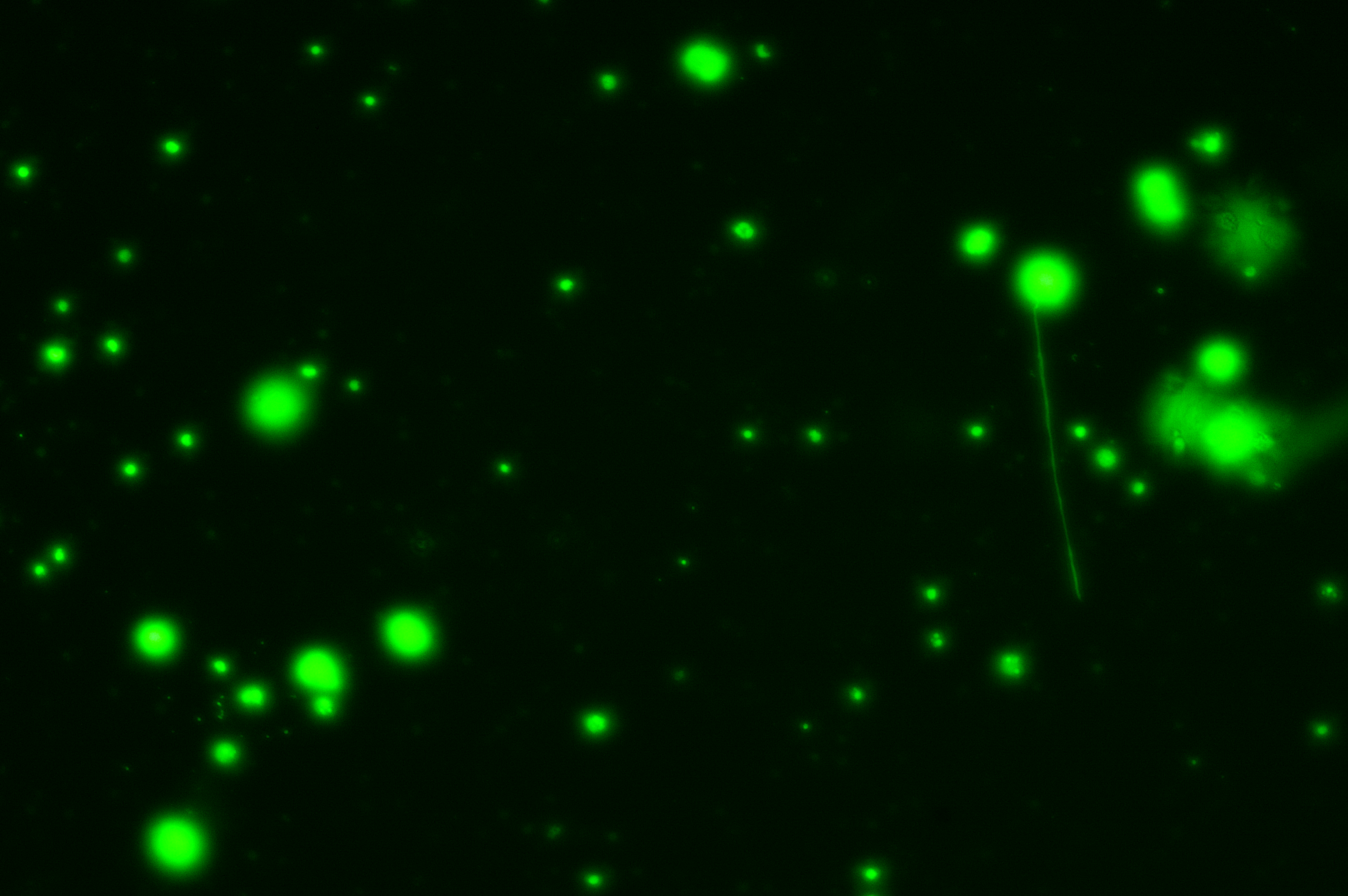

Supplement: Supplementary file 6 — Source data Fig. 3 [file 44321_2025_220_MOESM6_ESM.zip › Figure 3/Figure 3K/WT MCAO/sytox green.tif]

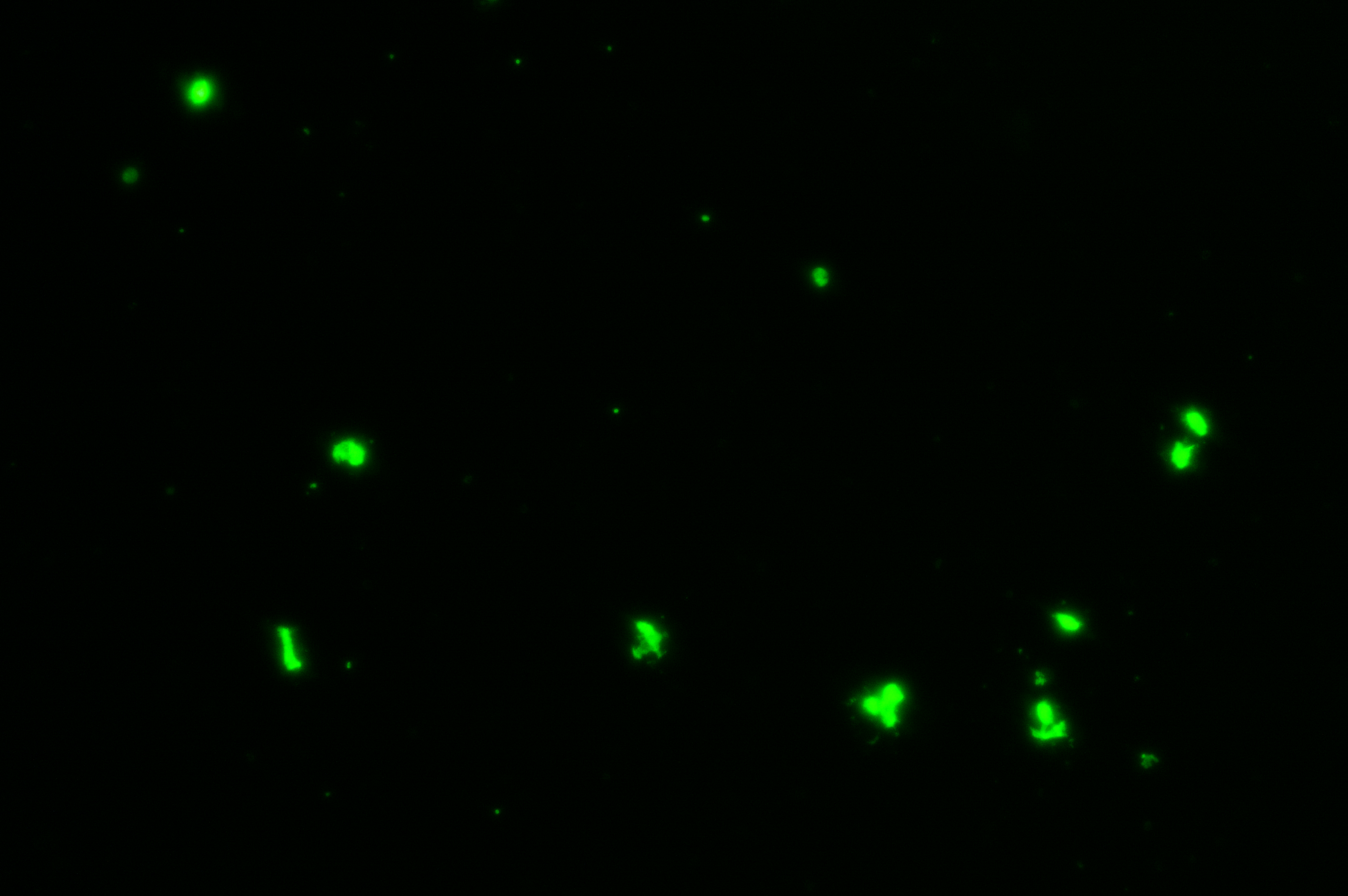

Supplement: Supplementary file 6 — Source data Fig. 3 [file 44321_2025_220_MOESM6_ESM.zip › Figure 3/Figure 3K/WT sham/sytox green .tif]

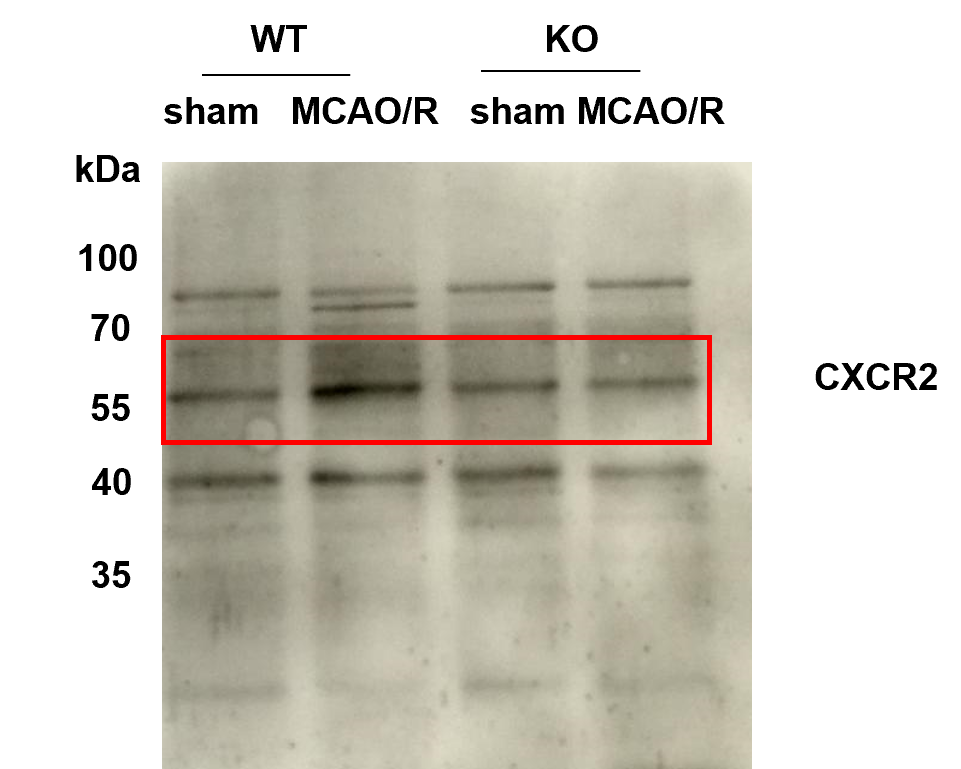

Supplement: Supplementary file 7 — Source data Fig. 4 [file 44321_2025_220_MOESM7_ESM.zip › Figure 4/Figure 4G/CXCR2.tif]

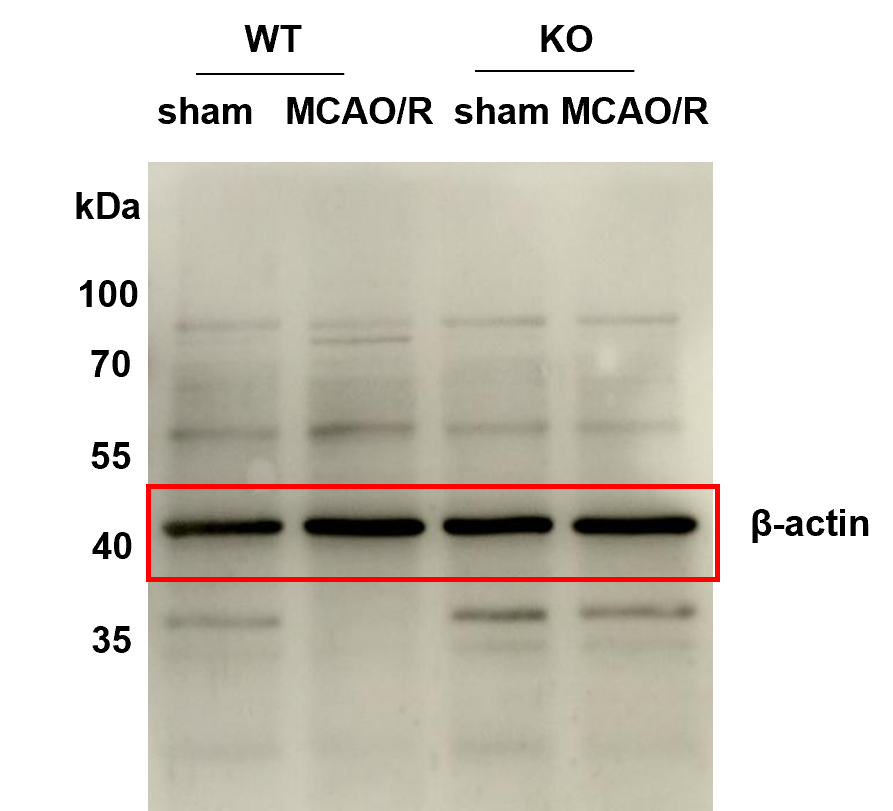

Supplement: Supplementary file 7 — Source data Fig. 4 [file 44321_2025_220_MOESM7_ESM.zip › Figure 4/Figure 4G/β-actin.tif]

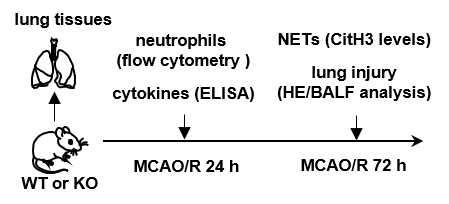

Supplement: Supplementary file 8 — Source data Fig. 5 [file 44321_2025_220_MOESM8_ESM.zip › EMM-2024-20638-V3_Figure 5/Figure 5A/figure 5A.png]

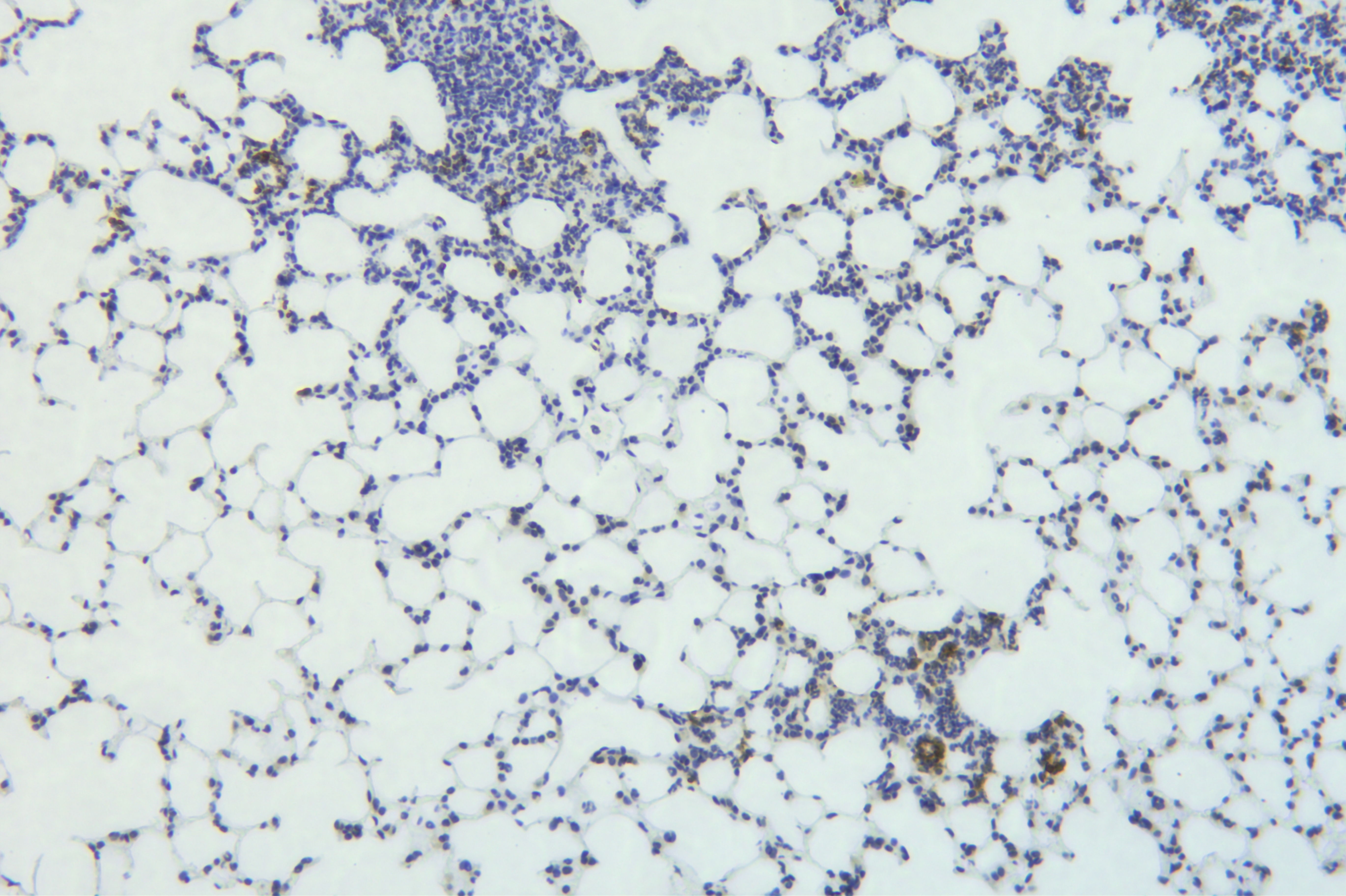

Supplement: Supplementary file 8 — Source data Fig. 5 [file 44321_2025_220_MOESM8_ESM.zip › EMM-2024-20638-V3_Figure 5/Figure 5G/female KO MCAO/female KO MCAO.jpg]

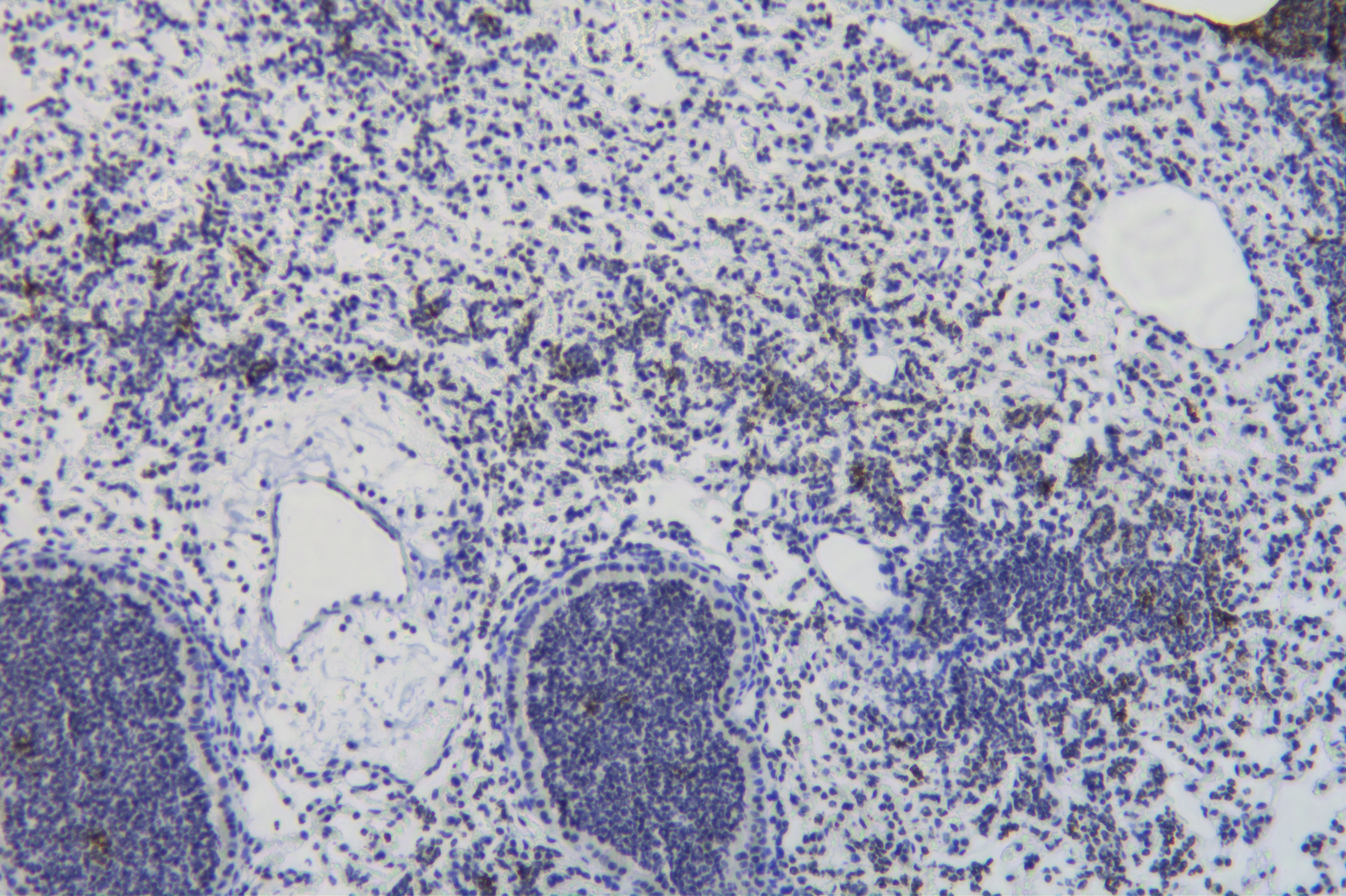

Supplement: Supplementary file 8 — Source data Fig. 5 [file 44321_2025_220_MOESM8_ESM.zip › EMM-2024-20638-V3_Figure 5/Figure 5G/female WT MCAO/female WT MCAO.jpg]

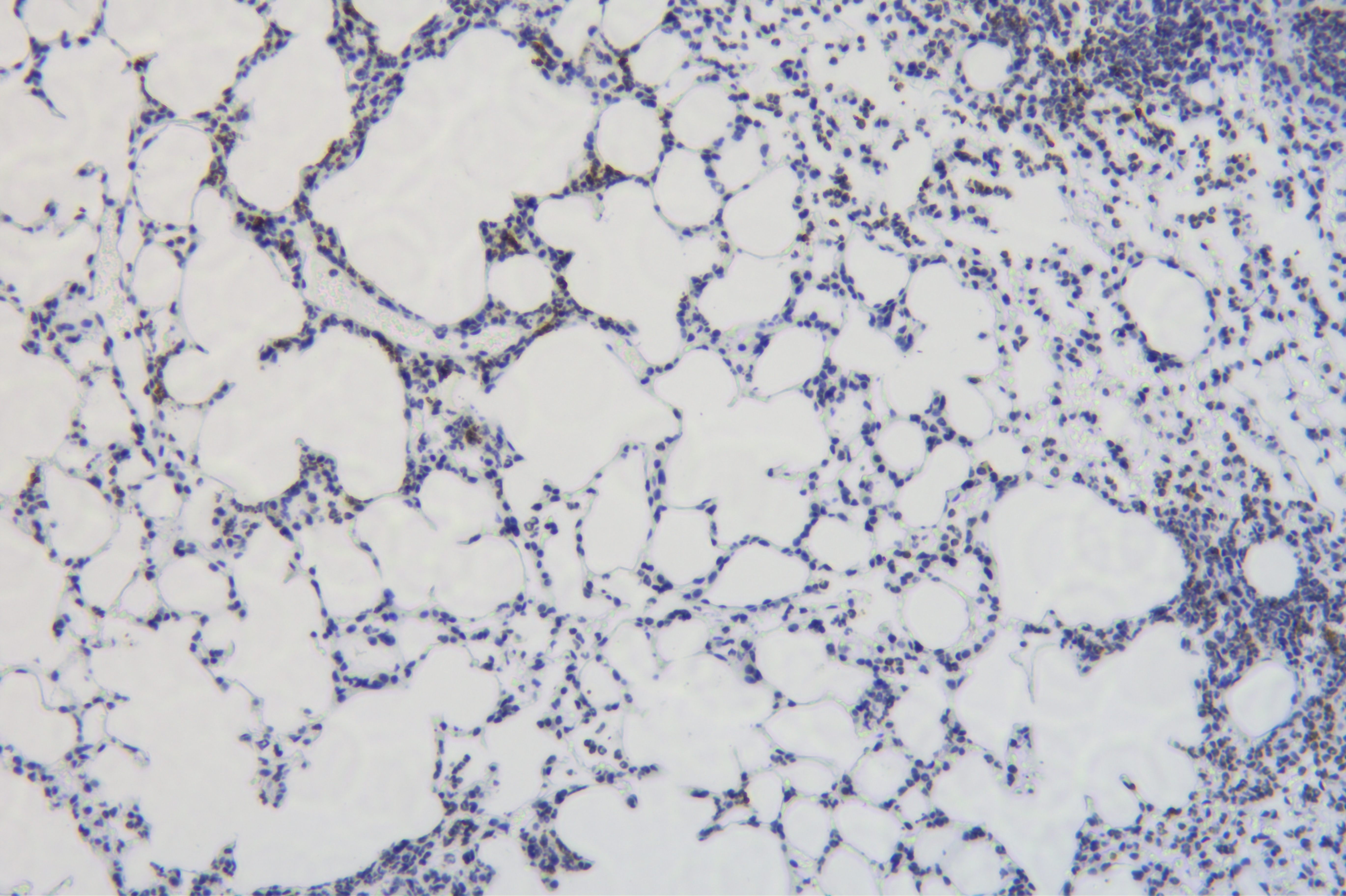

Supplement: Supplementary file 8 — Source data Fig. 5 [file 44321_2025_220_MOESM8_ESM.zip › EMM-2024-20638-V3_Figure 5/Figure 5G/male KO MCAO/male KO MCAO CitH3.jpg]

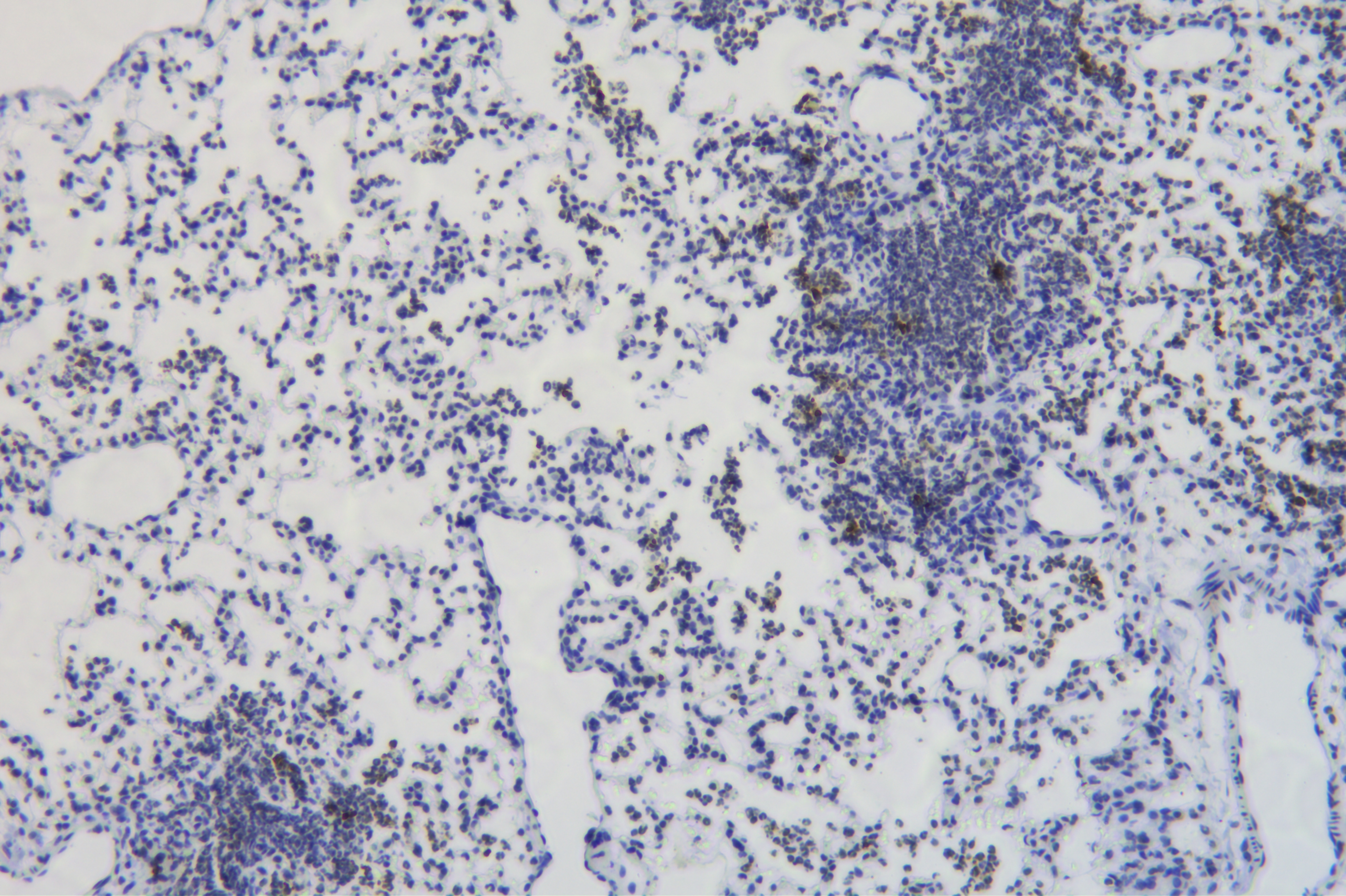

Supplement: Supplementary file 8 — Source data Fig. 5 [file 44321_2025_220_MOESM8_ESM.zip › EMM-2024-20638-V3_Figure 5/Figure 5G/male WT MCAO/male WT MCAO CitH3.jpg]

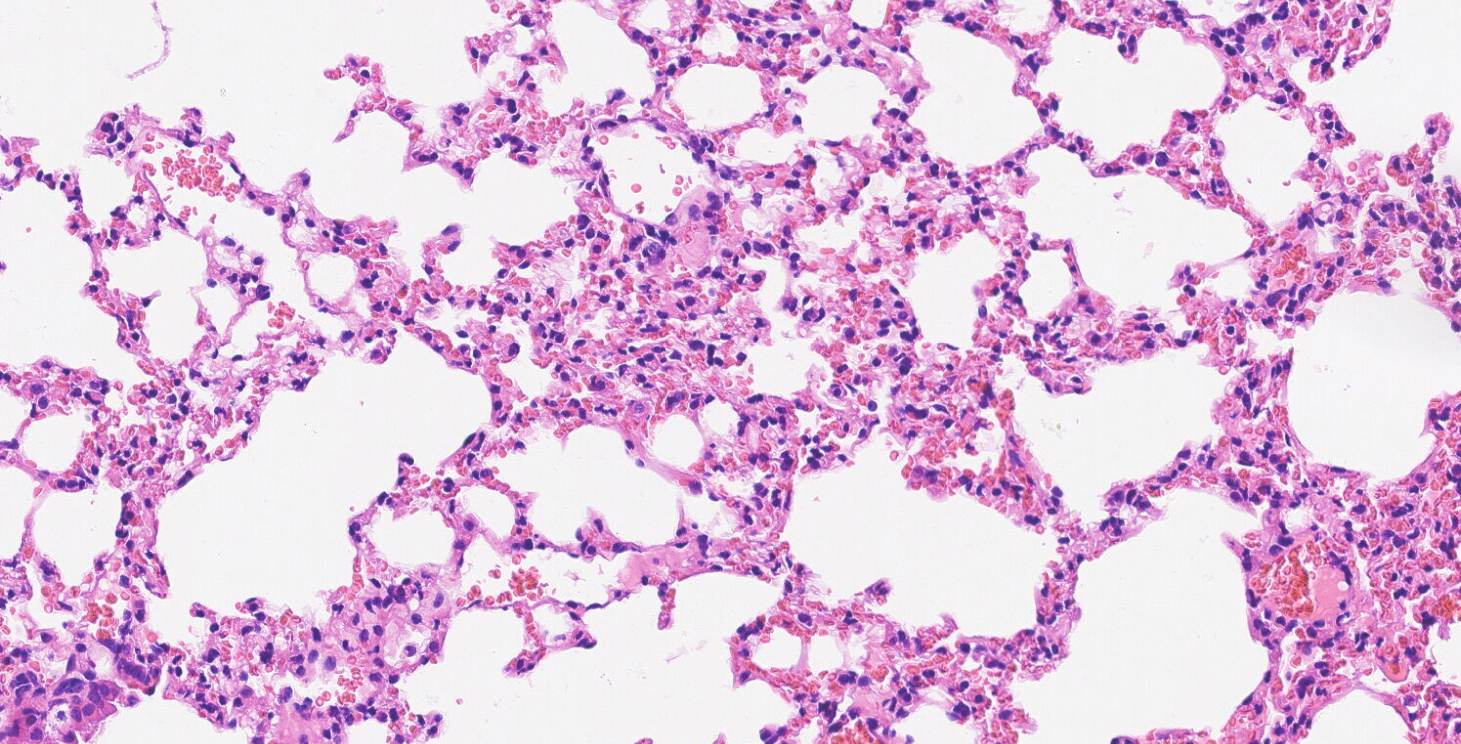

Supplement: Supplementary file 8 — Source data Fig. 5 [file 44321_2025_220_MOESM8_ESM.zip › EMM-2024-20638-V3_Figure 5/Figure 5I/female KO MCAO/KO MCAO.jpg]

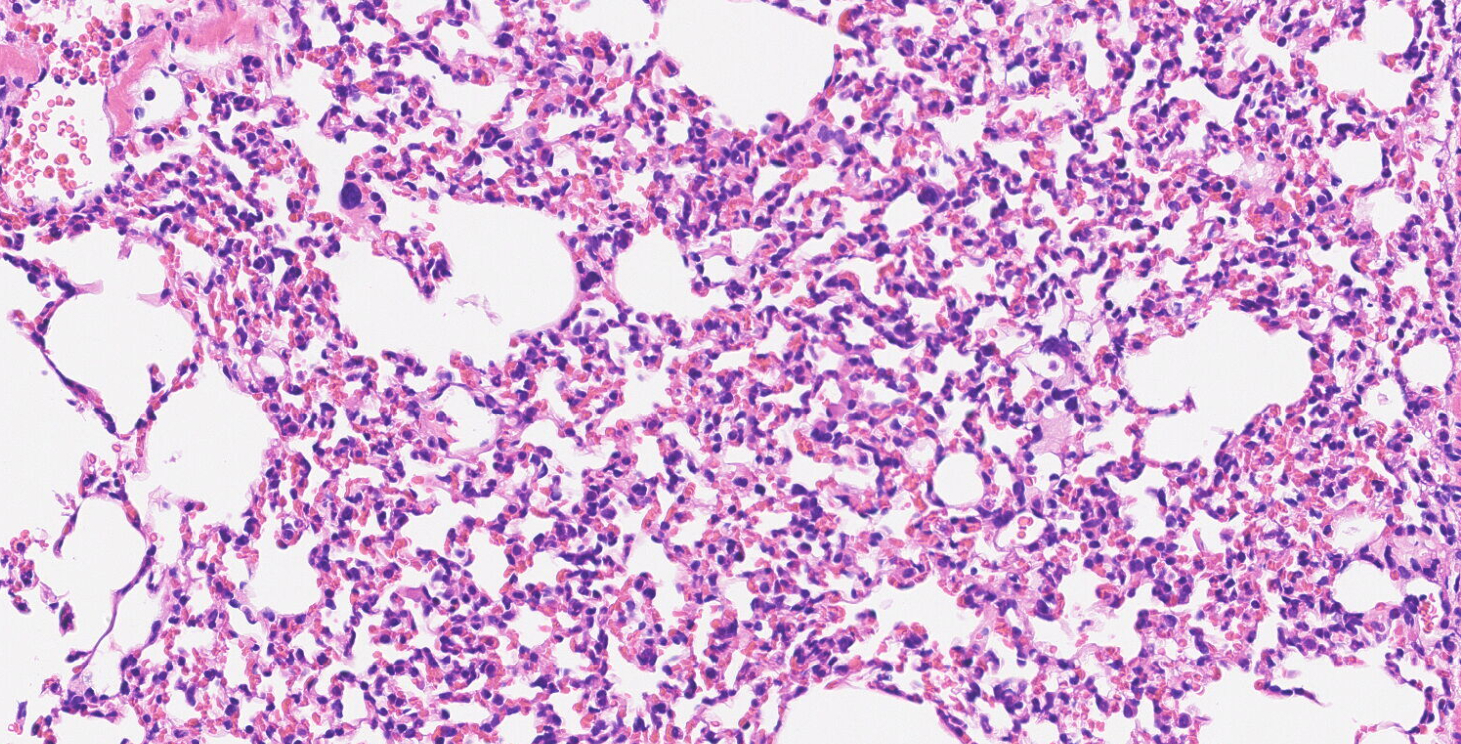

Supplement: Supplementary file 8 — Source data Fig. 5 [file 44321_2025_220_MOESM8_ESM.zip › EMM-2024-20638-V3_Figure 5/Figure 5I/female WT MCAO/WT MCAO .jpg]

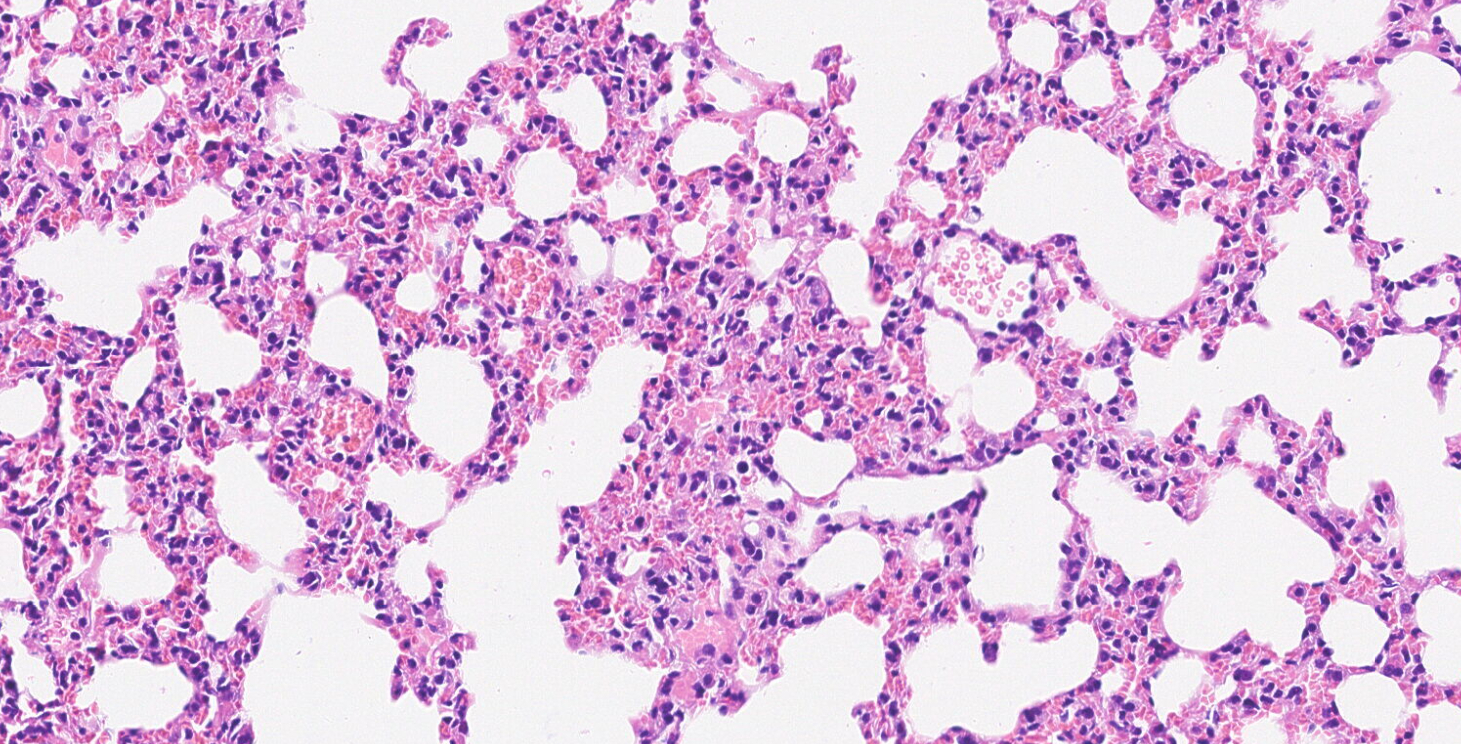

Supplement: Supplementary file 8 — Source data Fig. 5 [file 44321_2025_220_MOESM8_ESM.zip › EMM-2024-20638-V3_Figure 5/Figure 5I/male KO MCAO/KO MCAO.jpg]

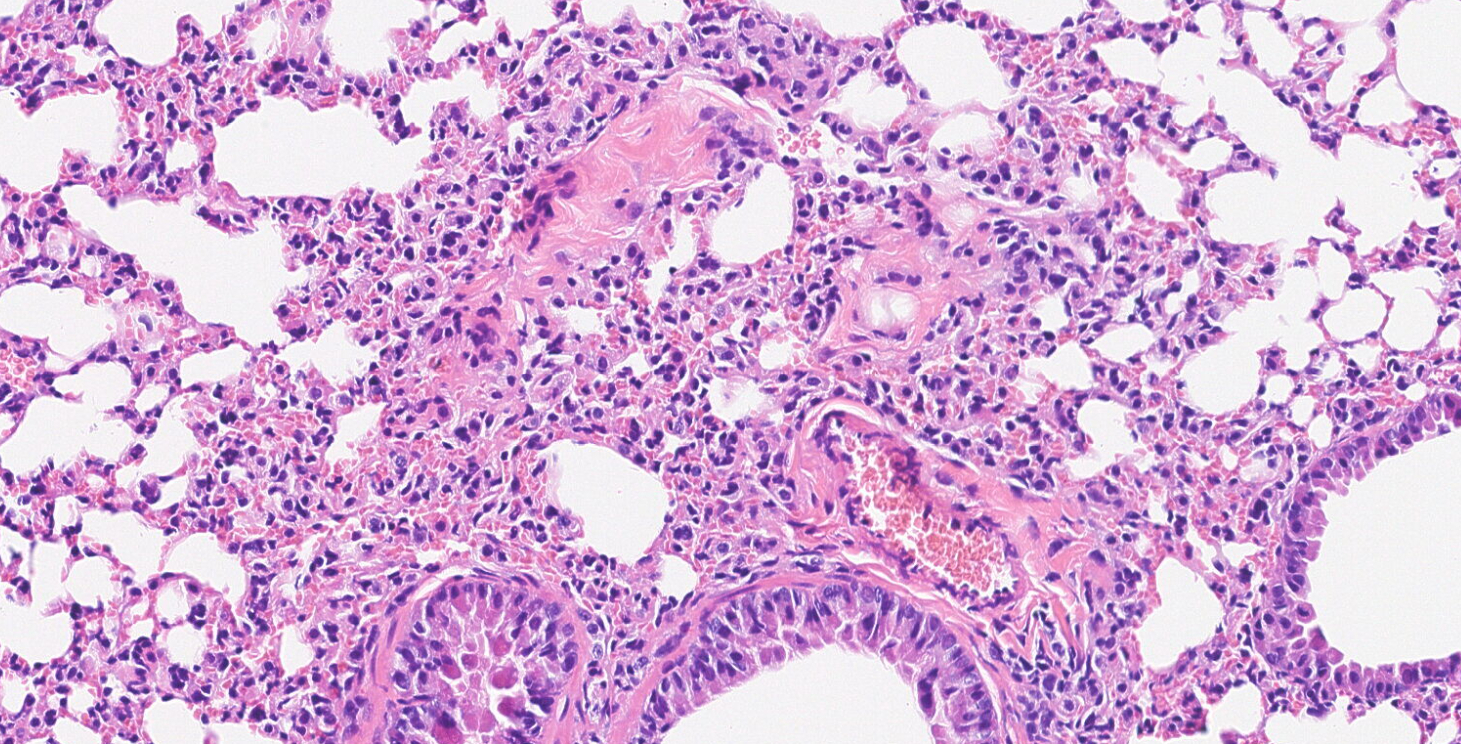

Supplement: Supplementary file 8 — Source data Fig. 5 [file 44321_2025_220_MOESM8_ESM.zip › EMM-2024-20638-V3_Figure 5/Figure 5I/male WT MCAO/male WT MCAO.jpg]

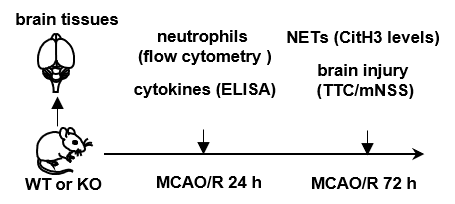

Supplement: Supplementary file 9 — Source data Fig. 6 [file 44321_2025_220_MOESM9_ESM.zip › EMM-2024-20638-V3_Figure 6/Figure 6A/Figure 6A.png]

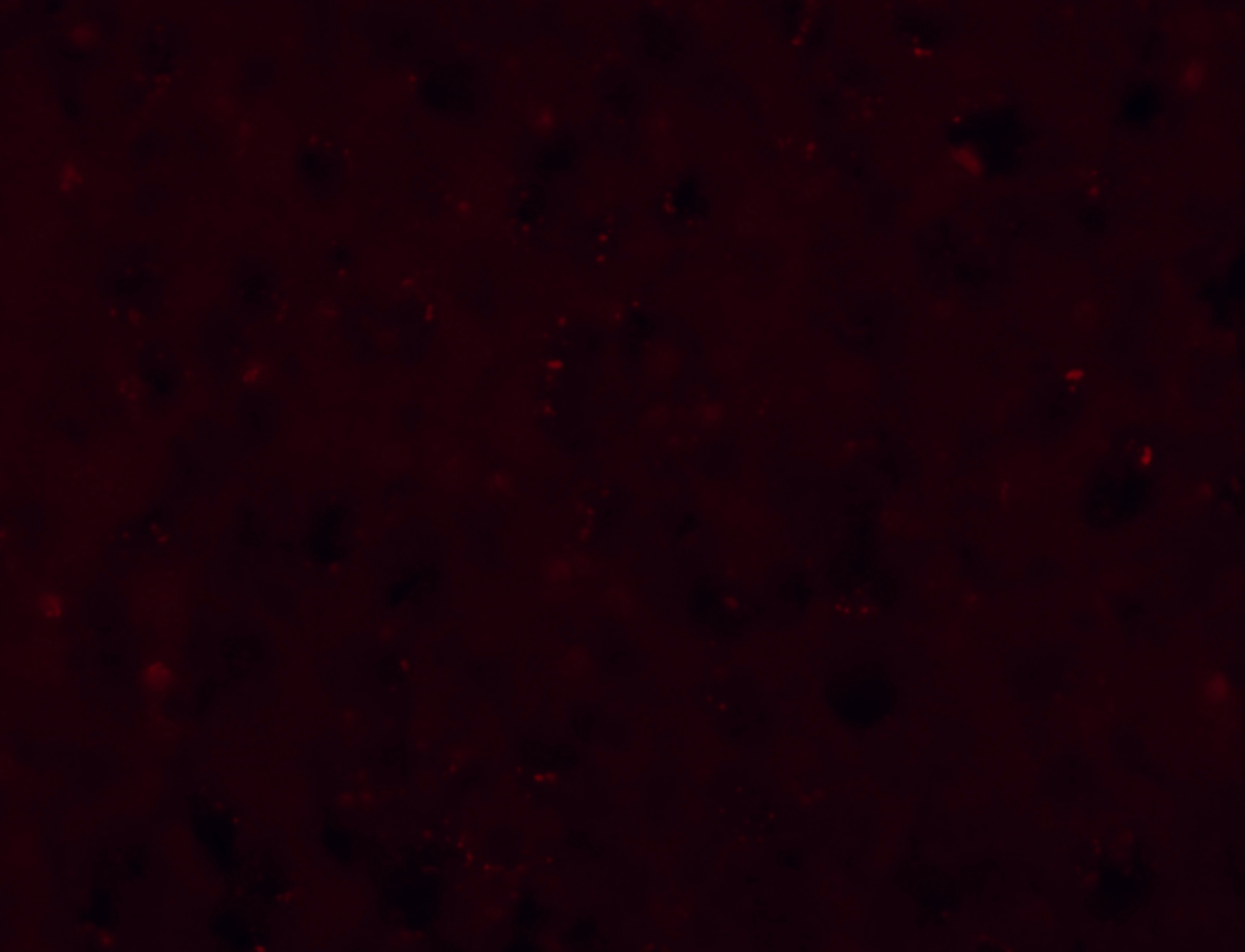

Supplement: Supplementary file 9 — Source data Fig. 6 [file 44321_2025_220_MOESM9_ESM.zip › EMM-2024-20638-V3_Figure 6/Figure 6G/KO control/CitH3-KO-control.png]

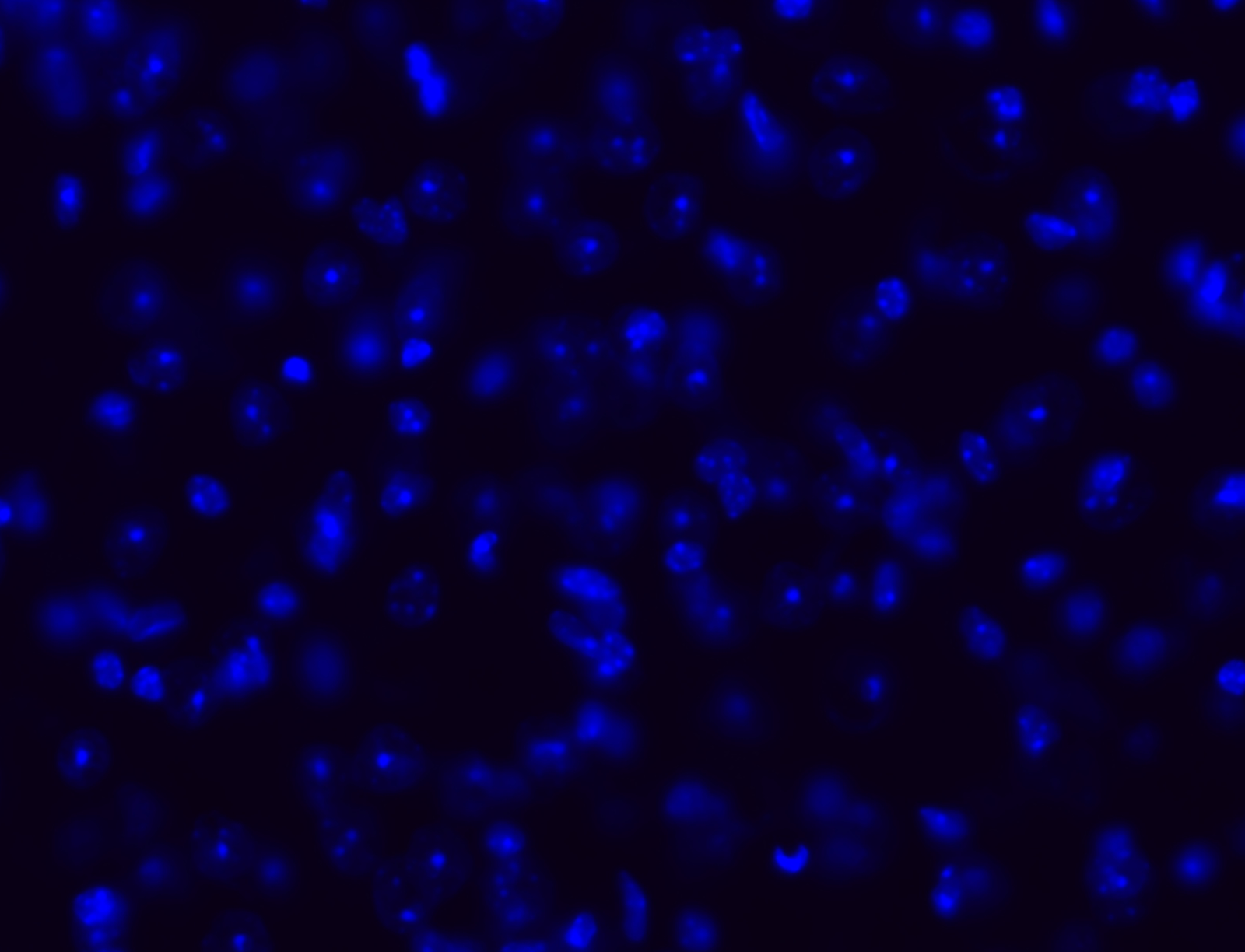

Supplement: Supplementary file 9 — Source data Fig. 6 [file 44321_2025_220_MOESM9_ESM.zip › EMM-2024-20638-V3_Figure 6/Figure 6G/KO control/DAPI-KO-control.png]

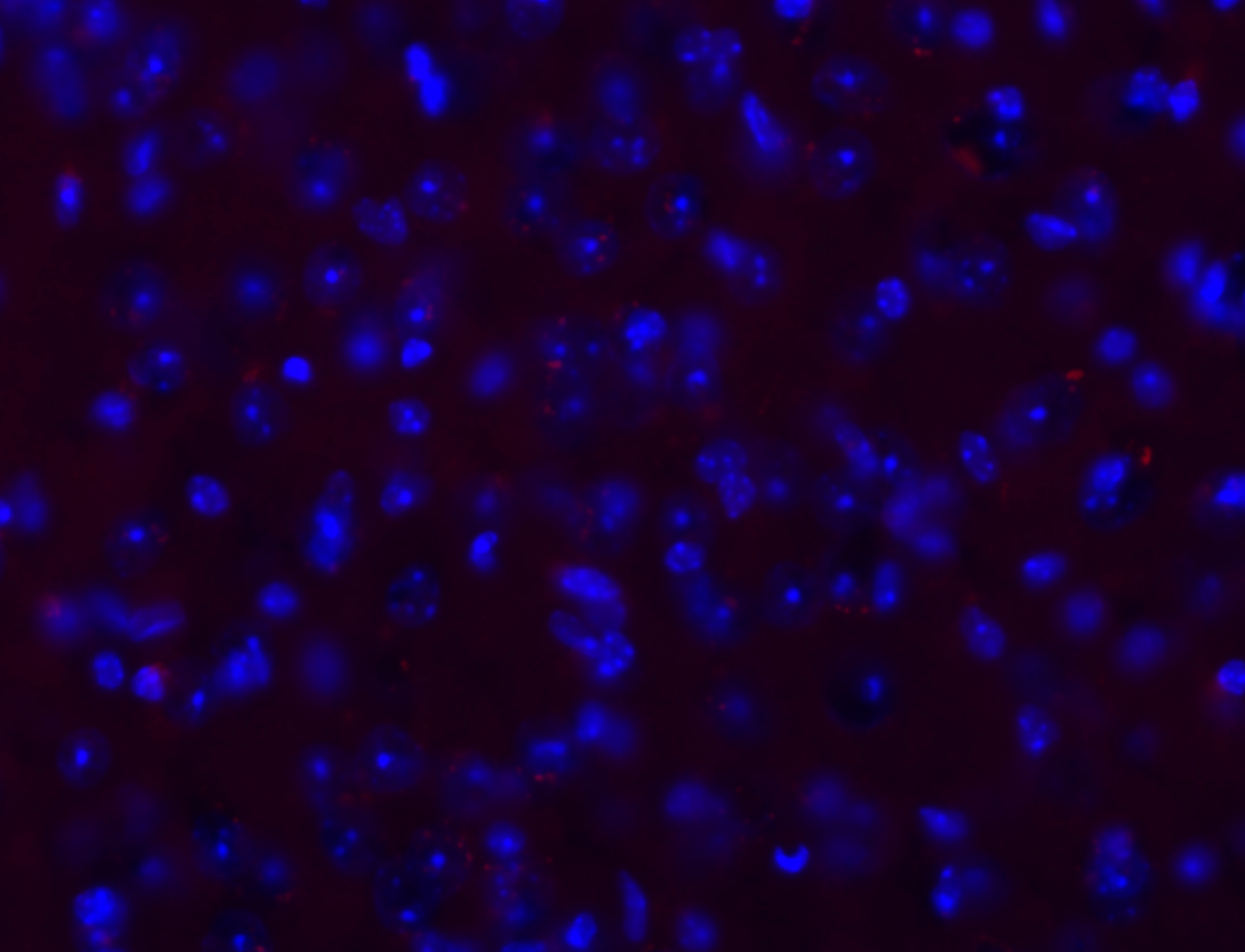

Supplement: Supplementary file 9 — Source data Fig. 6 [file 44321_2025_220_MOESM9_ESM.zip › EMM-2024-20638-V3_Figure 6/Figure 6G/KO control/MERGE-KO-control.png]

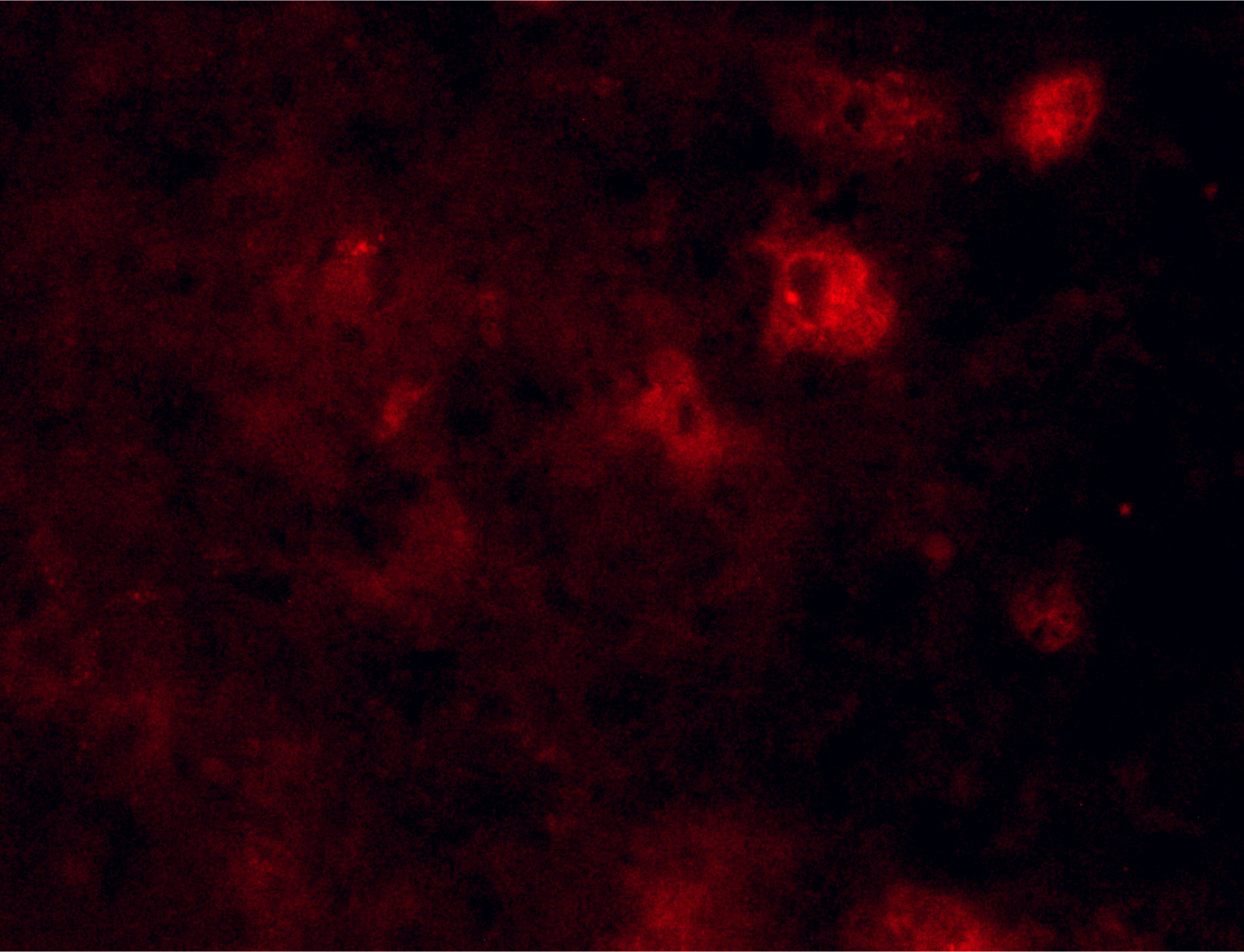

Supplement: Supplementary file 9 — Source data Fig. 6 [file 44321_2025_220_MOESM9_ESM.zip › EMM-2024-20638-V3_Figure 6/Figure 6G/KO MCAO/CitH3-KO-MCAO.png]

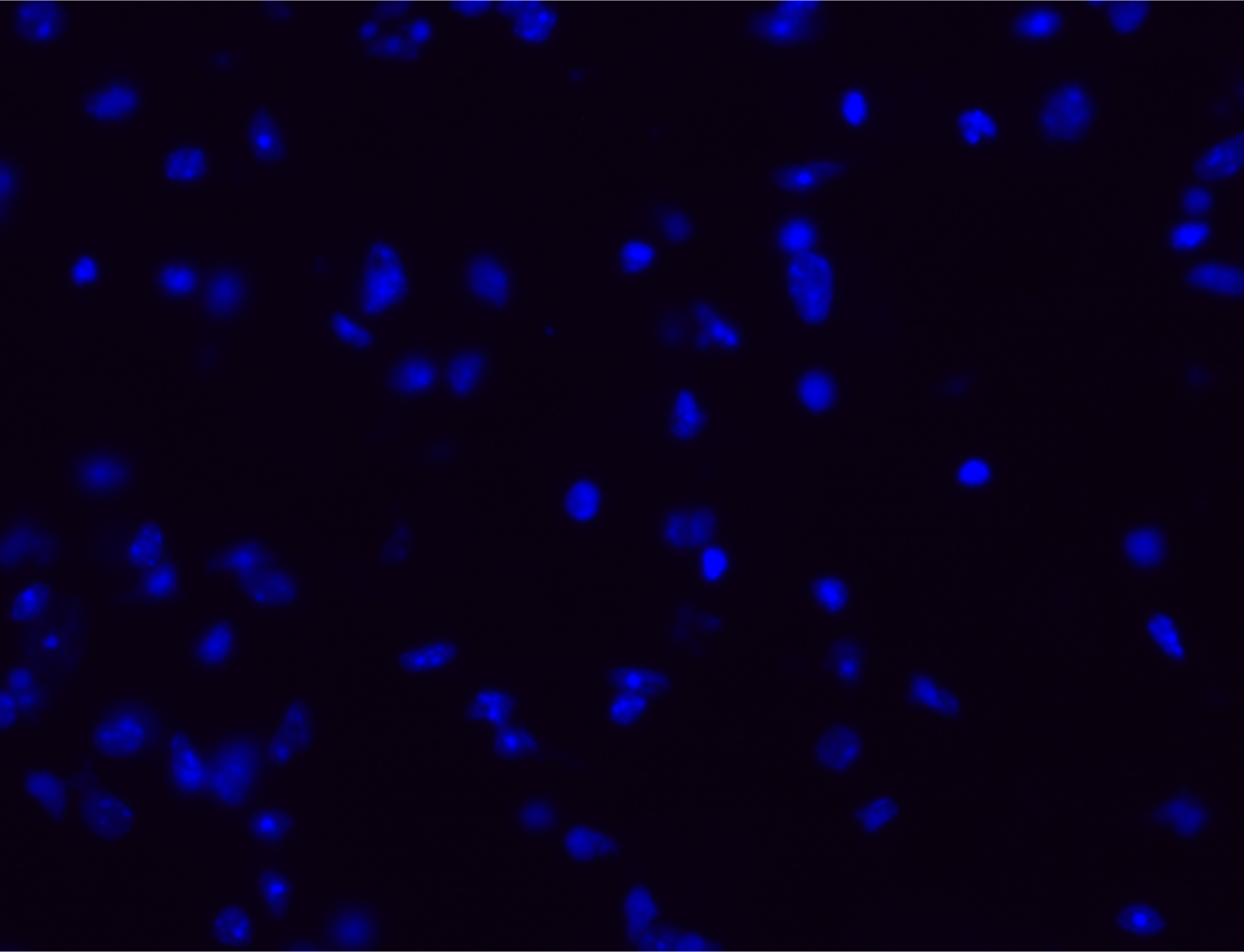

Supplement: Supplementary file 9 — Source data Fig. 6 [file 44321_2025_220_MOESM9_ESM.zip › EMM-2024-20638-V3_Figure 6/Figure 6G/KO MCAO/DAPI-KO-MCAO.png]

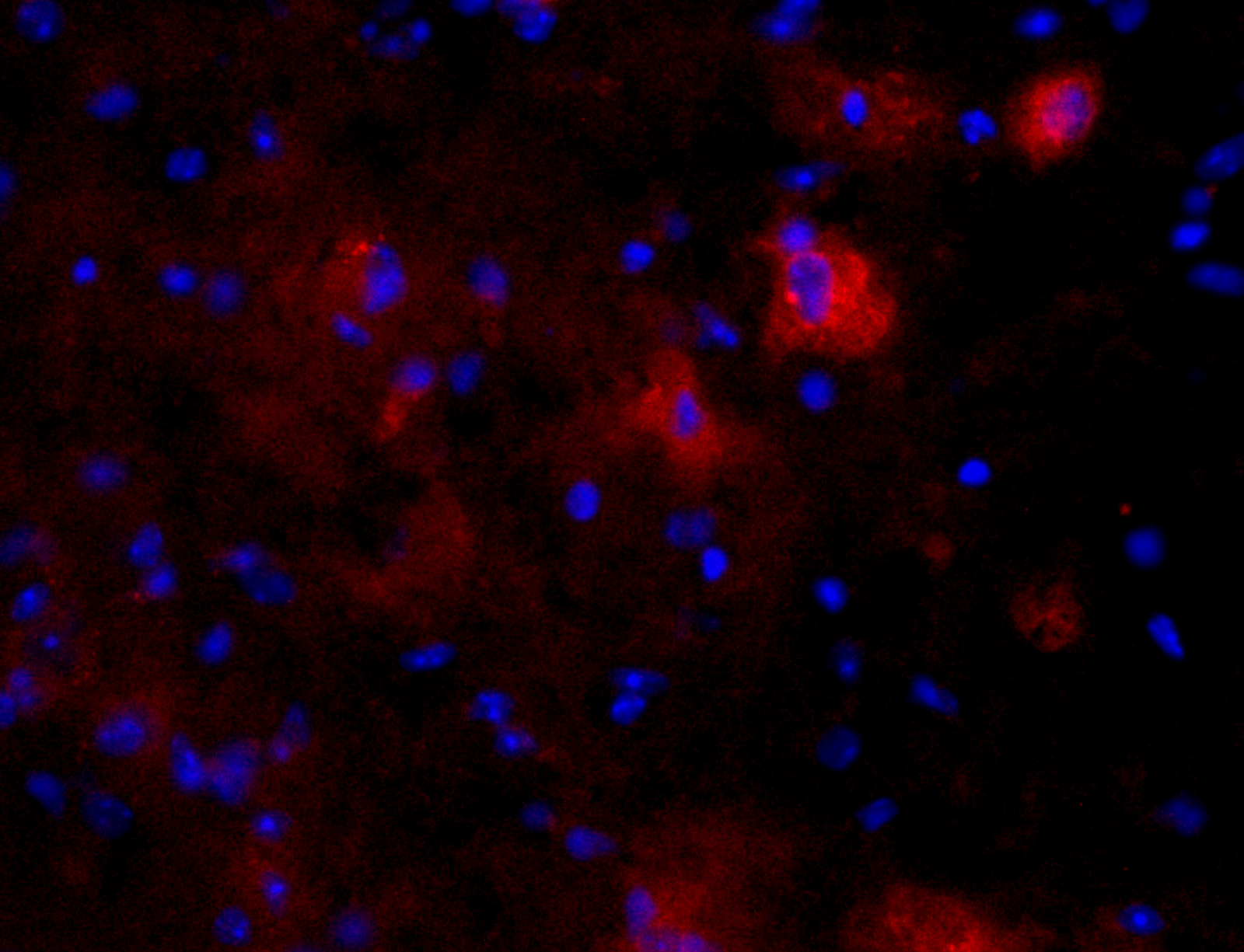

Supplement: Supplementary file 9 — Source data Fig. 6 [file 44321_2025_220_MOESM9_ESM.zip › EMM-2024-20638-V3_Figure 6/Figure 6G/KO MCAO/MERGE-KO-MCAO.png]

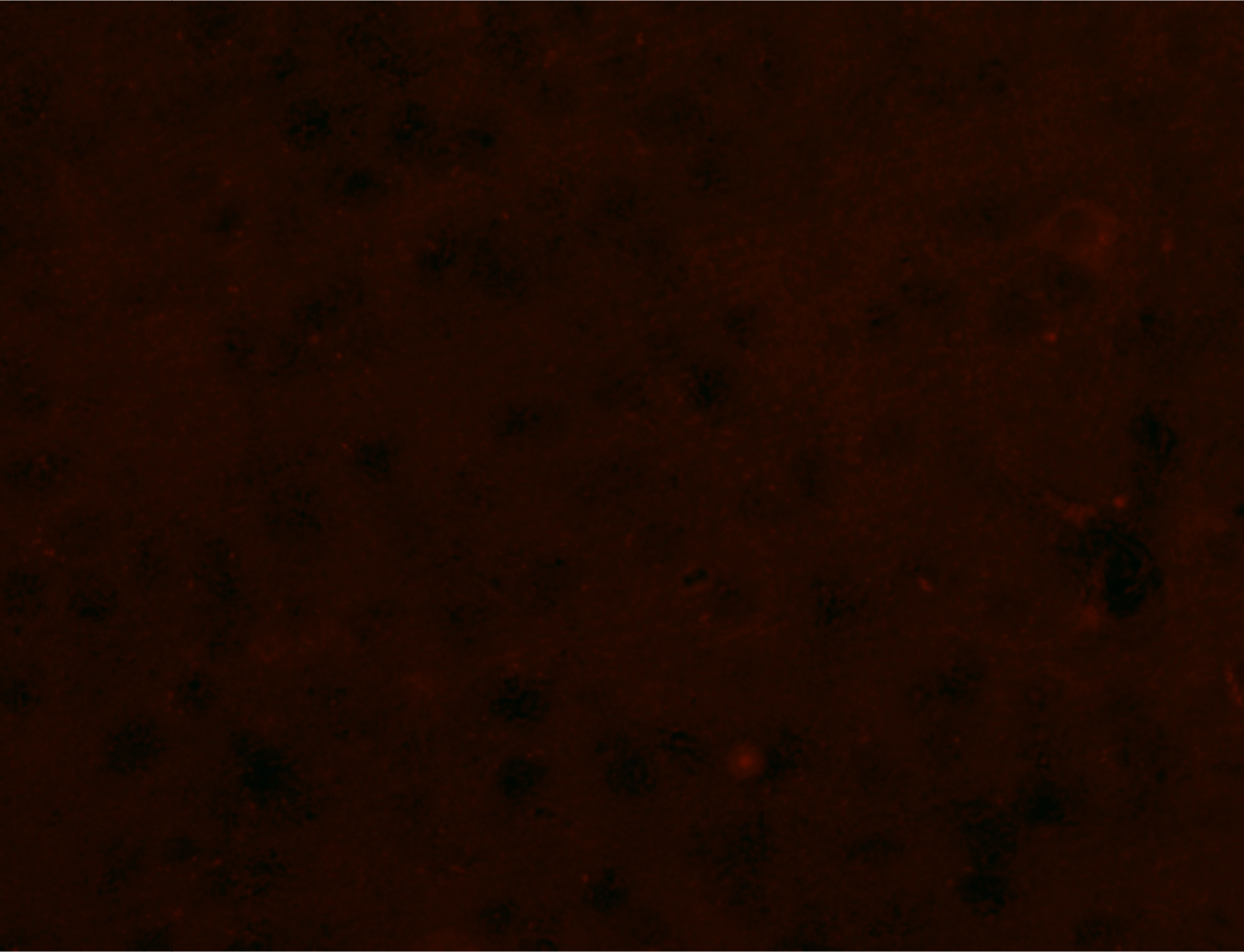

Supplement: Supplementary file 9 — Source data Fig. 6 [file 44321_2025_220_MOESM9_ESM.zip › EMM-2024-20638-V3_Figure 6/Figure 6G/WT control/CitH3-WT-control.png]

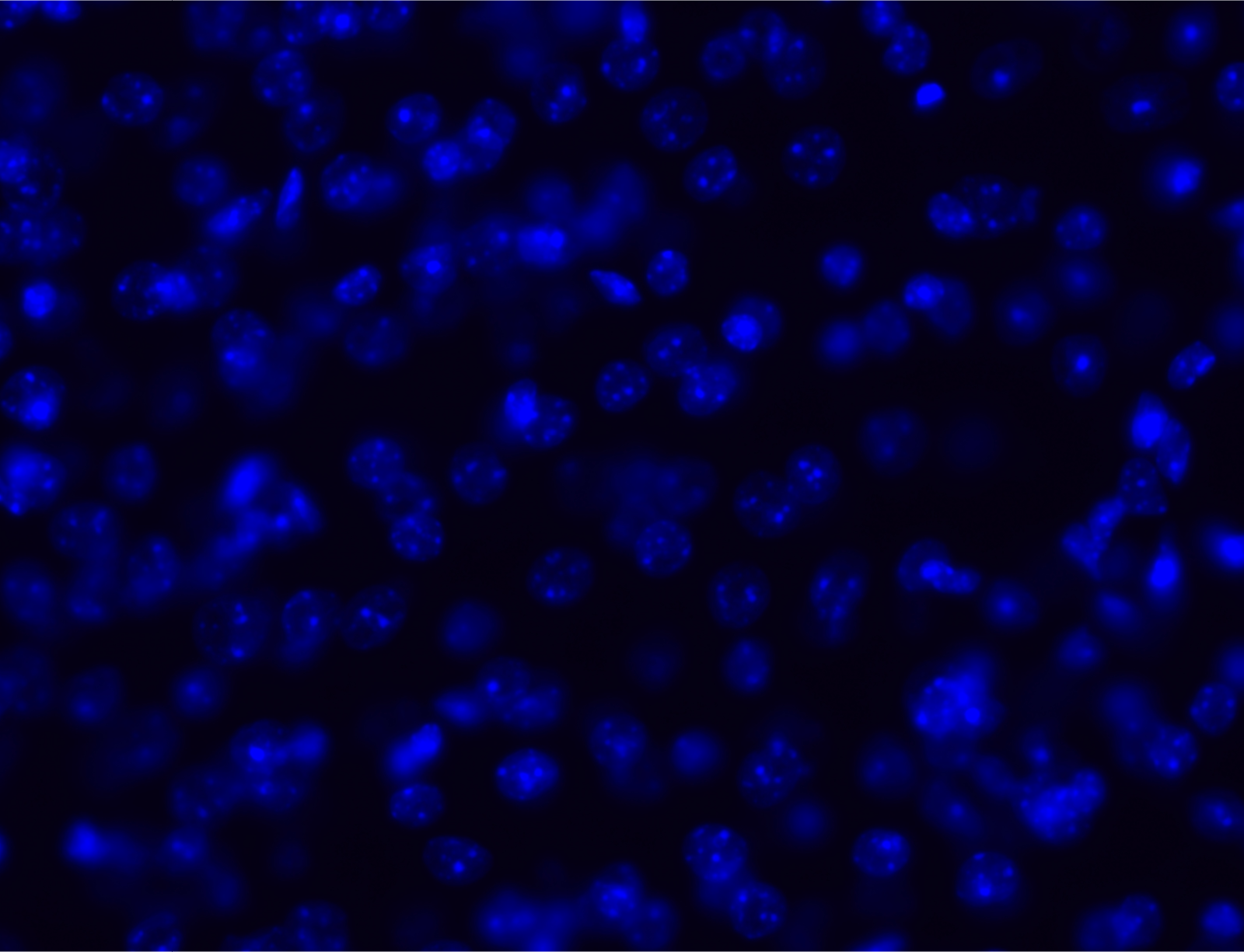

Supplement: Supplementary file 9 — Source data Fig. 6 [file 44321_2025_220_MOESM9_ESM.zip › EMM-2024-20638-V3_Figure 6/Figure 6G/WT control/DAPI-WT-control.png]

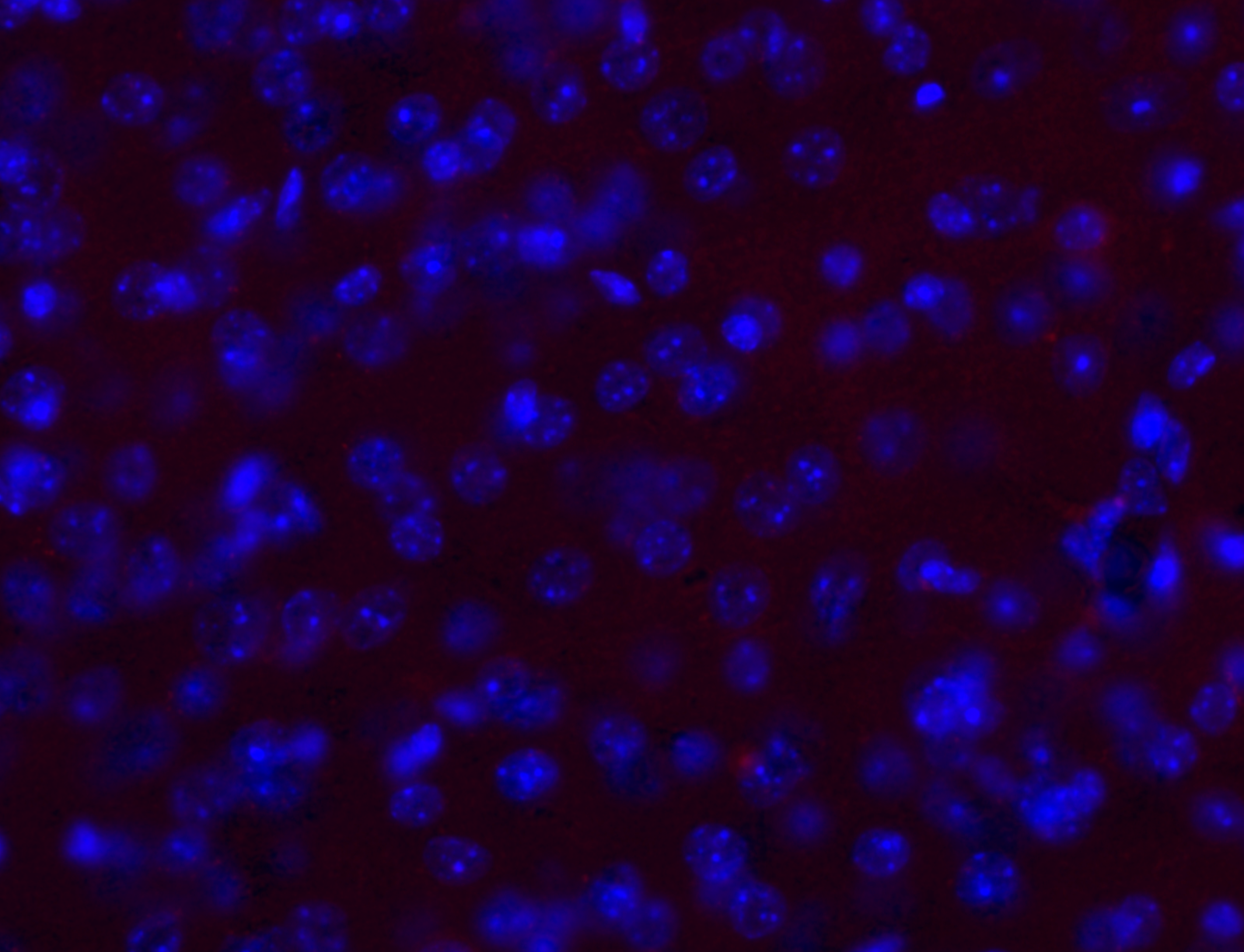

Supplement: Supplementary file 9 — Source data Fig. 6 [file 44321_2025_220_MOESM9_ESM.zip › EMM-2024-20638-V3_Figure 6/Figure 6G/WT control/MERGE-WT-control.png]

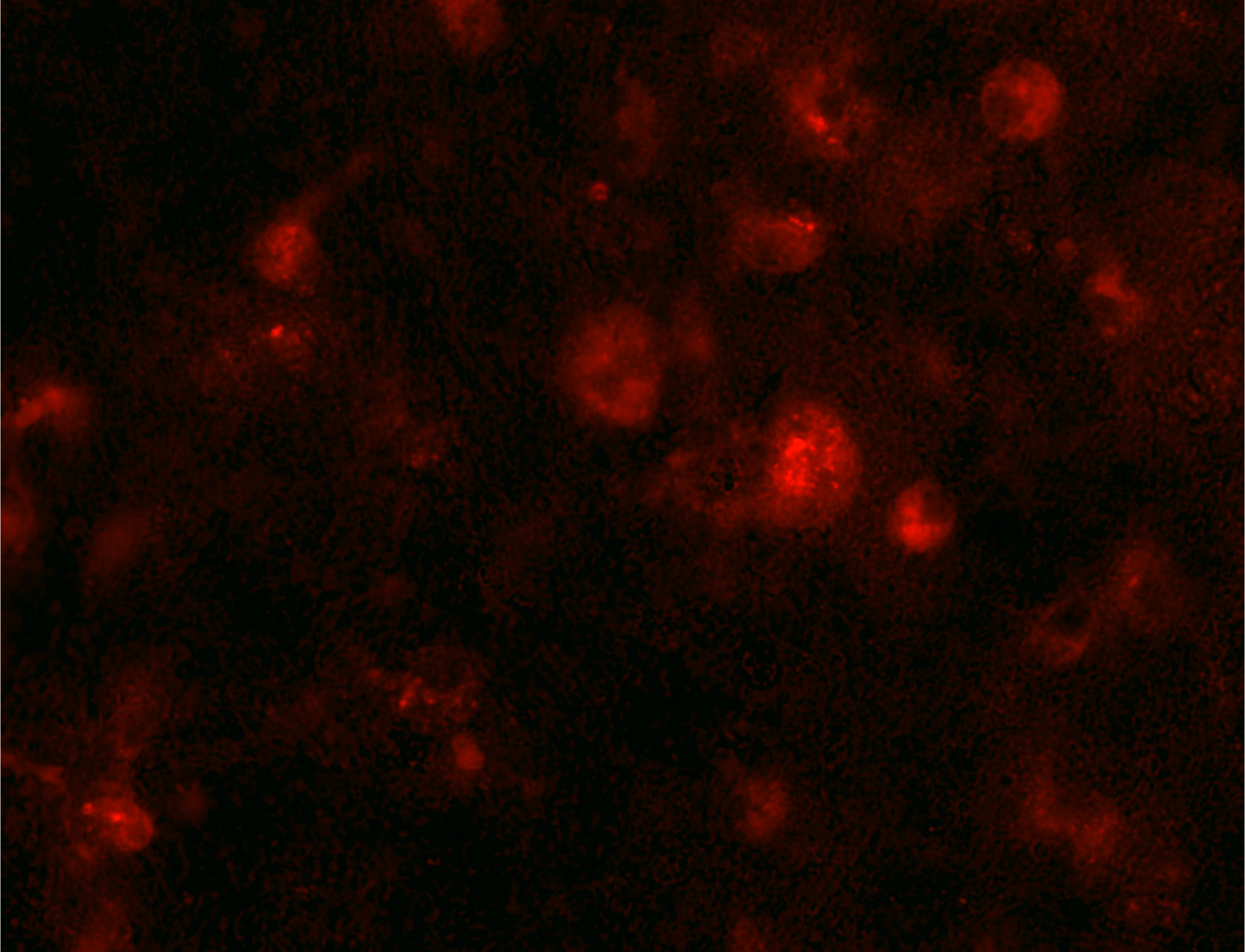

Supplement: Supplementary file 9 — Source data Fig. 6 [file 44321_2025_220_MOESM9_ESM.zip › EMM-2024-20638-V3_Figure 6/Figure 6G/WT MCAO/CitH3-WT-MCAO.png]

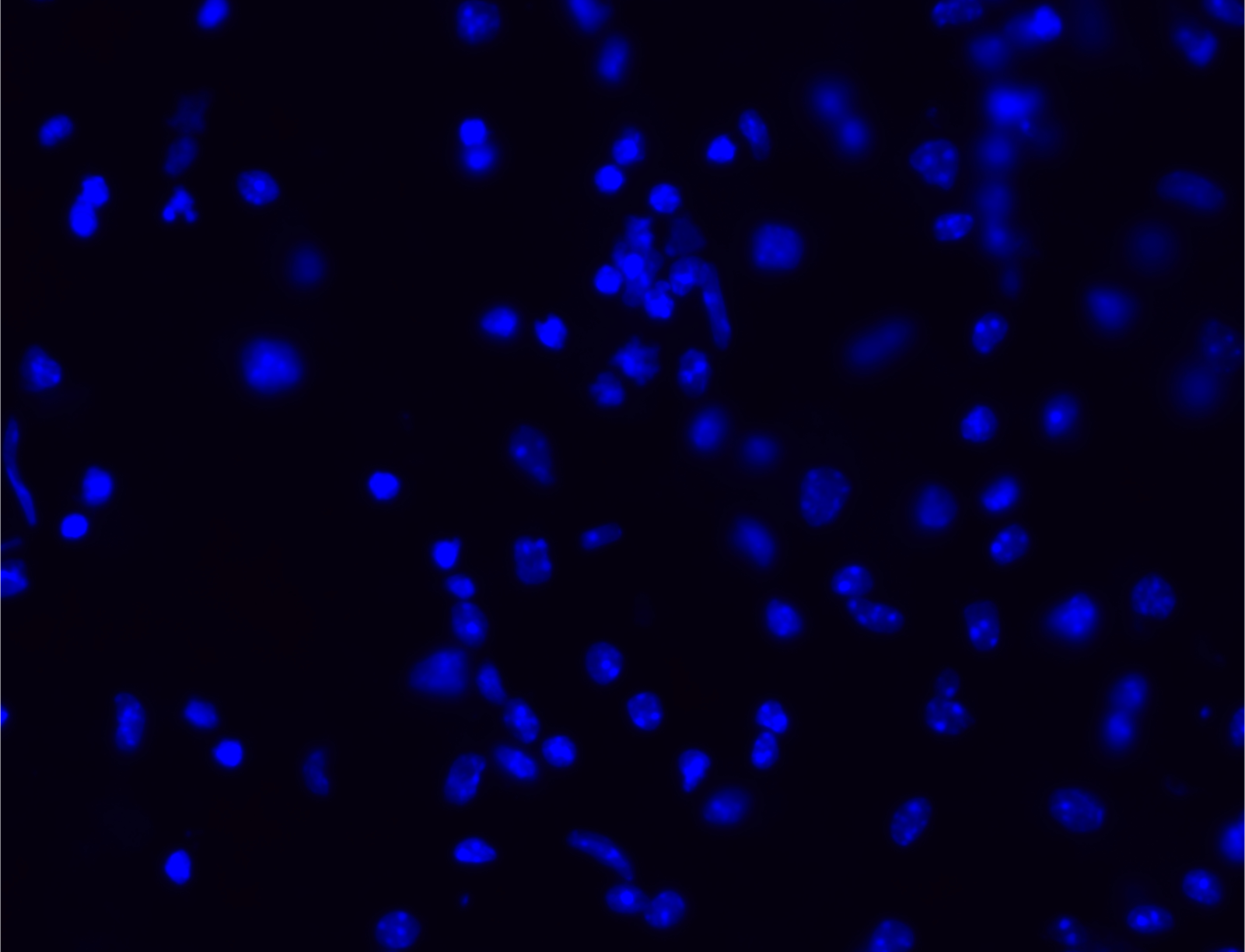

Supplement: Supplementary file 9 — Source data Fig. 6 [file 44321_2025_220_MOESM9_ESM.zip › EMM-2024-20638-V3_Figure 6/Figure 6G/WT MCAO/DAPI-WT-MCAO.png]

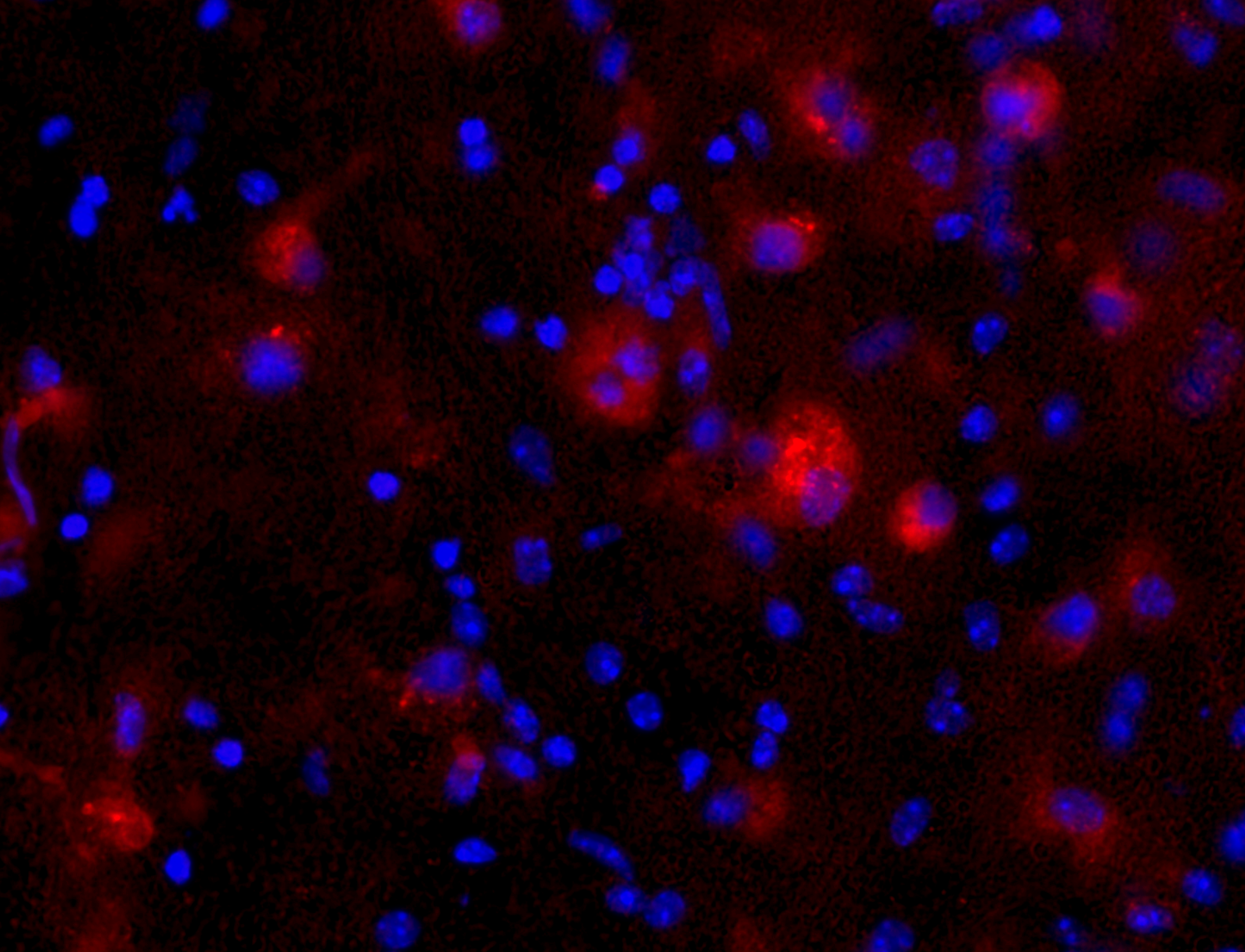

Supplement: Supplementary file 9 — Source data Fig. 6 [file 44321_2025_220_MOESM9_ESM.zip › EMM-2024-20638-V3_Figure 6/Figure 6G/WT MCAO/MERGE-WT-MCAO.png]

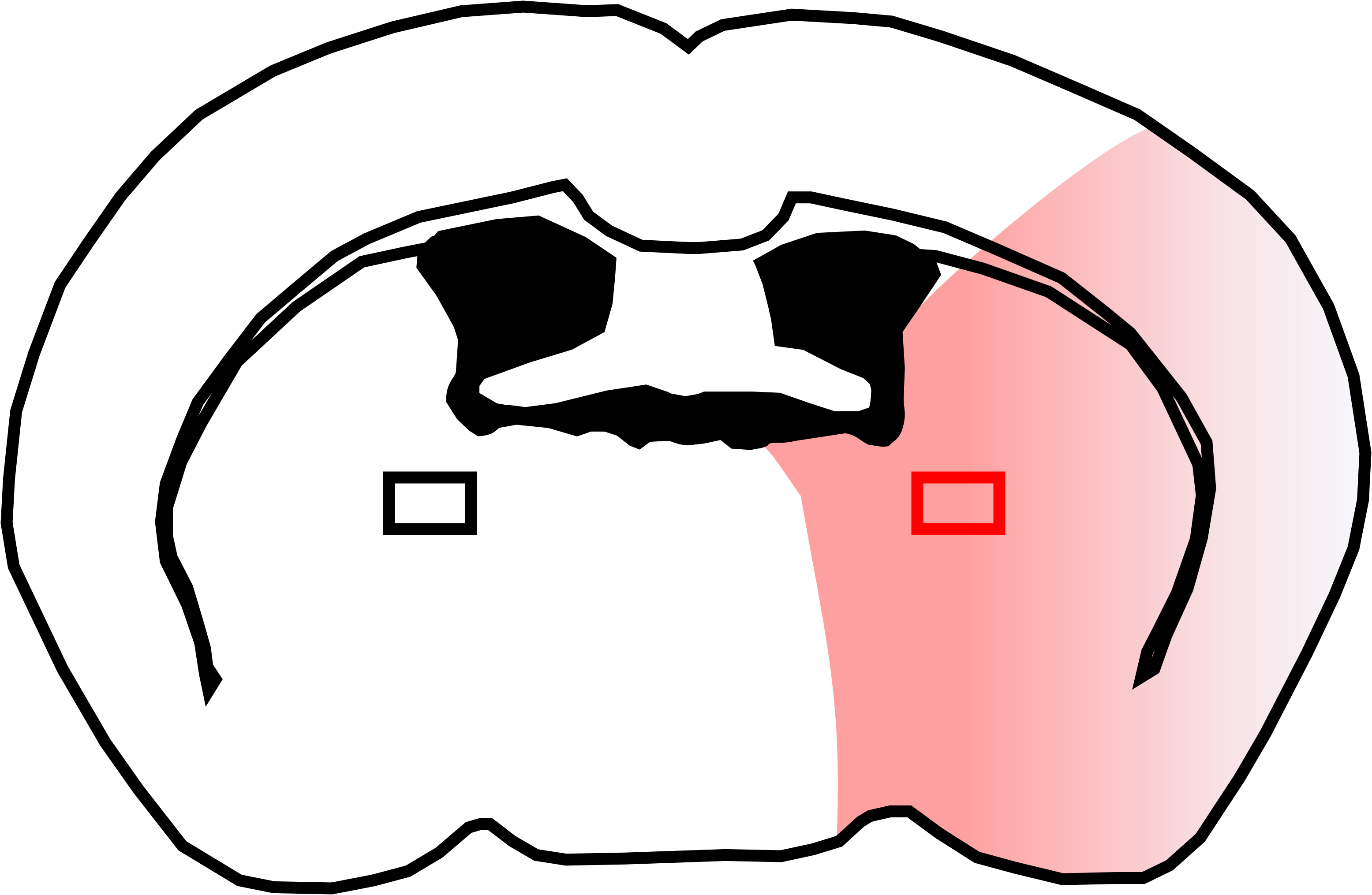

Supplement: Supplementary file 9 — Source data Fig. 6 [file 44321_2025_220_MOESM9_ESM.zip › EMM-2024-20638-V3_Figure 6/Figure 6H/Figure 6H.png]

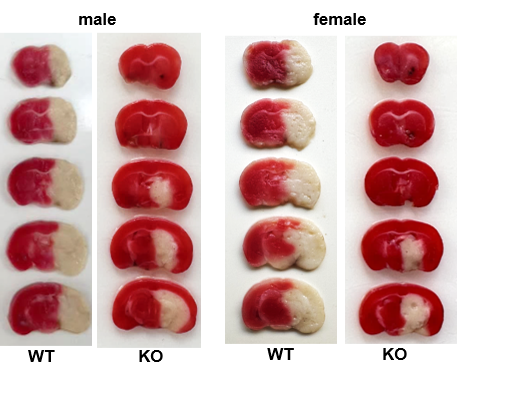

Supplement: Supplementary file 9 — Source data Fig. 6 [file 44321_2025_220_MOESM9_ESM.zip › EMM-2024-20638-V3_Figure 6/Figure 6J/Figure 6J.png]

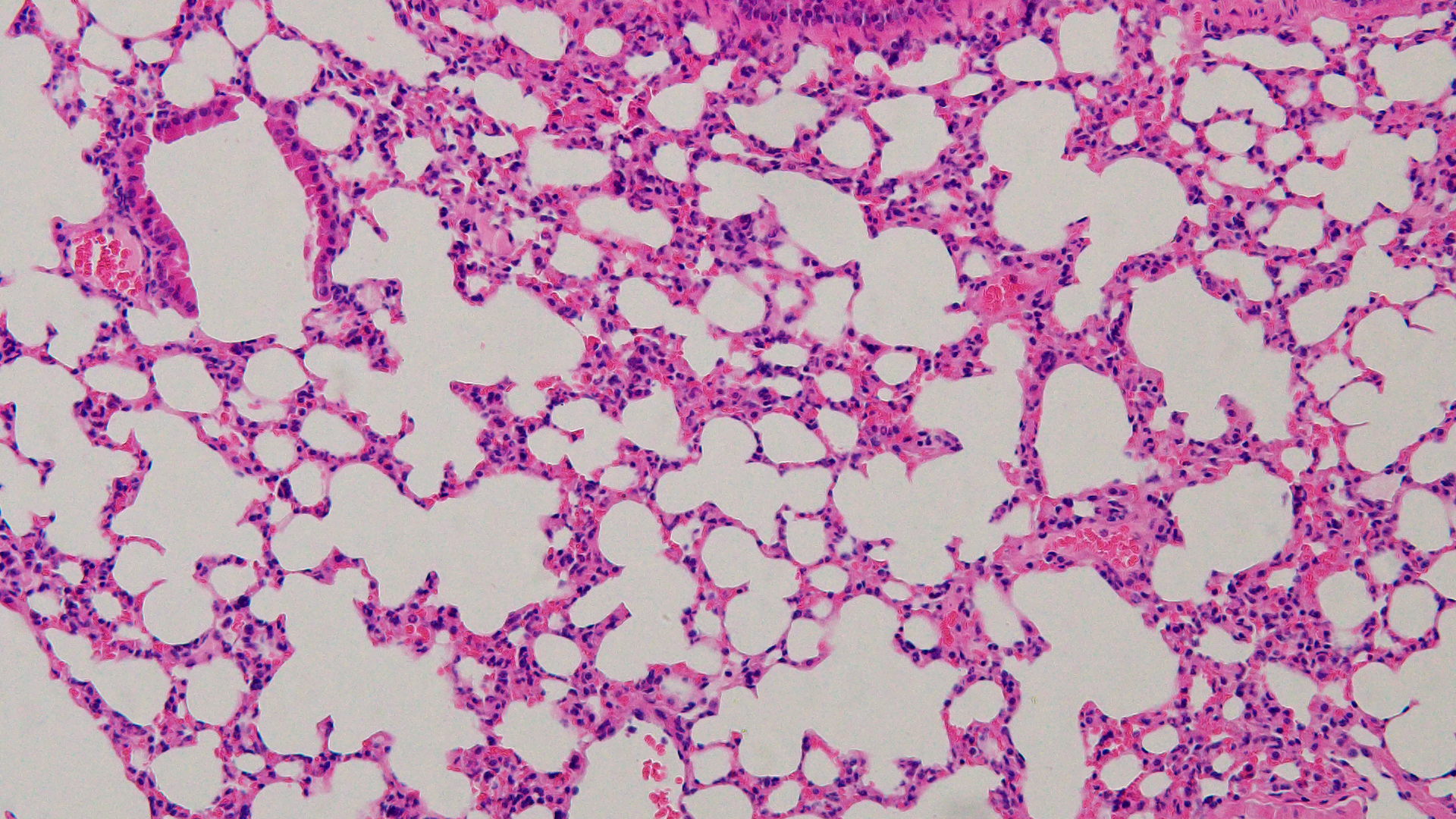

Supplement: Supplementary file 10 — Source data Fig. 7 [file 44321_2025_220_MOESM10_ESM.zip › Figure 7/Figure 7A/i-HPK1/i-HPK1.tif]

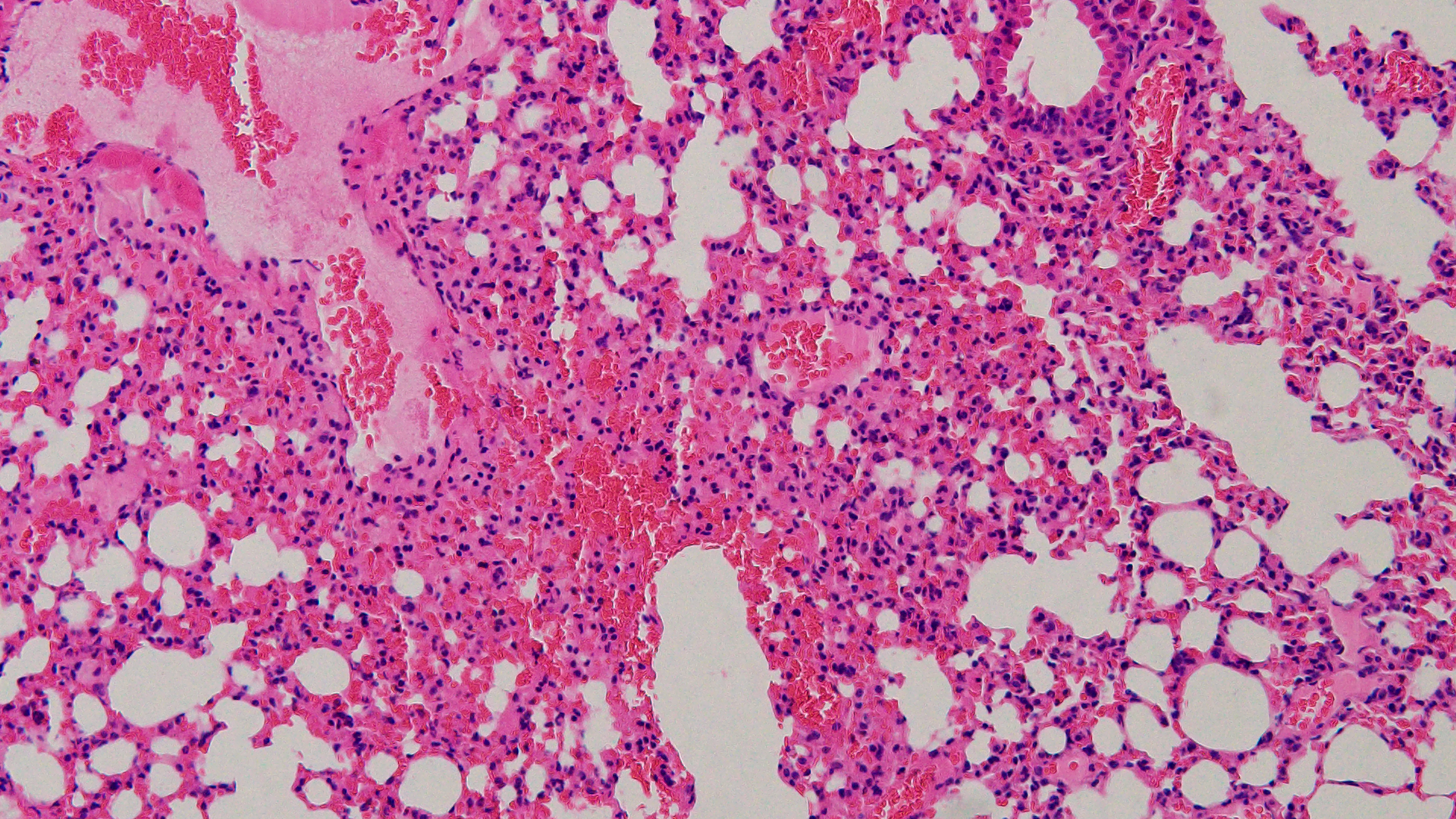

Supplement: Supplementary file 10 — Source data Fig. 7 [file 44321_2025_220_MOESM10_ESM.zip › Figure 7/Figure 7A/vehicle/vehicle.tif]

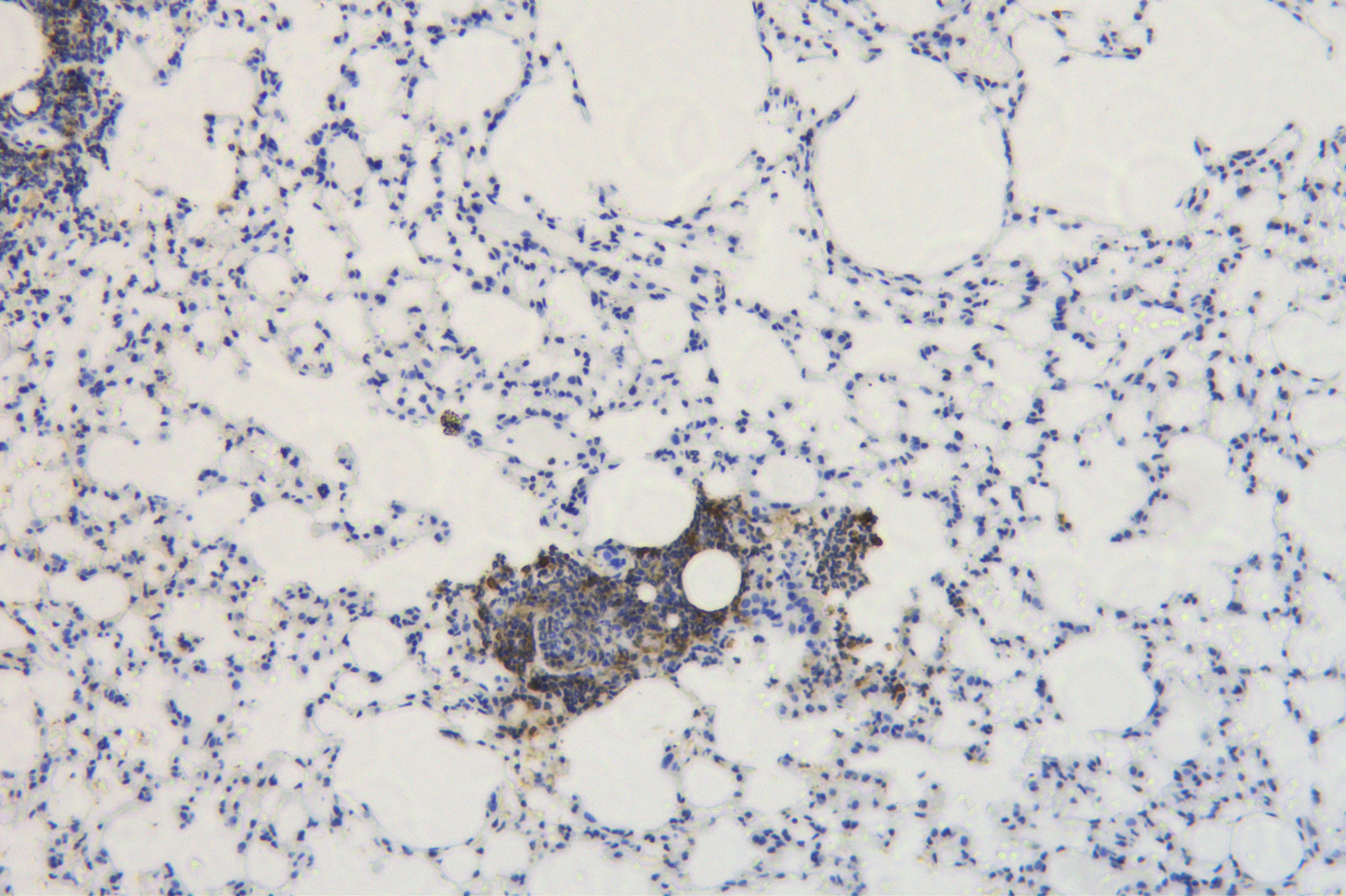

Supplement: Supplementary file 10 — Source data Fig. 7 [file 44321_2025_220_MOESM10_ESM.zip › Figure 7/Figure 7F/i-HPK1/i-HPK1.jpg]

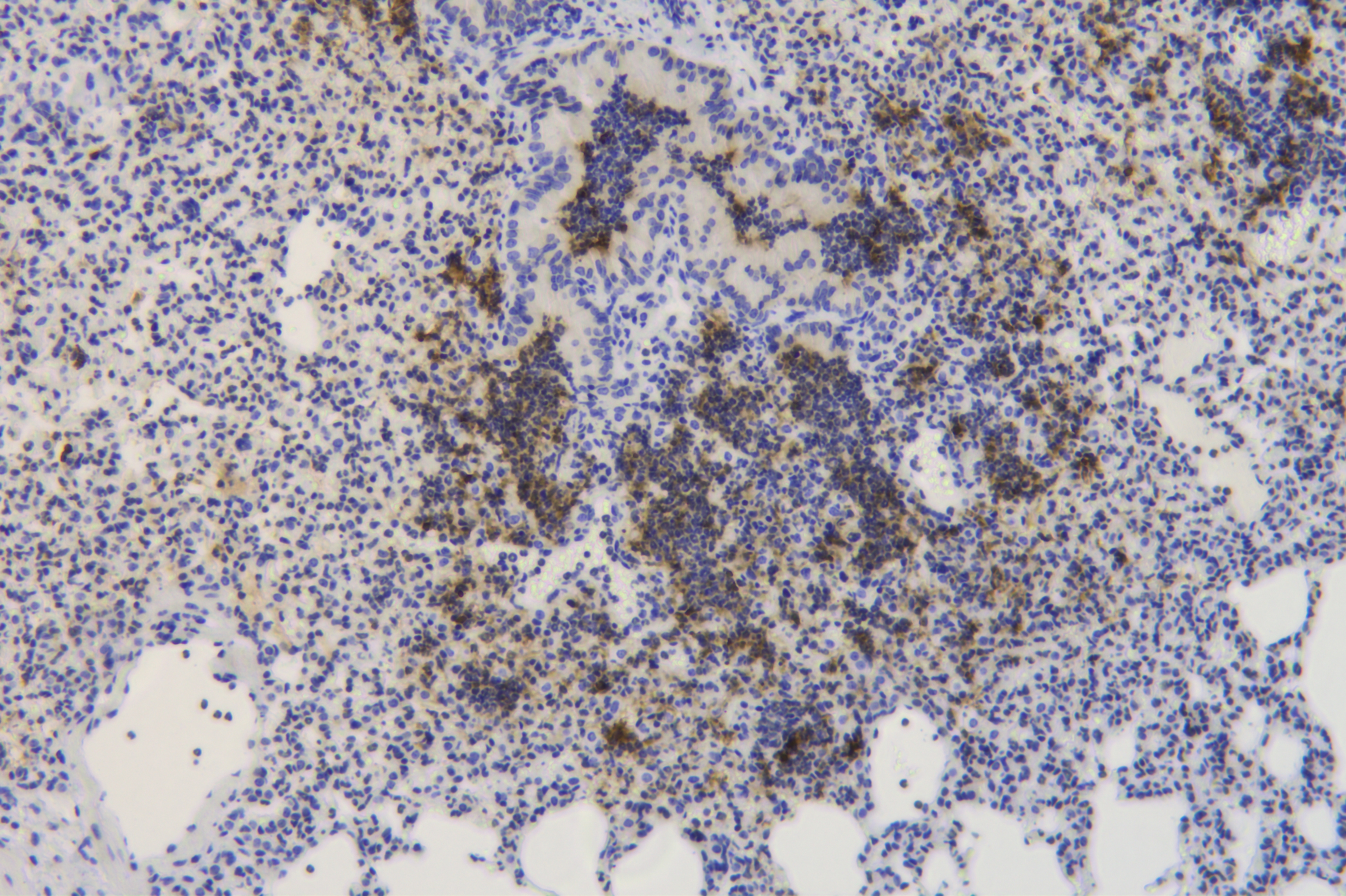

Supplement: Supplementary file 10 — Source data Fig. 7 [file 44321_2025_220_MOESM10_ESM.zip › Figure 7/Figure 7F/vehicle/vehicle.jpg]

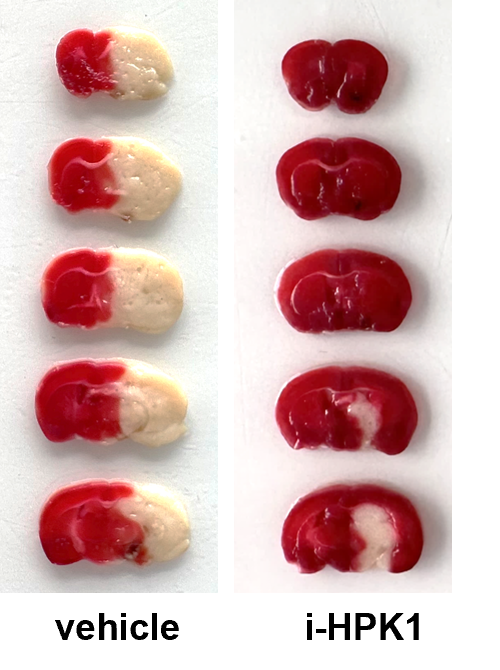

Supplement: Supplementary file 11 — Source data Fig. 8 [file 44321_2025_220_MOESM11_ESM.zip › Figure 8/Figure 8A/Figure 8A.png]

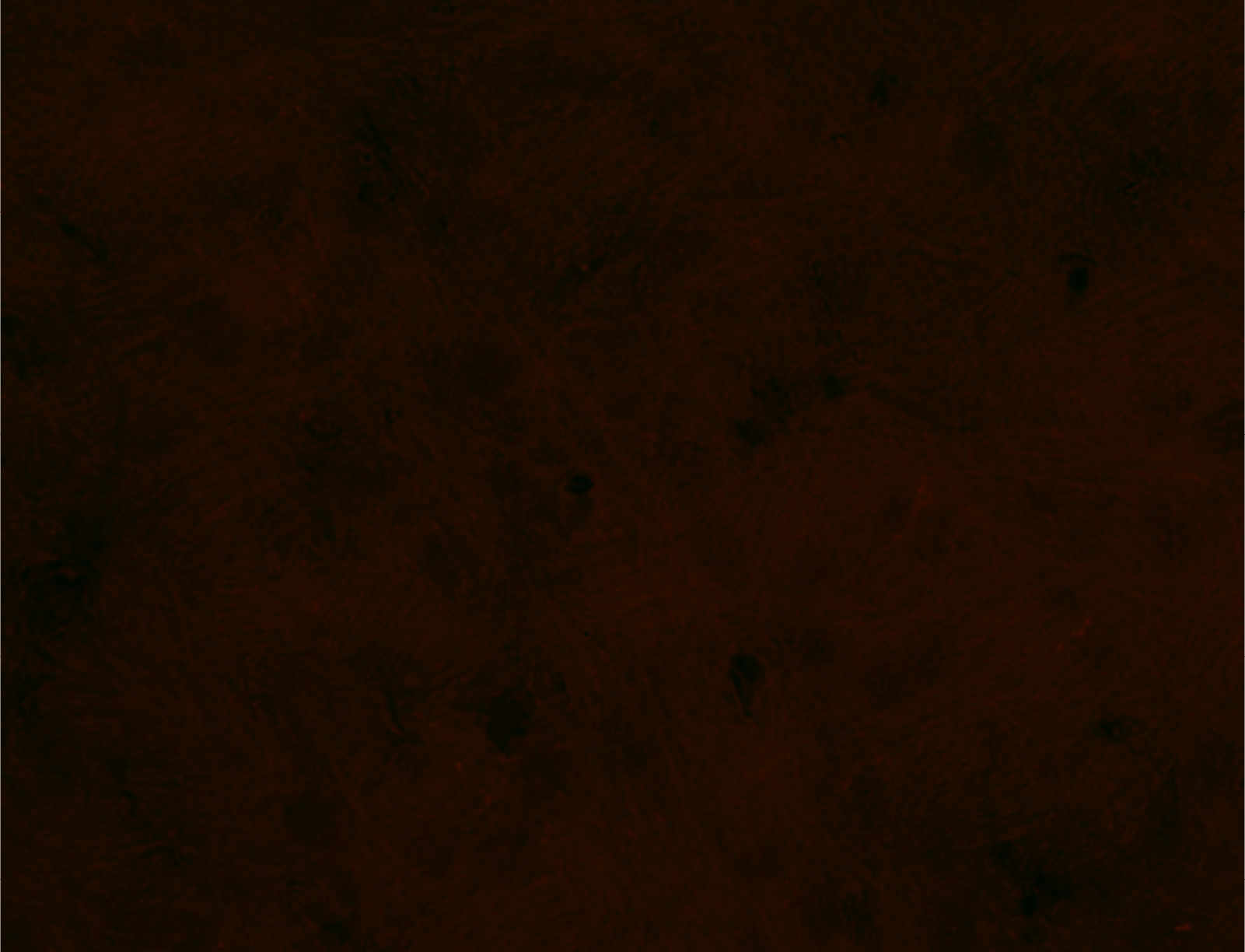

Supplement: Supplementary file 11 — Source data Fig. 8 [file 44321_2025_220_MOESM11_ESM.zip › Figure 8/Figure 8G/i-HPK1 control/CitH3-i-HPK1-control.png]

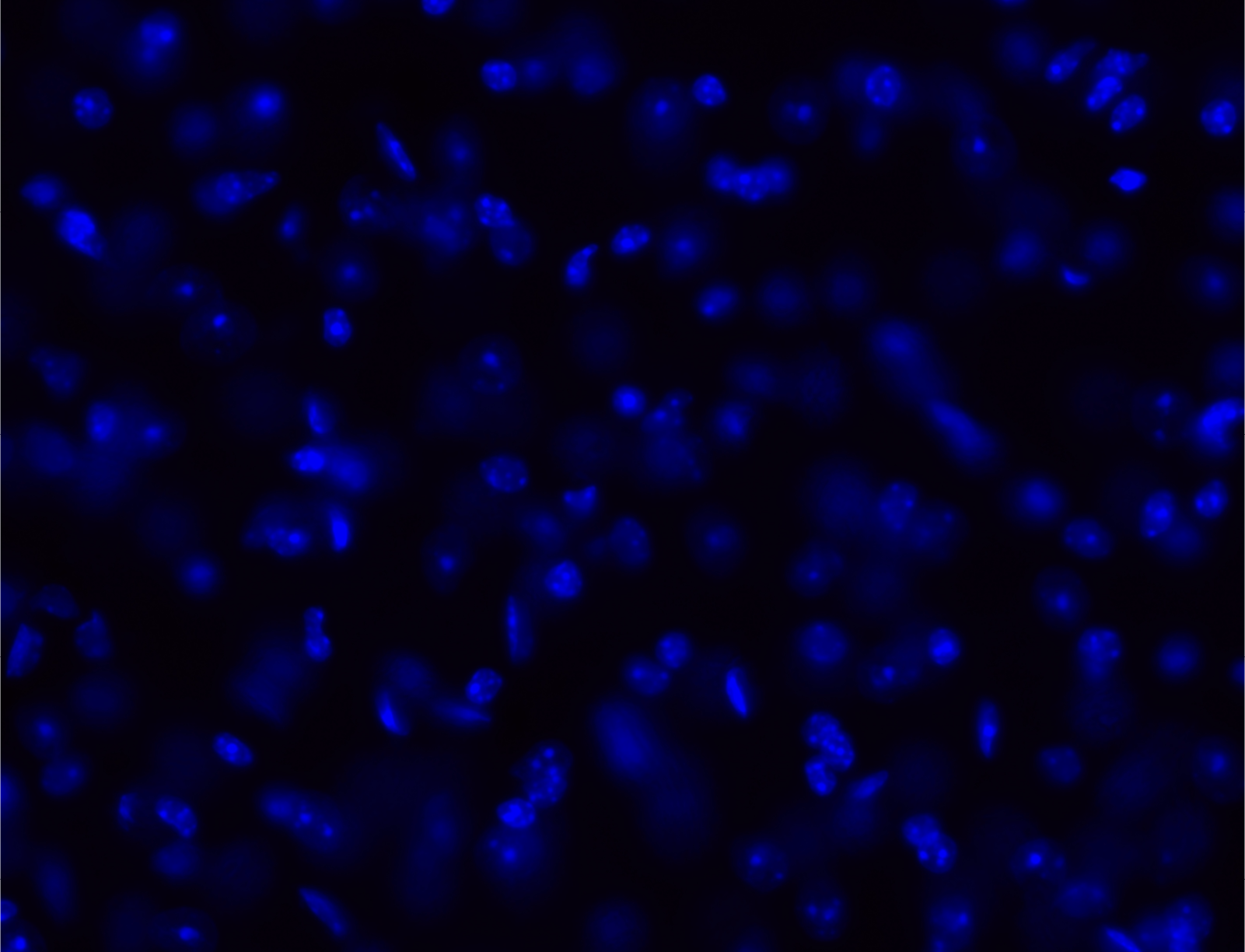

Supplement: Supplementary file 11 — Source data Fig. 8 [file 44321_2025_220_MOESM11_ESM.zip › Figure 8/Figure 8G/i-HPK1 control/DAPI-i-HPK1-control.png]

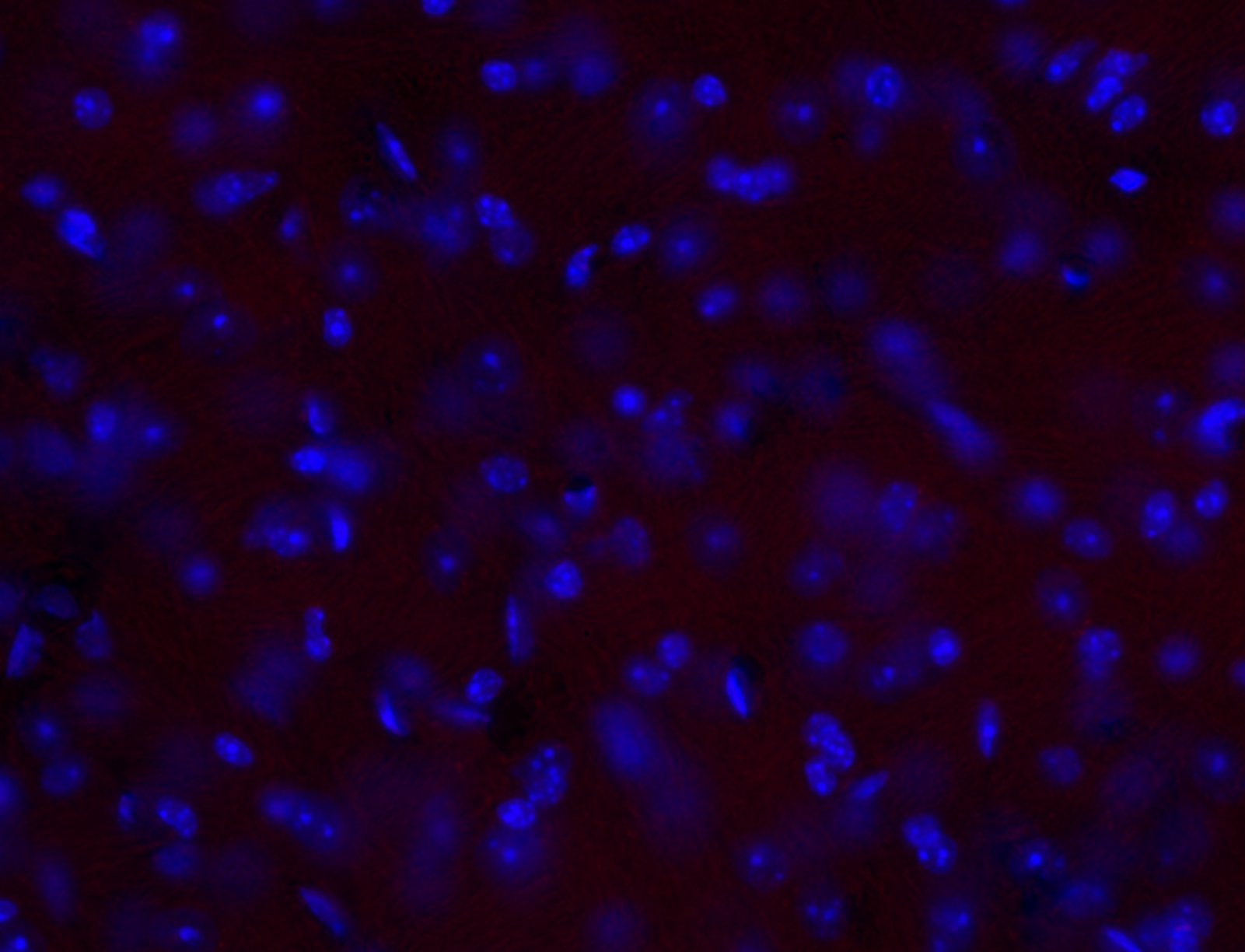

Supplement: Supplementary file 11 — Source data Fig. 8 [file 44321_2025_220_MOESM11_ESM.zip › Figure 8/Figure 8G/i-HPK1 control/MERGE-i-HPK1-control.png]

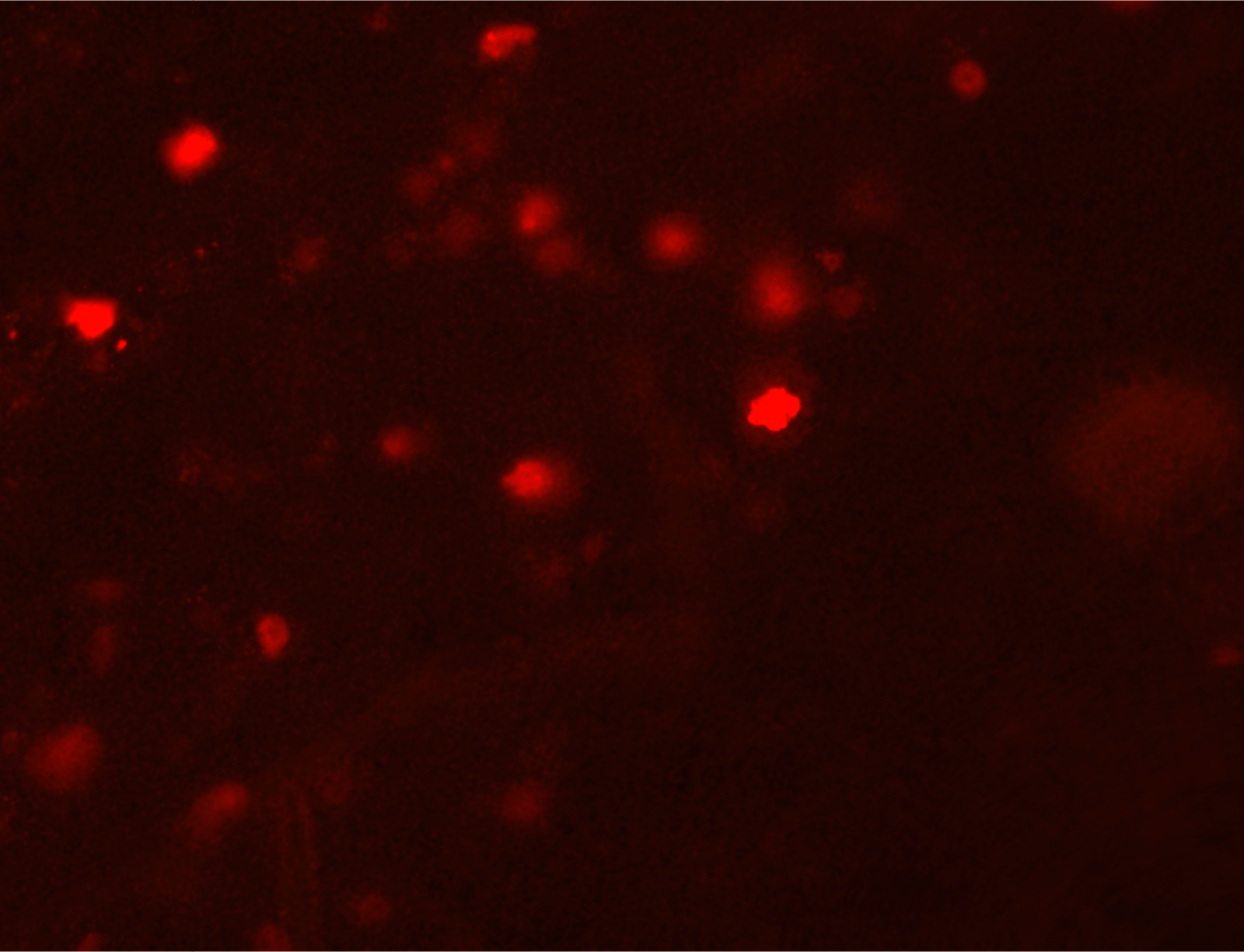

Supplement: Supplementary file 11 — Source data Fig. 8 [file 44321_2025_220_MOESM11_ESM.zip › Figure 8/Figure 8G/i-HPK1 MCAO/CitH3-i-HPK1-MCAO.png]

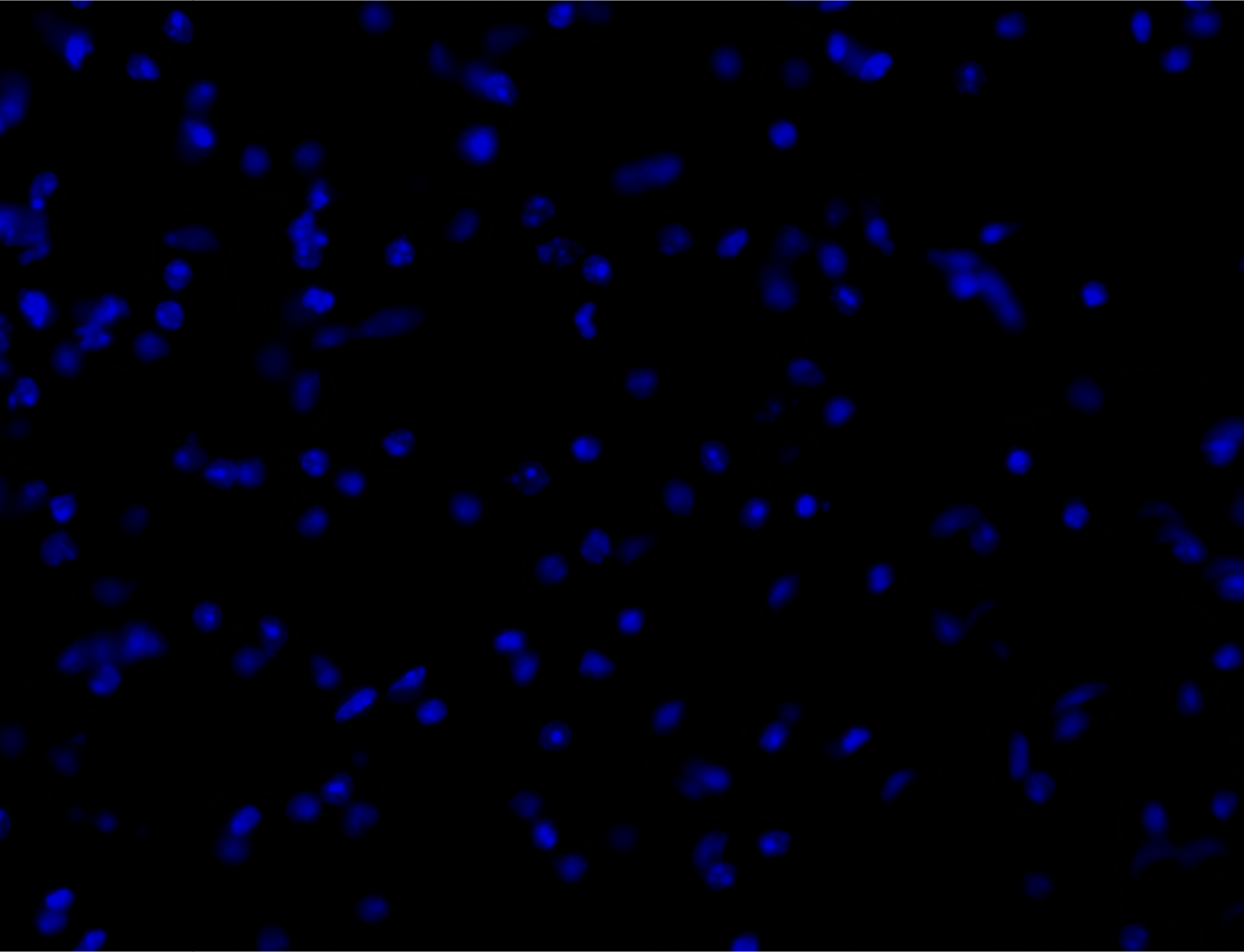

Supplement: Supplementary file 11 — Source data Fig. 8 [file 44321_2025_220_MOESM11_ESM.zip › Figure 8/Figure 8G/i-HPK1 MCAO/DAPI-i-HPK1-MCAO.png]

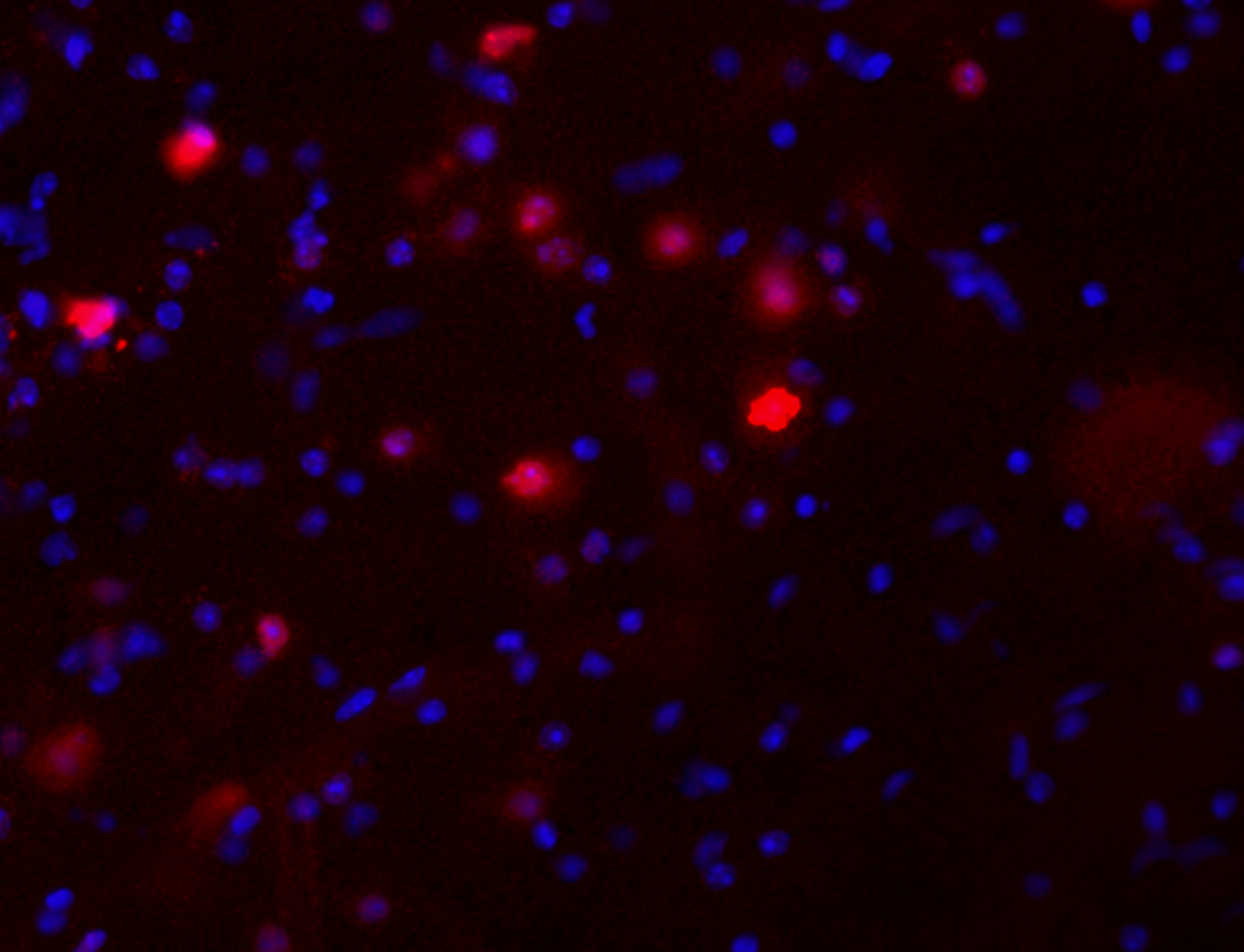

Supplement: Supplementary file 11 — Source data Fig. 8 [file 44321_2025_220_MOESM11_ESM.zip › Figure 8/Figure 8G/i-HPK1 MCAO/MERGE-i-HPK1-MCAO.png]

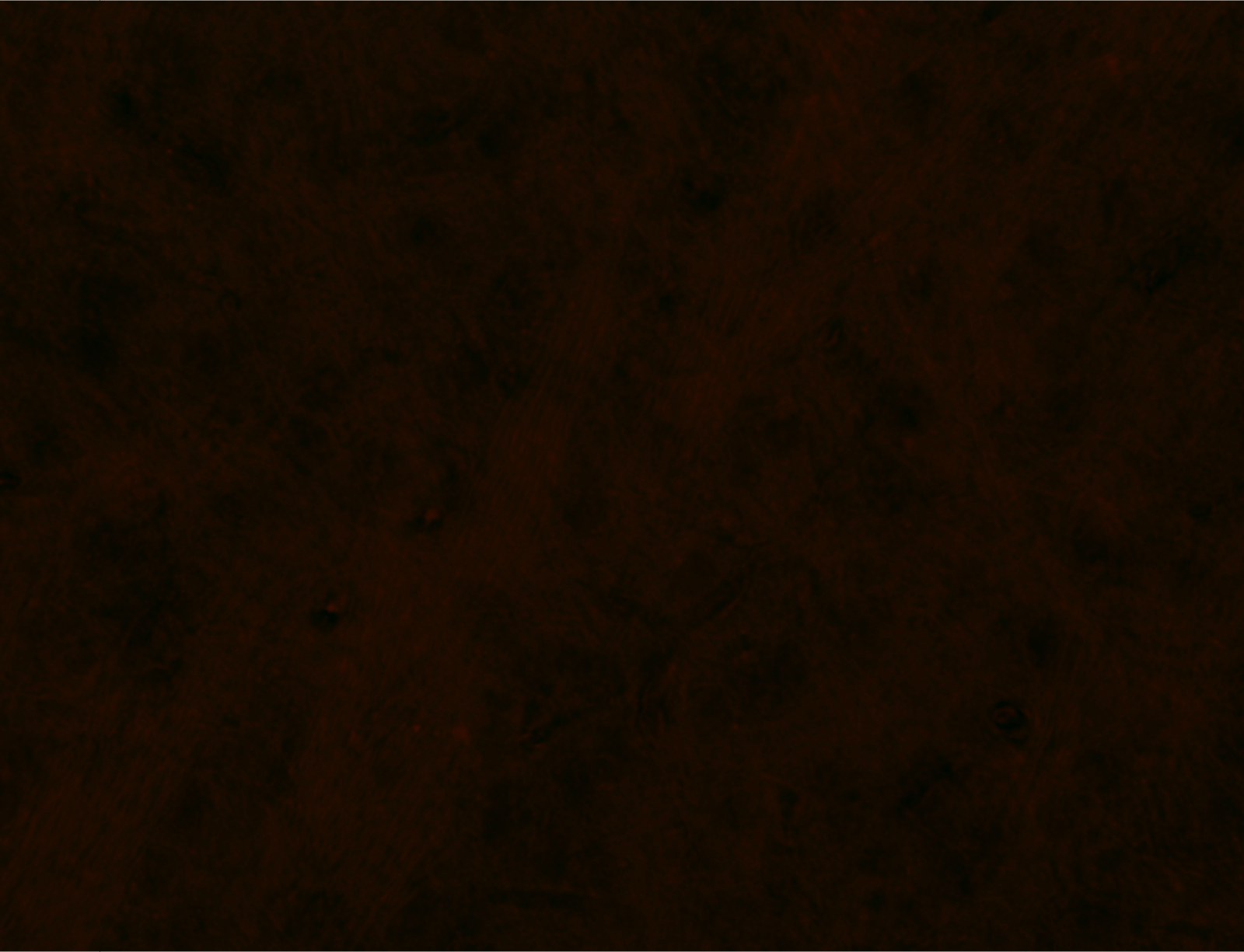

Supplement: Supplementary file 11 — Source data Fig. 8 [file 44321_2025_220_MOESM11_ESM.zip › Figure 8/Figure 8G/vehicle control/CitH3-vehicle-control.png]

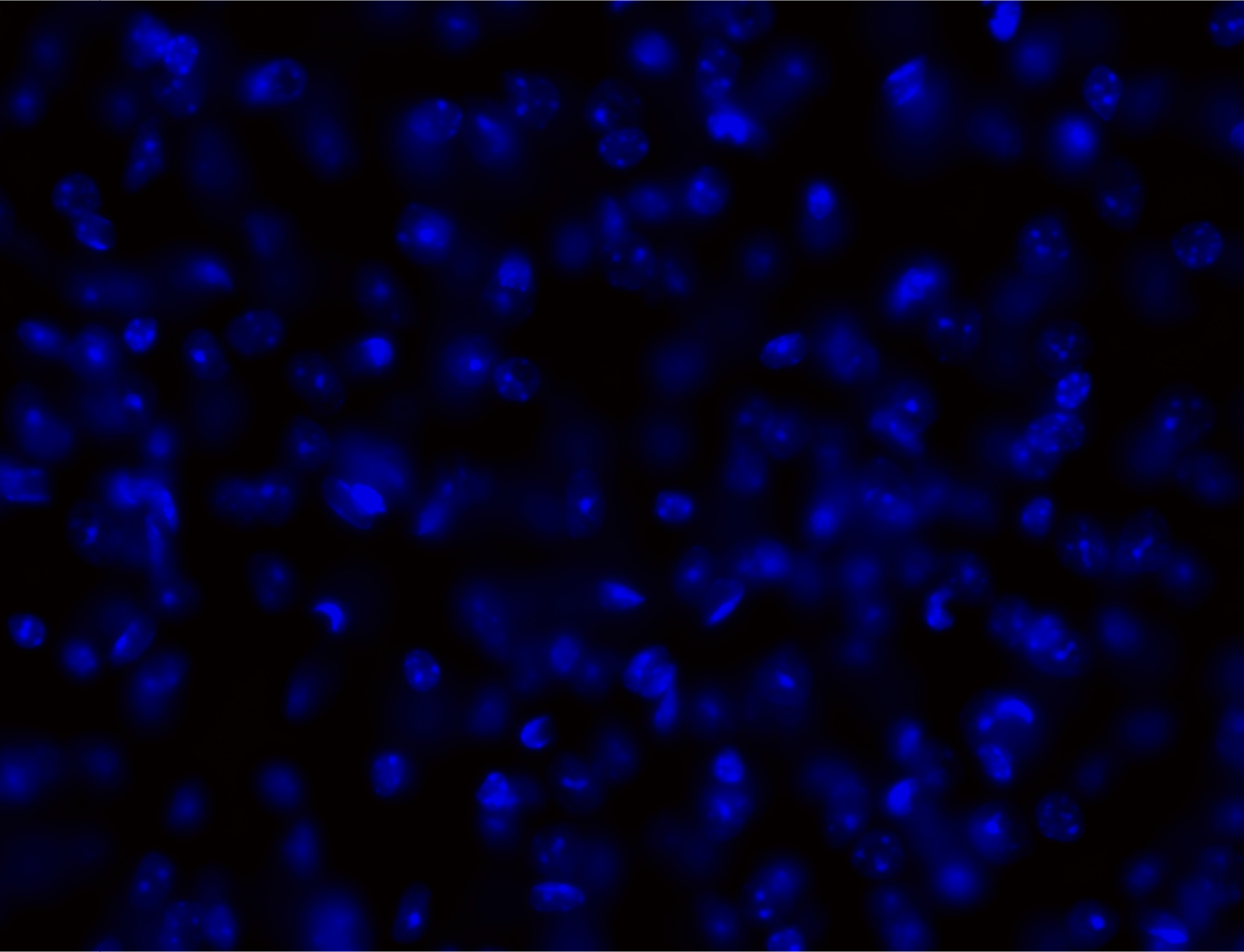

Supplement: Supplementary file 11 — Source data Fig. 8 [file 44321_2025_220_MOESM11_ESM.zip › Figure 8/Figure 8G/vehicle control/DAPI-vehicle-control.png]

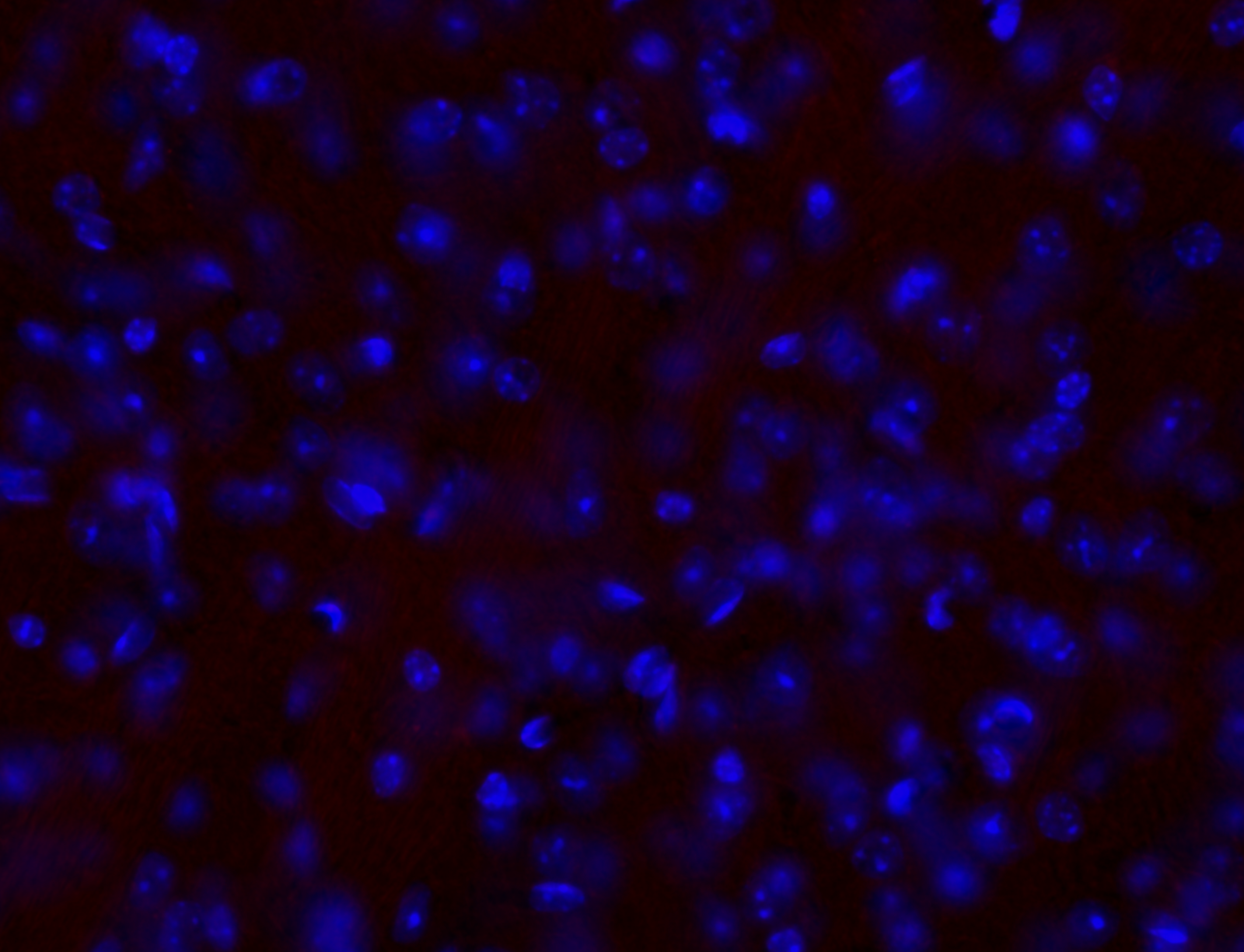

Supplement: Supplementary file 11 — Source data Fig. 8 [file 44321_2025_220_MOESM11_ESM.zip › Figure 8/Figure 8G/vehicle control/MERGE-vehicle-control.png]

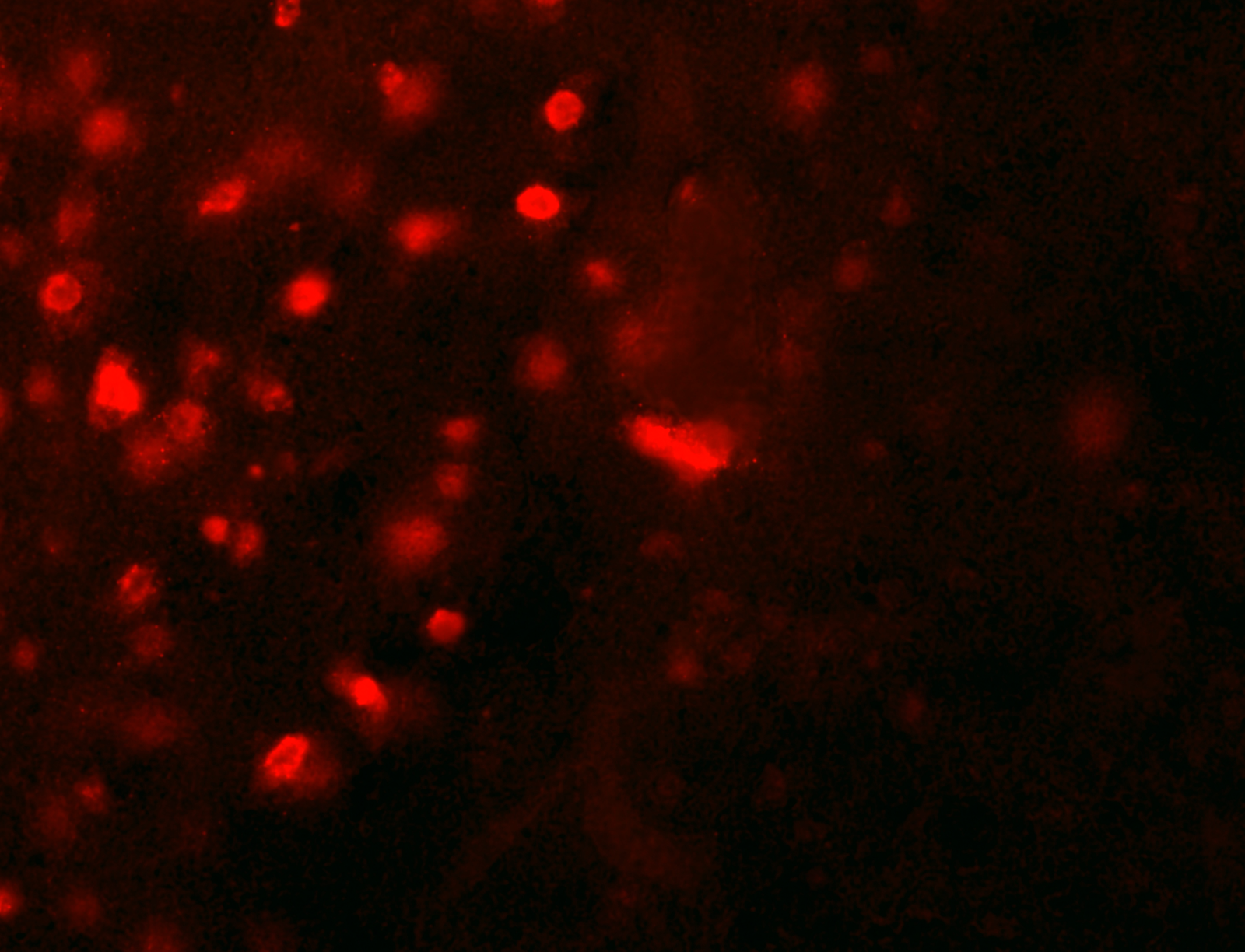

Supplement: Supplementary file 11 — Source data Fig. 8 [file 44321_2025_220_MOESM11_ESM.zip › Figure 8/Figure 8G/vehicle MCAO/CitH3-vehicle-MCAO.png]

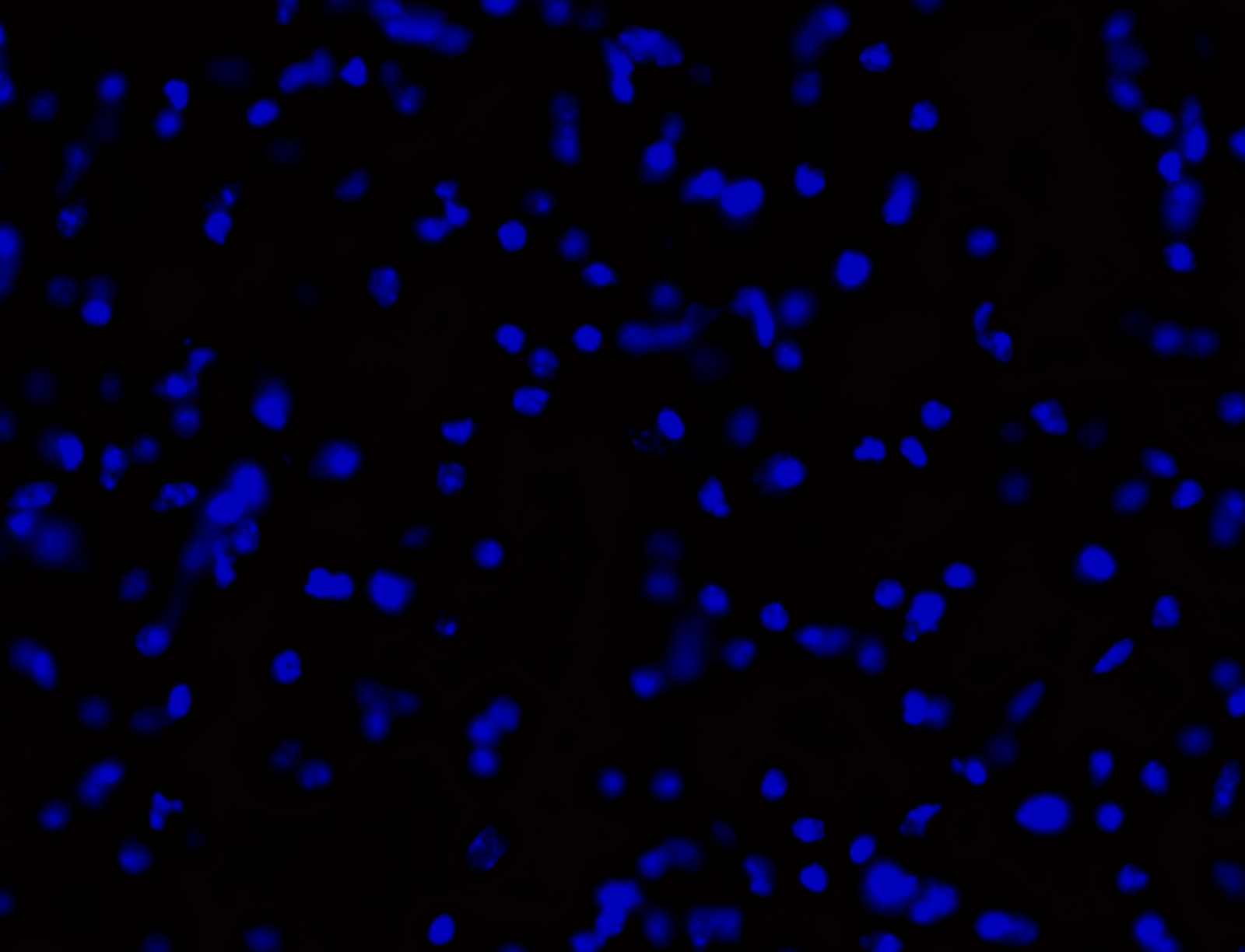

Supplement: Supplementary file 11 — Source data Fig. 8 [file 44321_2025_220_MOESM11_ESM.zip › Figure 8/Figure 8G/vehicle MCAO/DAPI-vehicle-MCAO.png]

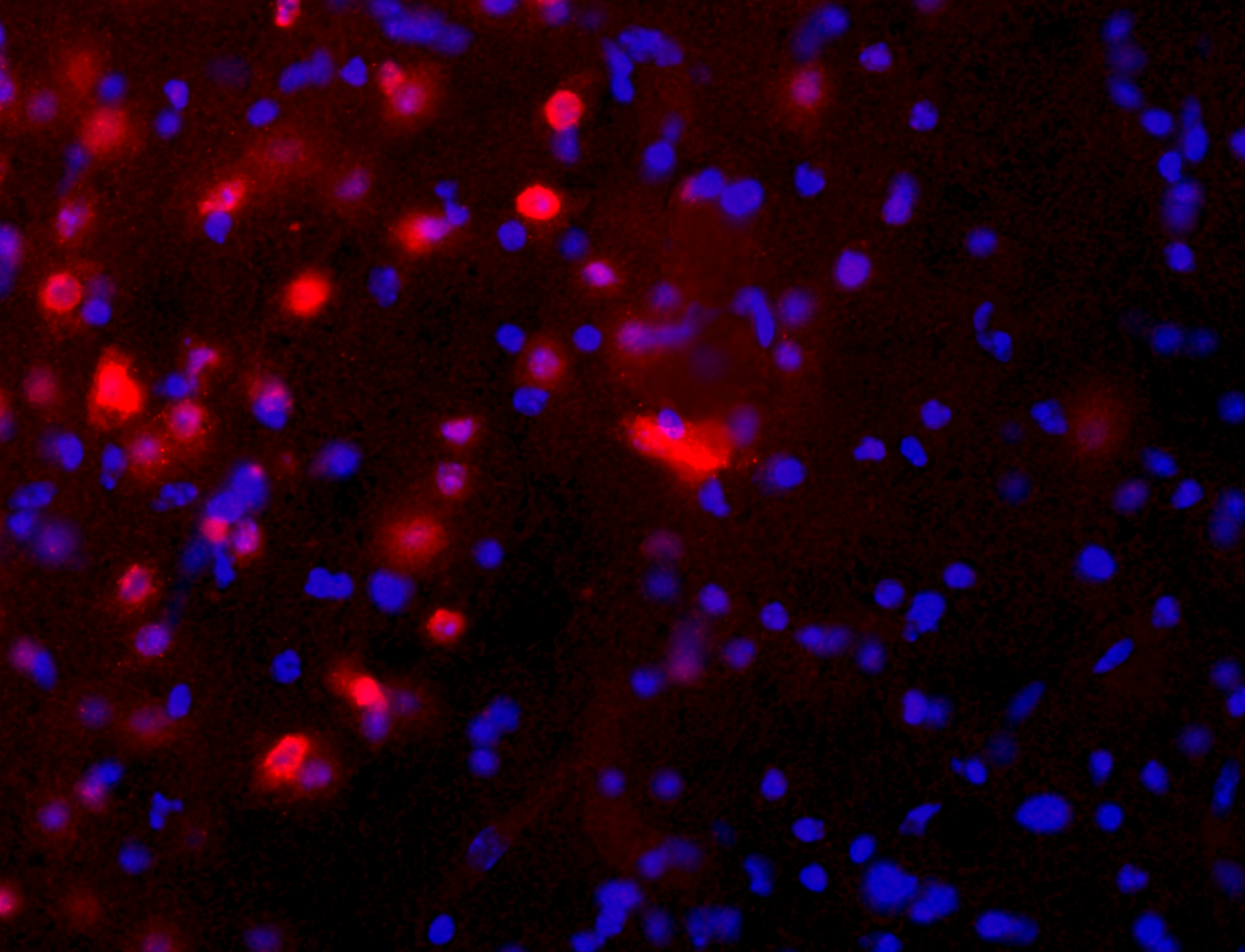

Supplement: Supplementary file 11 — Source data Fig. 8 [file 44321_2025_220_MOESM11_ESM.zip › Figure 8/Figure 8G/vehicle MCAO/MERGE-vehicle-MCAO.png]

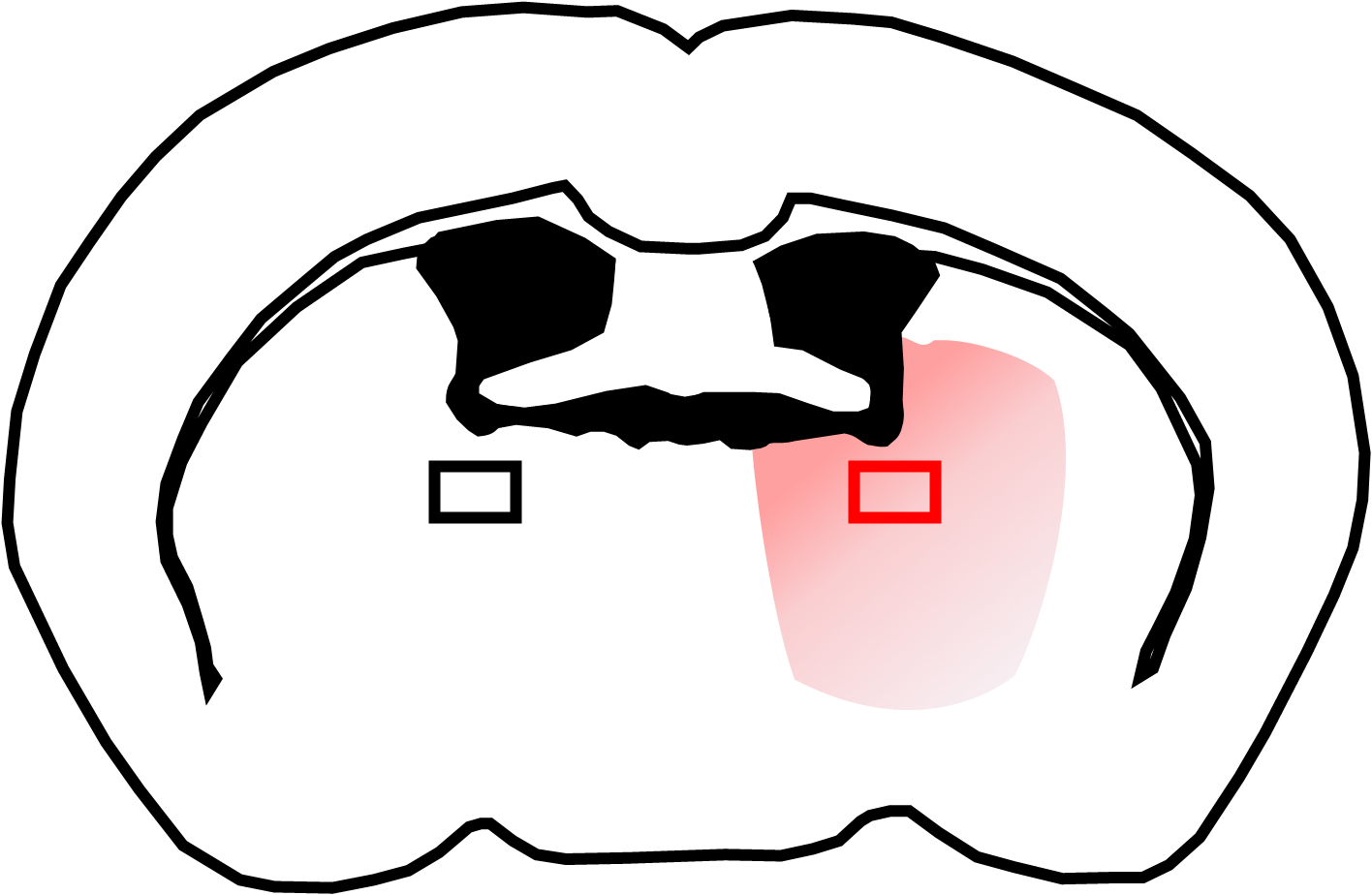

Supplement: Supplementary file 11 — Source data Fig. 8 [file 44321_2025_220_MOESM11_ESM.zip › Figure 8/Figure 8H/Figure 8H.png]
