# Supplementary material for: Targeting HPK1 inhibits neutrophil responses to mitigate post-stroke lung and cerebral injuries
Source: EMBO Mol Med. 2025 Apr 1;17(5):1018–40. doi: 10.1038/s44321-025-00220-8 (PMC12081623; doi:10.1038/s44321-025-00220-8)
Supplement: Supplementary file 12 — Expanded View Figures [file 44321_2025_220_MOESM12_ESM.pdf]

Expanded View Figures

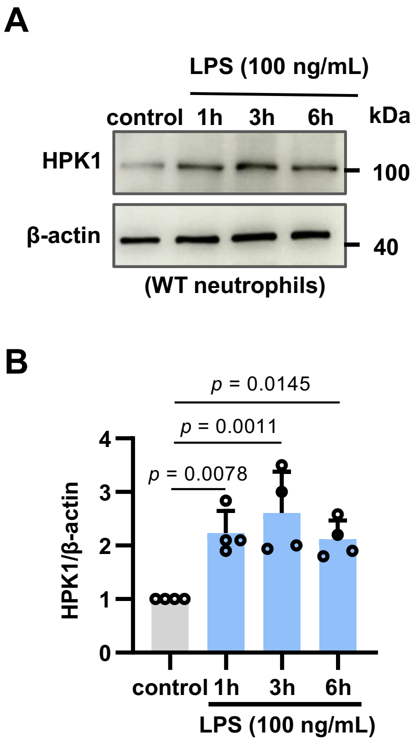

Figure EV1. HPK1 levels increase in primary neutrophils after LPS treatment.

(**A**, **B**) Representative HPK1 immunoblot (**A**) and quantification (**B**) in primary neutrophils from WT mice treated with LPS (100 ng/mL) for 1, 3, and 6 h. $N = 4$ per group. (**C**, **D**) Representative HPK1 immunoblot (**C**) and quantification (**D**) in primary neutrophils from WT mice treated with LPS (10 µg/mL) for 1 and 2 h. $N = 4$ per group. Bar graphs represent the mean ± SD. One-way ANOVA in (**B**) and (**D**). Source data are available online for this figure.

 © The Author(s)

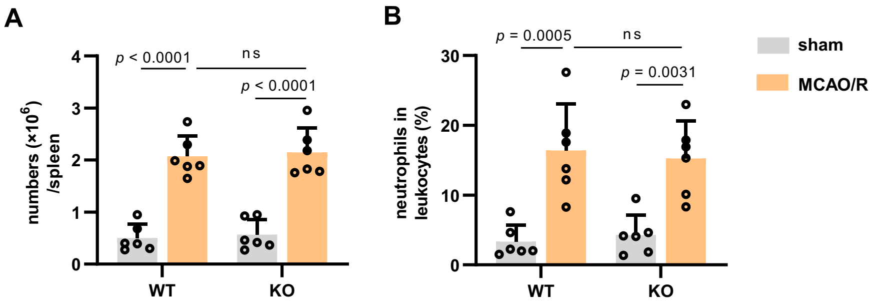

Figure EV2. HPK1 loss has no influence on neutrophil population in the mouse spleen after ischemic stroke.

(**A**, **B**) Flow cytometry analysis of the number (**A**) and frequency (**B**) of neutrophils in the spleen tissues of WT/KO mice after sham or MCAO/R for 24 h. $N = 6$ mice per group. Bar graphs represent the mean ± SD. ns, no significance. Two-way ANOVA in (**A**) and (**B**). Source data are available online for this figure.

© The Author(s)

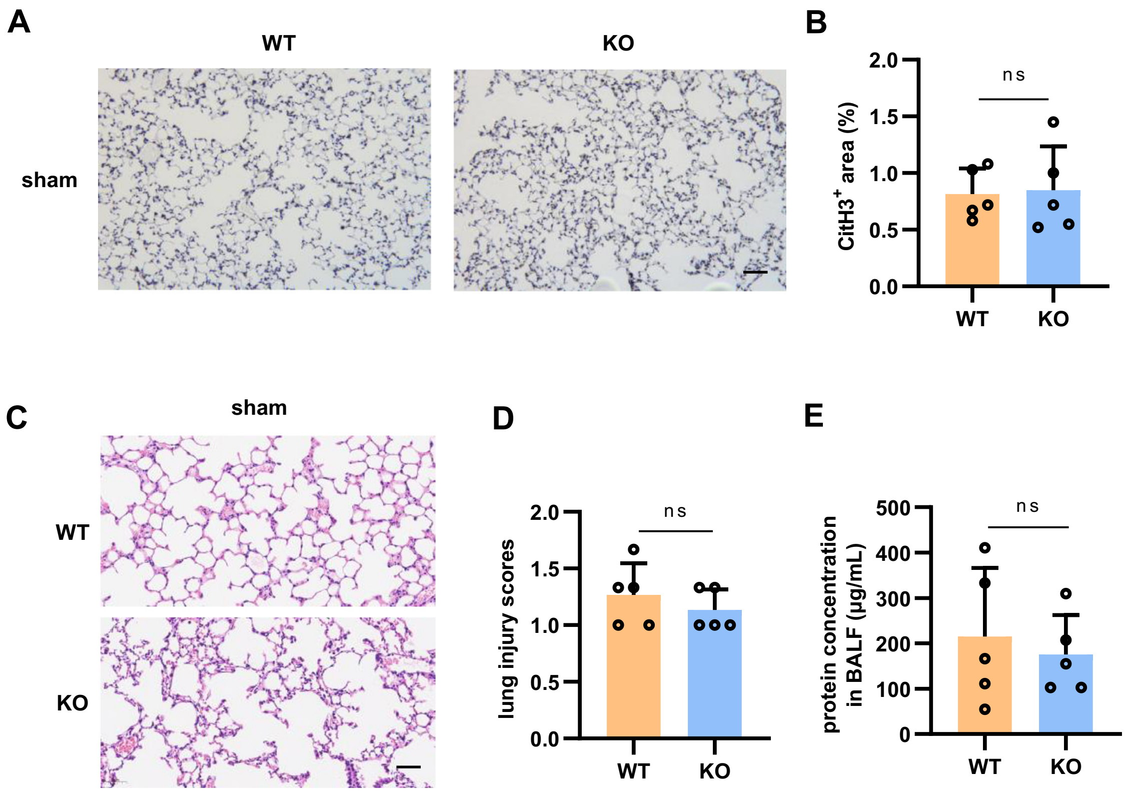

Figure EV3. There are no marked NETs and lung injury in WT and HPK1 loss mice after sham operation.

(**A**, **B**) Representative CitH3 immunohistochemical images (**A**) and quantification (**B**) in the lung sections from WT/KO mice after sham operation. *N* = 5 mice per group. Scale bar = 50 μm. (**C**, **D**) Representative HE staining (**C**) and histopathological scores (**D**) of lung sections from WT/KO mice after sham operation. Scale bar = 50 μm. *N* = 5 mice per group. (**E**) Protein concentration of BALF in WT/KO mice after sham operation. *N* = 5 mice per group. Bar graphs represent the mean ± SD. ns, no significance. Unpaired Student's *t*-test in (**B**), (**D**), and (**E**). Source data are available online for this figure.

 © The Author(s)

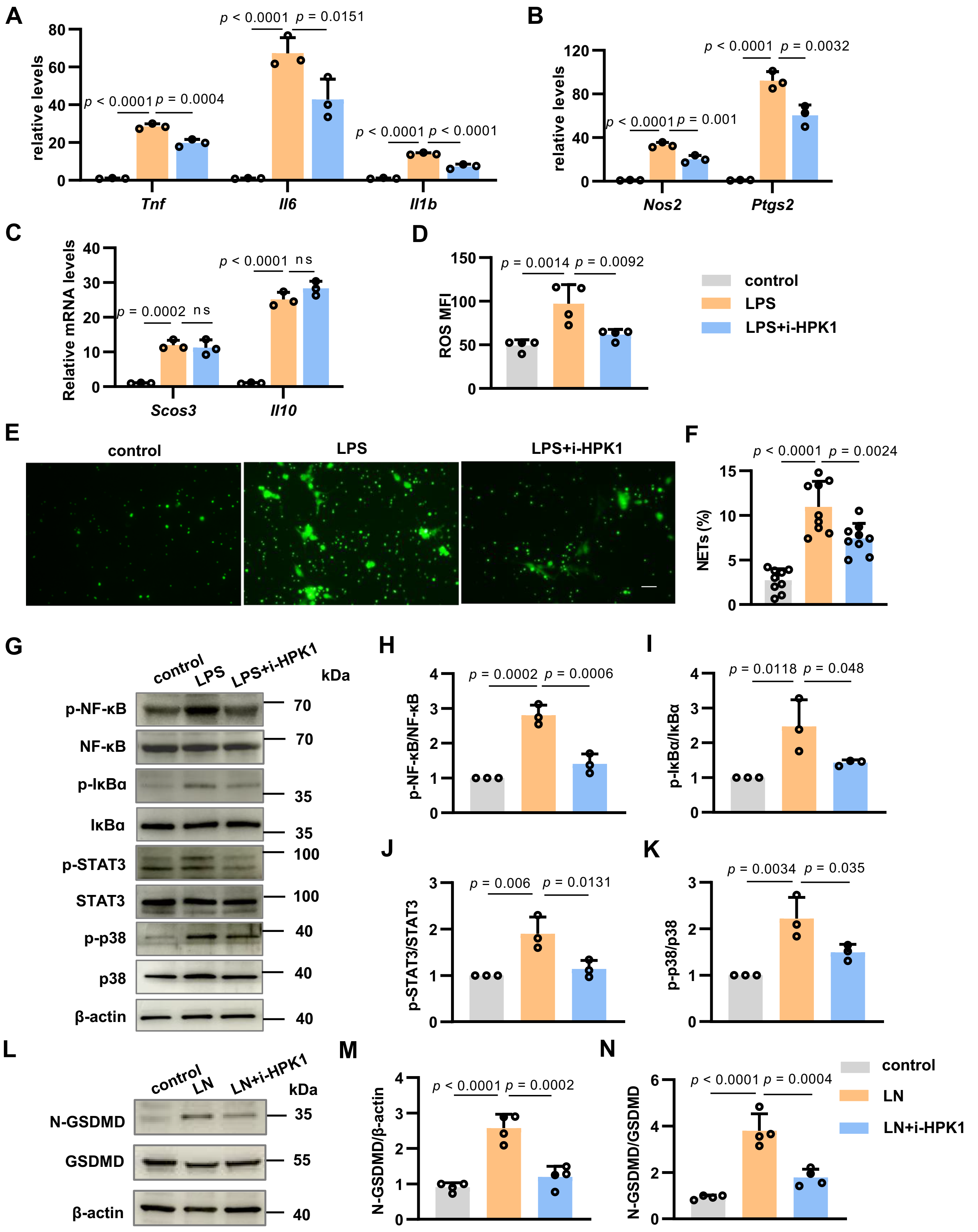
A
relative levels
p < 0.0001 p = 0.0004
p < 0.0001 p = 0.0151
p < 0.0001 p < 0.0001
Tnf
Il6
Il1b
B
relative levels
p < 0.0001 p = 0.001
p < 0.0001 p = 0.0032
Nos2
Ptgs2
C
Relative mRNA levels
p = 0.0002 ns
p < 0.0001 ns
Scos3
Il10
D
ROS MFI
p = 0.0014 p = 0.0092
control
LPS
LPS+i-HPK1
E
control
LPS
LPS+i-HPK1
F
NETs (%)
p < 0.0001 p = 0.0024
G
control LPS LPS+i-HPK1
kDa
p-NF-κB
NF-κB
p-IκBα
IκBα
p-STAT3
STAT3
p-p38
p38
β-actin
H
p-NF-κB/NF-κB
p = 0.0002 p = 0.0006
I
p-IκBα/IκBα
p = 0.0118 p = 0.048
J
p-STAT3/STAT3
p = 0.006 p = 0.0131
K
p-p38/p38
p = 0.0034 p = 0.035
L
control LN LN+i-HPK1
kDa
N-GSDMD
GSDMD
β-actin
M
N-GSDMD/β-actin
p < 0.0001 p = 0.0002
N
N-GSDMD/GSDMD
p < 0.0001 p = 0.0004
control
LN
LN+i-HPK1

Figure EV4. Inhibiting HPK1 reduces LPS-induced hyperactivity and NF-κB/STAT3/p38/GSDMD pathways in primary neutrophils.

(**A–C**) qPCR analysis of *Tnf*, *Il6*, and *Il1b* (**A**), *Nos2* and *Ptgs2* (**B**), *Socs3* and *Il10* (**C**) in WT neutrophils treated with LPS (100 ng/mL). $N = 3$ per group, three-time repeats. (**D**) Flow cytometry analysis of ROS levels (MFI) in WT neutrophils treated with LPS (100 ng/mL) for 3 h. $N = 4$ per group, three-time repeats. (**E–F**) Representative fluorescence imaging (**E**) and quantification (**F**) of NET formation. Neutrophils were stimulated with LPS (10 μg/mL) for 2.5 h, and NETs were visualized using SYTOX Green. Scale bar = 50 μm. $N = 9$ per group. (**G–K**) Representative immunoblot (**G**) of phosphorylation and total protein levels of NF-κB (**H**), IκBα (**I**), STAT3 (**J**), and p38 (**K**) in WT neutrophils treated with LPS (100 ng/mL). $N = 3$ per group. (**L–N**) Representative immunoblot (**L**) and quantification of GSDMD cleavage (N-GSDMD) levels (**M**) and N-GSDMD/GSDMD ratio (**N**) in WT neutrophils treated with LPS (500 ng/mL) and nigericin (LN). $N = 4$ per group. i-HPK1 was treated 30 min before LPS treatment. Bar graphs represent the mean ± SD. ns, no significance. One-way ANOVA in (**A–D**), (**F**), (**H–K**), (**M**), and (**N**). Source data are available online for this figure.

© The Author(s)

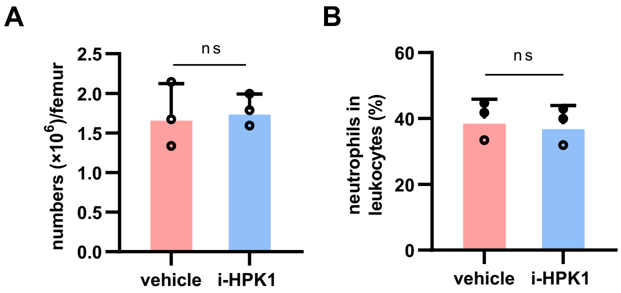

Figure EV5. Inhibition of HPK1 has no influence on neutrophil release from bone marrow in mice after ischemic stroke.

(**A**, **B**) Flow cytometry analysis of the number (**A**) and frequency (**B**) of bone marrow neutrophils in mice after MCAO/R for 24 h. Mice were treated with vehicle or i-HPK1 30 min after reperfusion following MCAO (90 min). $N = 3$ mice per group. Bar graphs represent the mean ± SD. ns, no significance. Unpaired Student's *t*-test in (**A**) and (**B**). Source data are available online for this figure.

© The Author(s)
